# Phenothiazine‐Functionalized Poly(norbornene)s as High‐Rate Cathode Materials for Organic Batteries

**DOI:** 10.1002/cssc.201903168

**Published:** 2020-01-28

**Authors:** Fabian Otteny, Gauthier Studer, Martin Kolek, Peter Bieker, Martin Winter, Birgit Esser

**Affiliations:** ^1^ Institute for Organic Chemistry University of Freiburg Albertstraße 21 79104 Freiburg Germany; ^2^ Freiburg Materials Research Center University of Freiburg Stefan-Meier-Str. 21 79104 Freiburg Germany; ^3^ MEET Battery Research Center Institute of Physical Chemistry University of Münster Corrensstraße 46 48149 Münster Germany; ^4^ Helmholtz Institute Münster (HI MS), IEK-12 Forschungszentrum Jülich GmbH Corrensstraße 46 48149 Münster Germany; ^5^ Cluster of Excellence livMatS @ FIT-Freiburg Center for Interactive Materials and Bioinspired Technologies University of Freiburg Georges-Köhler-Allee 105 79110 Freiburg Germany

**Keywords:** fast charging, high rate capability, organic batteries, phenothiazine, redox polymers

## Abstract

Organic cathode materials are handled as promising candidates for new energy‐storage solutions based on their transition‐metal‐free composition. Phenothiazine‐based polymers are attractive owing to their redox potential of 3.5 V vs. Li/Li^+^ and high cycling stabilities. Herein, three types of poly(norbornene)s were investigated, functionalized with phenothiazine units through either a direct connection or ester linkages, as well as their crosslinked derivatives. The directly linked poly(3‐norbornylphenothiazine)s demonstrated excellent rate capability and cycling stability with a capacity retention of 73 % after 10 000 cycles at a C‐rate of 100 C for the crosslinked polymer. The polymer network structure of the crosslinked poly(3‐norbornylphenothiazine) was beneficial for its rate performance.

## Graphical abstract

**Fast charging**: Poly(norbornene)s, functionalized with phenothiazine units through either a direct connection or ester linkages, are tested as cathode‐active battery materials. Directly linked poly(3‐norbornylphenothiazine) demonstrates excellent rate capability and cycling stability with a capacity retention of 73 % after 10 000 cycles at a C‐rate of 100 C.

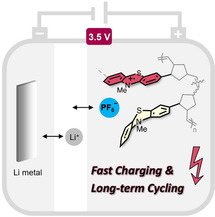

In view of the growing need for energy storage, the development of new battery technologies is necessary. Organic compounds are promising candidates because of their heavy‐metal‐ and transition‐metal‐free composition.^1^, ^2^, ^3^, ^4^ Furthermore, organic electrodes can be mechanically flexible, an advantage for application in consumer products such as wearable electronics or flexible displays.^5^, ^6^ The manifold possibilities for the design of organic compounds offer a broad diversity of structural, mechanical, and electrochemical properties.^7^ The redox properties of organic compounds can be easily modulated through incorporation of functional groups, thus enabling a customized battery voltage.^2^ However, many organic materials suffer from insufficient cycling stabilities owing to dissolution into liquid electrolytes and from low rate capabilities owing to their poor electronic conductivity.^8^, ^9^, ^10^ To improve the latter, a carbon‐based conductive additive is usually added to the electrode, and solubility can be suppressed by incorporating redox‐active moieties into polymeric architectures.^8^, ^11^ It is important, however, to consider the insolubility of the redox polymer in both its neutral and charged state, and in some cases crosslinking is required.^12^, ^13^ The redox‐active unit determines the charge/discharge potential of the battery. Using organic redox polymers, discharge voltages of up to 4.1 V vs. Li/Li^+^ have been reported.^14^ Radical polymers containing stable nitroxide radicals are among the most investigated organic electrode materials.^15^, ^16^, ^17^, ^18^, ^19^ Various backbones have been studied, including poly(methacrylate)s, poly(vinyl ether)s, poly(styrene)s, and poly(norbornene)s.^17^ The most well‐known example is PTMA [poly(2,2,6,6‐tetramethylpiperidinyloxy‐4‐ylmethacrylate)]. Its oxidation potential of 3.6 V vs. Li/Li^+^ lies close to the operating potential of commercial Li‐ion battery cells.^20^ The charge/discharge reaction of these and other p‐type polymers for Li–organic batteries proceeds according to dual‐ion cells, that is, involves the insertion and de‐insertion of electrolyte anions.^21^ Another redox‐active group with a similar oxidation potential is phenothiazine (PT). We,^12^, ^13^, ^22^, ^23^ among other groups,^24^, ^25^, ^26^, ^27^ have investigated PT‐based polymers as cathode materials. In particular poly(3‐vinyl‐*N*‐methylphenothiazine) (PVMPT) showed excellent results regarding cycling stability and rate capability.^12^, ^13^, ^22^ For the non‐crosslinked derivative, however, strong π‐interactions led to only half of the theoretical specific capacity being accessible.^12^, ^22^ These interactions occurred partly intramolecularly between neighboring units along the poly(vinylene) chain. The close intramolecular vicinity of the phenothiazine moieties might have played a crucial role.

We therefore set out to change the polymer backbone to poly(norbornene)s, side group‐functionalized with *N*‐methylphenothiazine (MPT), and to investigate their electrochemical performance (Figure 1). Three poly(norbornene)s with either a direct connection to the MPT units [(poly(3‐norbornyl‐*N*‐methylphenothiazine), PNMPT] or ester linkages (P1 and P2) were synthesized. Because crosslinking can be necessary to obtain complete insolubility in liquid battery electrolytes, crosslinked versions of each polymer were also synthesized [three different crosslinkers were used for PNMPT (e.g., X‐PNMPT), and two each for P1 and P2; see the Supporting Information for structures]. Out of ten linear and crosslinked polymers synthesized and investigated we found that PNMPT, in which the MPT units were directly connected to the norbornene units, as well as its crosslinked congener X‐PNMPT revealed superior electrochemical characteristics compared to the other polymers investigated herein and showed excellent performance regarding long‐term cycling stability and rate capability.

**Figure 1:**
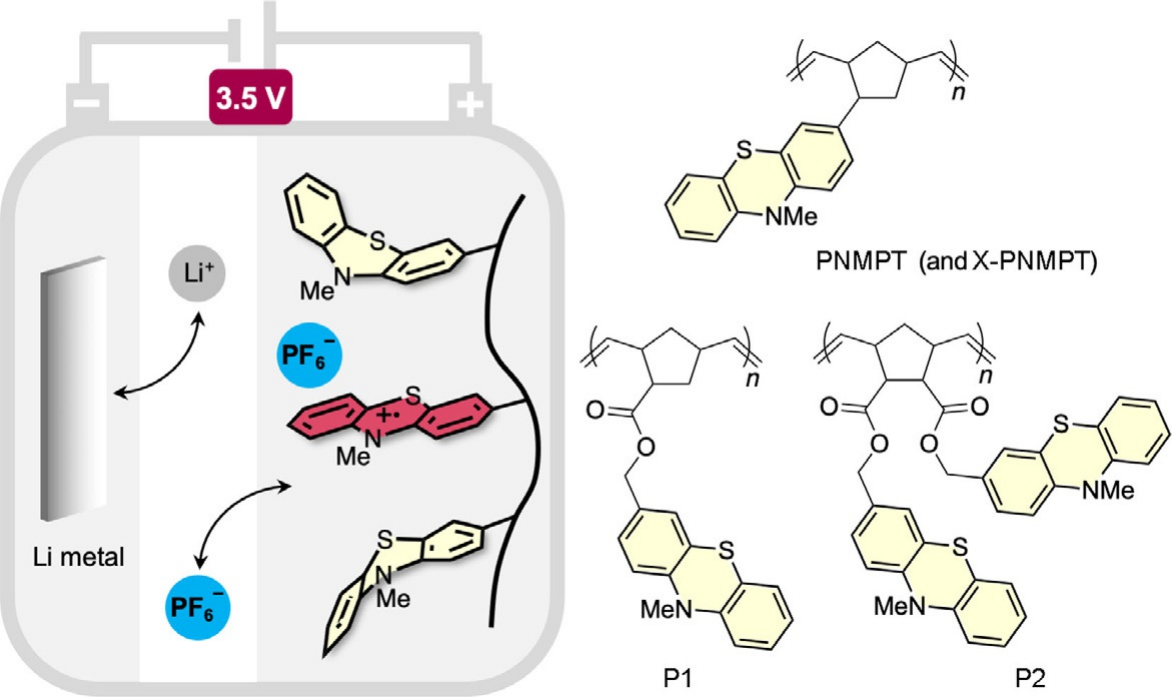
Schematic of a lithium–organic battery with a phenothiazine‐functionalized polymer as cathode‐active material and phenothiazine‐functionalized poly(norbornene)s PNMPT, P1, and P2, investigated herein.

The syntheses of directly linked poly(norbornene) PNMPT and its crosslinked derivative X‐PNMPT are shown in Scheme 1. Palladium‐catalyzed hydroarylation of norbornadiene with bromo‐MPT **1** (see the Supporting Information for the synthesis) afforded the monomer **2** in quantitative yield. The same reaction using dibromo‐MPT **3** led to the crosslinker **4** also in quantitative yield. Polymerization of **2** proceeded successfully with the Grubbs third‐generation catalyst **G3** and furnished the polymer PNMPT in excellent yield and with a high molecular weight of 1.0×10^5^ g mol^−1^. Polymerization of **2** with the addition of 10 mol % **4** as crosslinker furnished X‐PNMPT, which was insoluble in common organic solvents. Norbornadiene and 1,4‐di‐(*exo*‐norbornen‐2‐yl)benzene (**8**,^28^ for the structure see the Supporting Information) were also used as crosslinkers to synthesize X1‐PNMPT and X2‐PNMPT, respectively (see the Supporting Information for the structures).

**Scheme 1:**
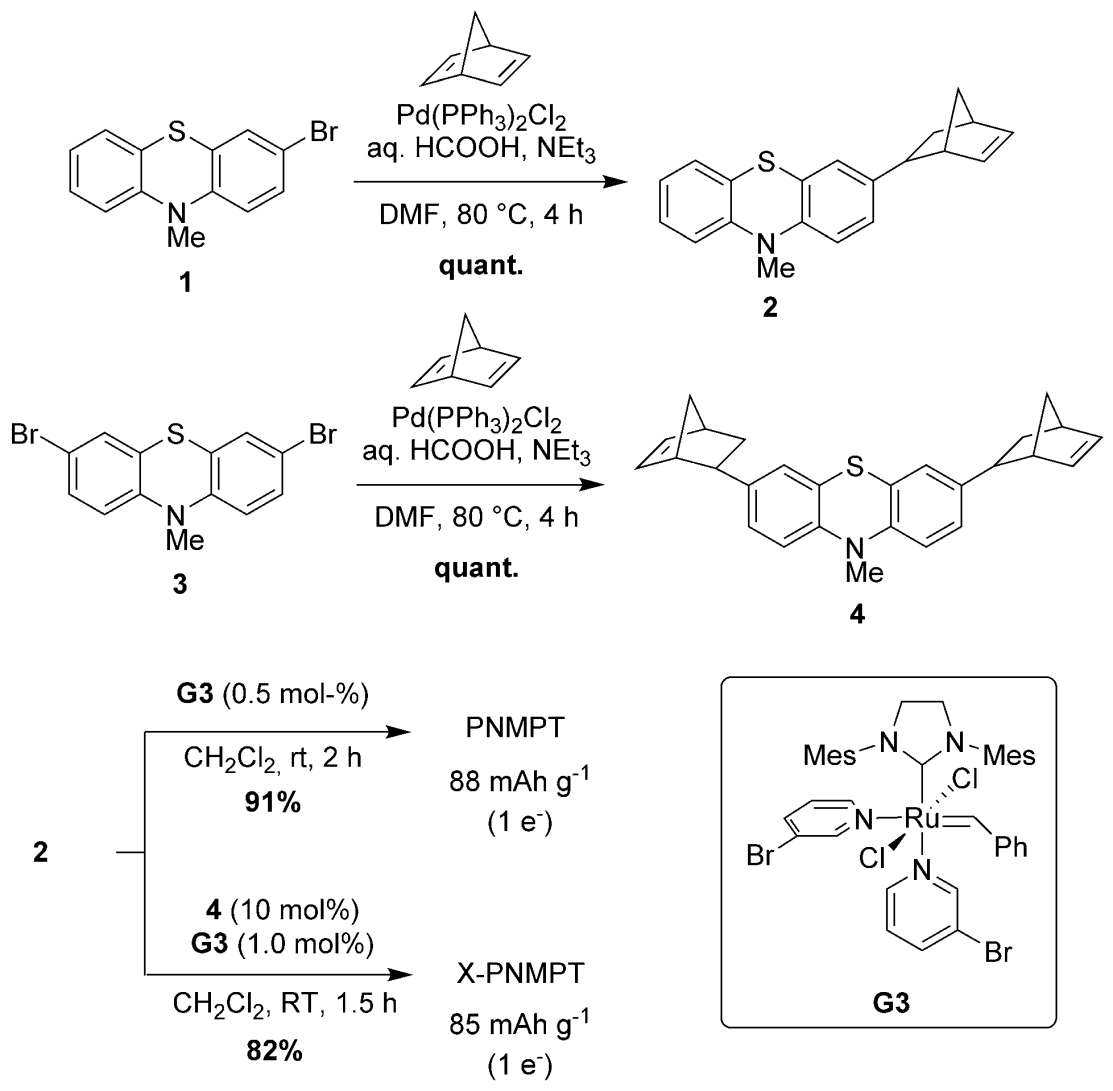
Syntheses of PNMPT and X‐PNMPT with a direct linkage between phenothiazine and the poly(norbornene) backbone.

The syntheses of the ester‐linked poly(norbornene)s P1 and P2 commenced from **5** (Scheme 2, for the synthesis of **5** see the Supporting Information). Esterification with *exo*‐5‐norbornene‐2‐carboxylic acid led to **6**, whereas **7** was synthesized by reaction with *cis*‐5‐norbornene‐*exo*‐2,3‐dicarboxylic anhydride and 2‐chloro‐1‐methylpyridinium iodide (CMPI) as the coupling reagent. Polymerization of **6** and **7** using **G3** afforded the polymers P1 and P2, respectively, in good yields and with high molecular weights. Two crosslinked versions of each P1 and P2 were also synthesized (X1‐P1, X2‐P1, X1‐P2, and X2‐P2, see the Supporting Information for details). However, they did not show better cell performance than the linear polymers (see the Supporting Information, Section 3.3 for electrochemical measurements) and will not be further discussed.

**Scheme 2:**
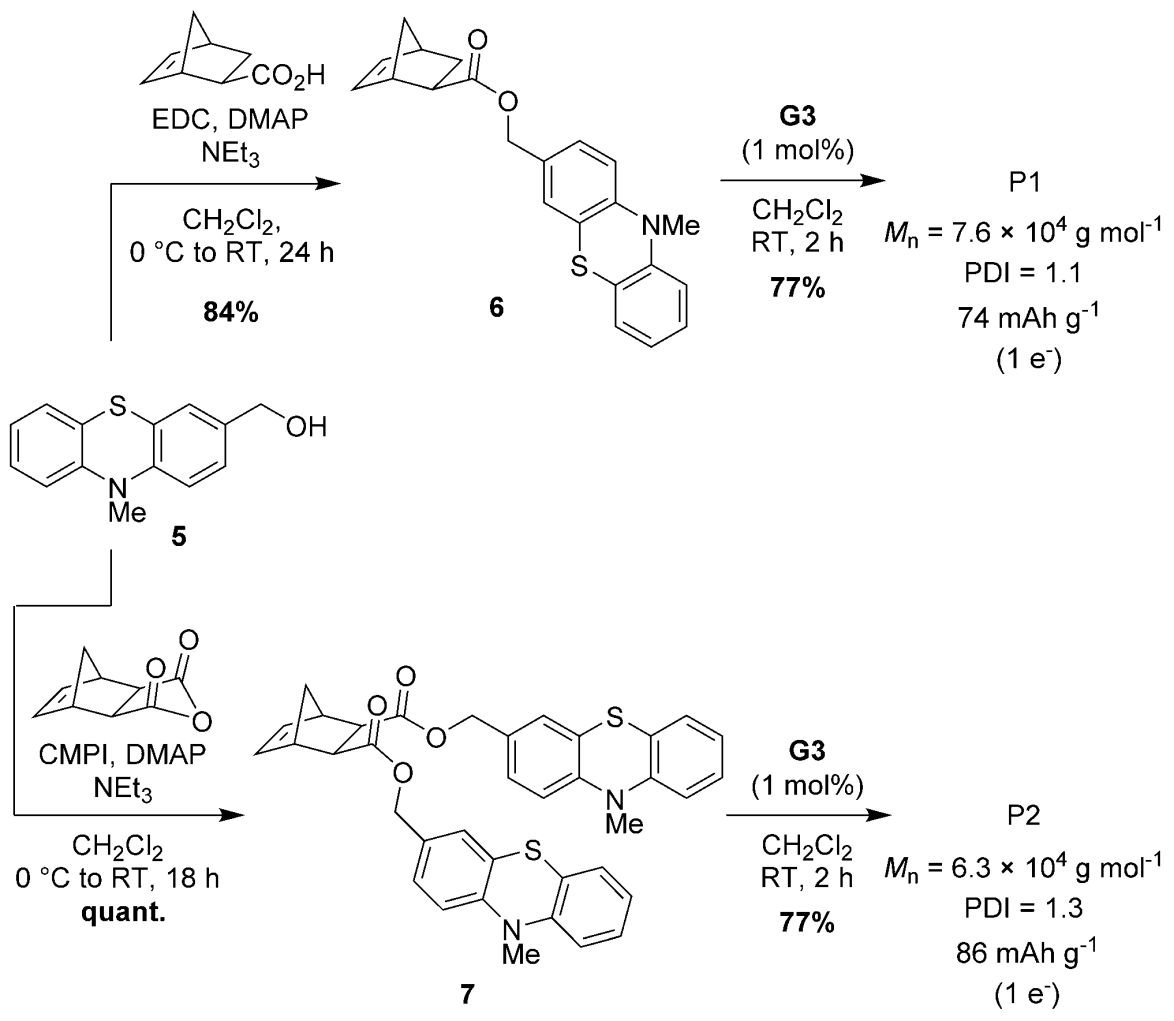
Syntheses of polymers P1 and P2 with an ester linkage between phenothiazine and the poly(norbornene) backbone.

Thermal gravimetric analyses revealed the highest thermal stabilities for PNMPT and its crosslinked forms with onsets of decomposition from 394–397 °C. For P1 and P2 and their crosslinked derivatives the onsets lay between 277 and 321 °C (details and further characterization can be found in the Supporting Information, including Figures S1–S32). Cyclic voltammetry (CV) in solution displayed reversible redox processes at 0.23 V for PNMPT, 0.29 V for P1, and 0.27 V for P2 (vs. Fc/Fc^+^) for the oxidation of each MPT side group to a radical cation (Figure 2 a–c), corresponding to potentials of 3.48, 3.54, and 3.52 V vs. Li/Li^+^, respectively (assuming 3.25 V for Fc/Fc^+^ vs. Li/Li^+^).^29^ MPT can generally be oxidized to a di‐cation; however, the second oxidation processes were less reversible for the polymers, especially for ester‐linked polymers P1 and P2 (see Figures S33–S35 in the Supporting Information).

**Figure 2:**
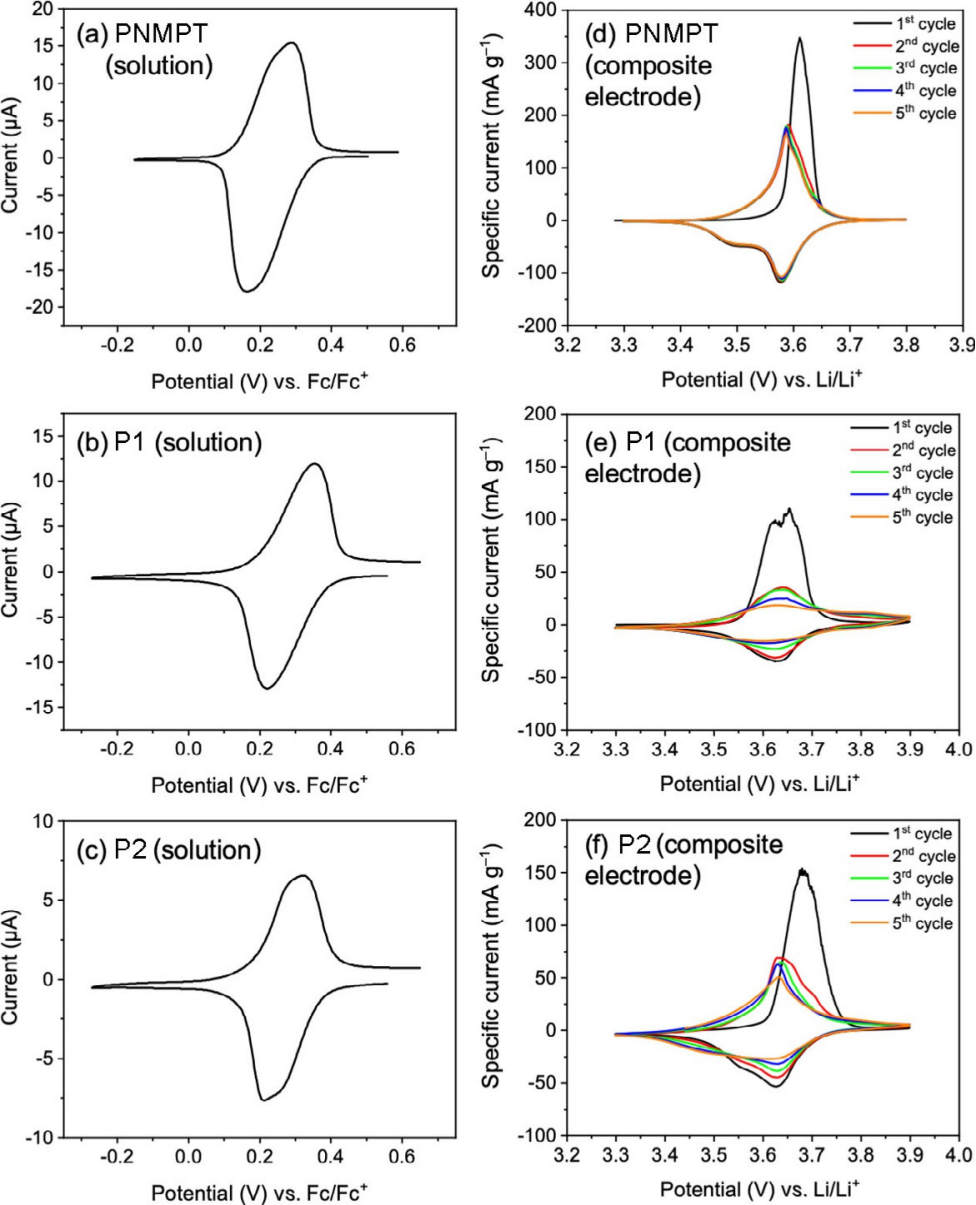
CVs of PNMPT, P1, and P2 (a–c) in solution (100 mV s^−1^, 1 mm in CH_2_Cl_2_, 0.1 m *n*Bu_4_NPF_6_, glassy carbon working electrode) and (d–f) in composite electrodes [0.05 mV s^−1^, polymer/carbon black/PVdF (50:45:5 wt %), 1 m LiPF_6_ in EC/DMC (1:1), counter/reference electrode: Li foil].

We next investigated all ten synthesized polymers as cathode‐active materials in Li–organic batteries. Composite electrodes contained 50 wt % redox‐active polymer, 45 wt % carbon black as conductive additive (Super C65), and 5 wt % polyvinylidene fluoride (PVdF) binder (the carbon additive used showed negligible specific capacity in the applied potential range, see Section 3.3.3 in the Supporting Information). We used metallic lithium as the counter and reference electrode^30^ and 1 m LiPF_6_ in ethyl carbonate/dimethyl carbonate (EC/DMC, 1:1) as electrolyte (Figure 3a). SEM measurements showed the porous, percolated network structure of the carbon black in the composite electrodes. In case of the linear polymers PNMPT, P1, and P2, this network was evenly coated with the polymer (Figure 3 a and Figures S36–S44 in the Supporting Information). For all crosslinked polymers, however, the distribution was less even owing to their insolubility in *N*‐methyl‐2‐pyrrolidone during electrode preparation. The CVs of composite electrodes showed a reversible oxidation for all polymers (Figure 2 d–f and Figures S45–S48 in the Supporting Information). For PNMPT, P1, and P2 these occurred at half‐wave potentials of 3.58, 3.63, and 3.63 V vs. Li/Li^+^ with a narrow separation of anodic and cathodic peak of 9, 15, and 6 mV, respectively, indicating diffusion‐controlled processes. The second oxidations of the phenothiazine units were observed when scanning to 4.5 V vs. Li/Li^+^ (for PNMPT see Figure S45 in the Supporting Information), but with low reversibility. Hence, only the first oxidation step was investigated in constant‐current cycling measurements.

**Figure 3:**
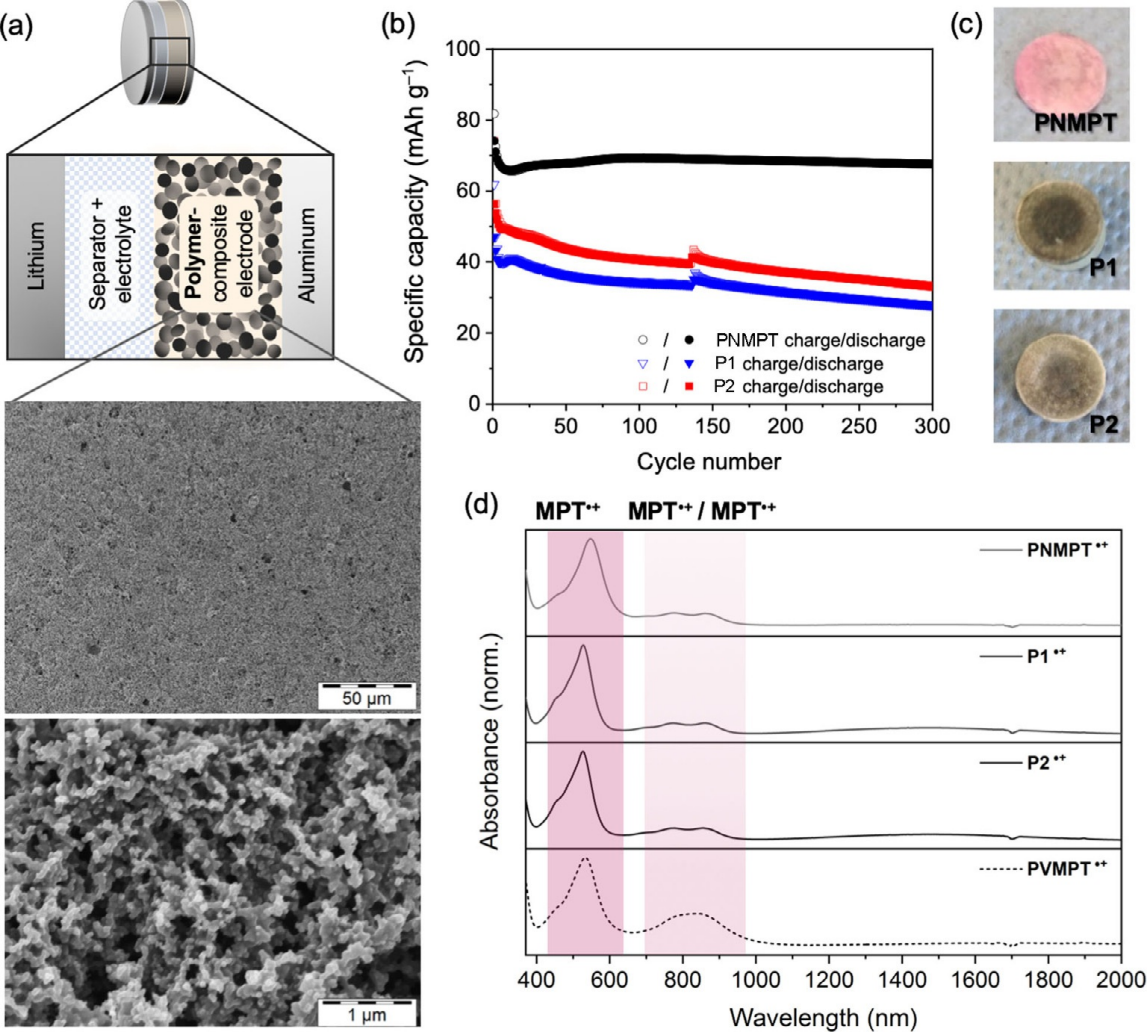
(a) Schematic of the cell setup and SEM images of a pristine PNMPT‐based composite electrode;^13^, ^22^ (b) Constant‐current cycling measurements at a 1 C rate of PNMPT‐, P1‐, and P2‐based electrodes; (c) Photographs of separators from cycled cells after 300 cycles of PNMPT, P1, and P2; (d) UV/Vis/NIR spectra of oxidized PNMPT, P1, P2 and PVMPT in CH_2_Cl_2_.

The cathodic peak in the CV of the composite electrode with PNMPT was split into two peaks (Figure 2 d). This was also observed for P2 and less strongly for P1. This might have been owing to contributions by π‐interactions between phenothiazine units and the slow scan rate we used (0.05 mV s^−1^). Similar observations have been previously reported for PVMPT.^22^ However, as we will show later, π‐interactions played a minor role in PNMPT, P1, and P2. Constant‐current cycling at 1 C rate showed a flat plateau at 3.58 V vs. Li/Li^+^ for PNMPT in the charge/discharge curves (see Figure S49 in the Supporting Information).^31^ The cycling stabilities of the three polymers were markedly different. PNMPT‐based electrodes showed stable cycling over 300 cycles with a maximum accessible specific capacity of 69 mAh g^−1^ after 90 cycles, which only faded by 3 % up to cycle 300 (Figure 3 b, for the theoretical specific capacity see Scheme 1).^32^ For ester‐linked P1 and P2, in contrast, the specific capacity retention after 300 cycles amounted to only 45 % of the initial value, corresponding to 28 and 33 mAh g^−1^, respectively (for the theoretical specific capacities see Scheme 2). We ascribe this to insufficient stability of the ester groups towards the electrolyte (and possible traces of formed HF)^33^ under battery cell operation conditions, leading—in part—to their dissolution and/or decomposition (see also the solubility tests in the Supporting Information, Figure S29). This was seen by the coloring of separators extracted from cycled cells (Figure 3 c), which were dark for P1 and P2. For PNMPT only a slight pink coloring was observed.

This degradation of the active material was also seen in differential capacity plots for P1 and P2, in which a new peak appeared at 3.8 V vs. Li/Li^+^ (see Figure S50 in the Supporting Information). The crosslinked derivatives of P1 and P2 did not show a better cycling stability (see the Supporting Information).

With 69 mAh g^−1^, the specific capacity accessible for PNMPT at 1 C rate lay at 78 % of its theoretical value of 88 mAh g^−1^. This stands in contrast to the poly(vinylene) PVMPT, for which, owing to strong π‐interactions, only half of the theoretical specific capacity was accessible.^22^ Owing to the changed polymer backbone, interactions between phenothiazine side groups became less favorable in PNMPT. This was confirmed in UV/Vis/near IR (NIR) spectra of oxidized samples of PNMPT, P1, and P2 in solution [Figure 3 d, chemically oxidized by using tris(4‐bromophenyl)ammoniumyl hexachloroantimonate]. For all three polymers the absorption bands corresponding to π‐interactions between oxidized MPT units at 700–900 nm were less expressed than the band for the non‐interacting MPT radical cations at approximately 550 nm compared to PVMPT.^12^, ^22^, ^34^ Furthermore, the broad band at approximately 1500 nm, corresponding to interactions between neutral and oxidized MPT units, showed the lowest intensity for PNMPT.

Owing to the already high cycling stability obtained for the PNMPT‐based electrodes, they were further compared to its crosslinked derivatives X‐PNMPT, X1‐PNMPT, and X2‐PNMPT. All three crosslinked polymers showed stable cycling at 1 C (see Figures S51–S53 in the Supporting Information). X‐PNMPT provided most reliable results between different cells and was probed for its rate capability in comparison with PNMPT. Constant‐current cycling at 10 C rate proceeded with high capacity retention for both polymers (Figure 4 a, b). After 1000 cycles, 61 mAh g^−1^ were retained for PNMPT and 62 mAh g^−1^ for X‐PNMPT, corresponding to 69 and 73 % of the theoretical values, respectively. The charge/discharge curves presented plateaus at potentials of 3.63 and 3.65 V vs. Li/Li^+^ during charge and 3.50 and 3.50 V vs. Li/Li^+^ during discharge for PNMPT and X‐PNMPT, respectively (Figure 4 c, d).

**Figure 4:**
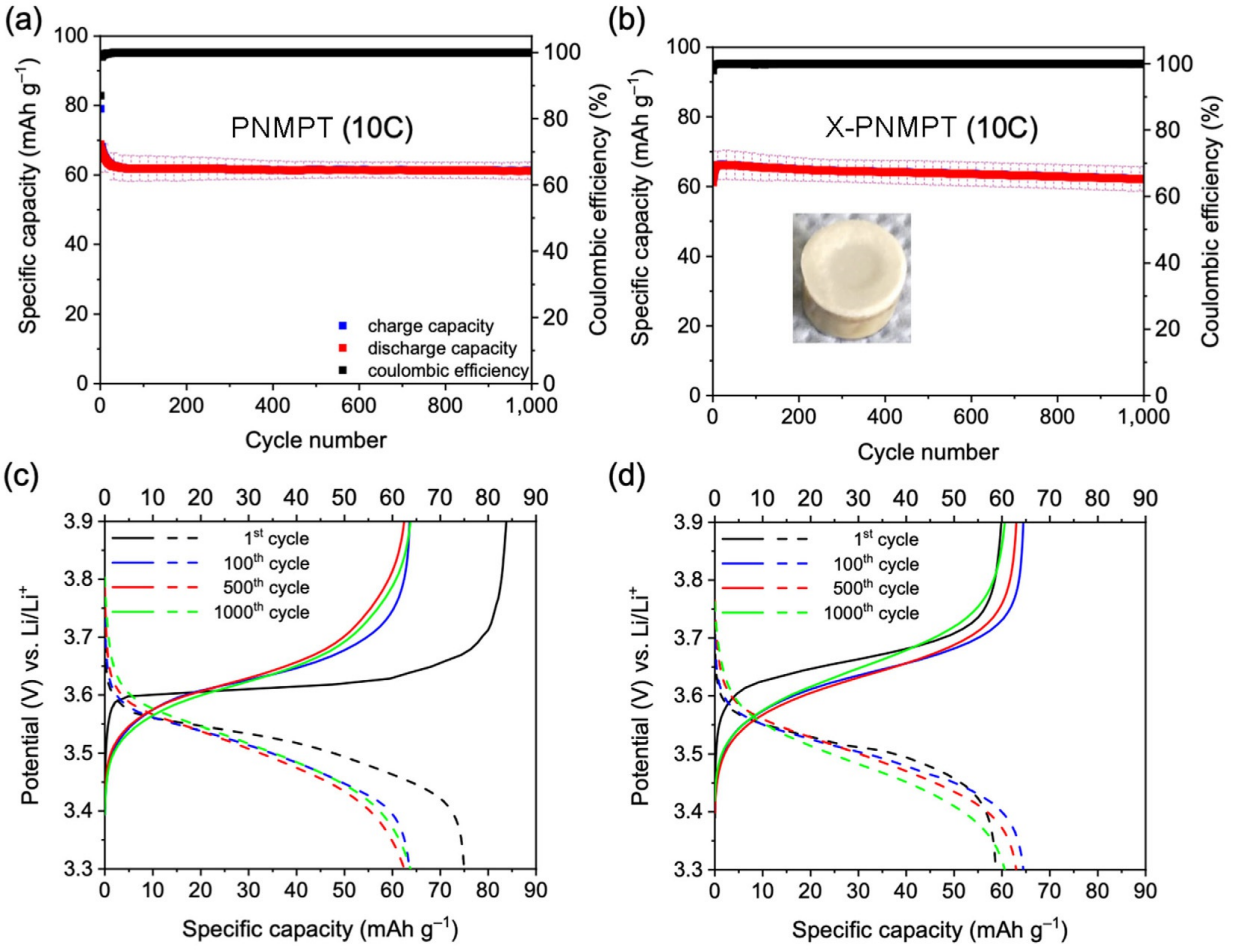
Constant‐current cycling measurements at 10 C rate of (a) PNMPT‐ and (b) X‐PNMPT‐based electrodes (average of three different cells) with selected charge/discharge curves (c, d). In (b) a photograph of a separator taken from an X‐PNMPT‐based cell after 1000 cycles is shown.

A comparison of the rate capabilities of PNMPT and X‐PNMPT demonstrated the superiority of the crosslinked polymer (Figure 5 a, b). In X‐PNMPT, C‐rates of up to 100 C were possible and provided 47 mAh g^−1^ (75 % of the initial charge capacity and 55 % of the theoretical value), whereas in the PNMPT‐based electrode the capacity significantly faded for C‐rates higher than 20 C. At 20 C rate, the specific capacity amounted to 58 mAh g^−1^ for PNMPT, showing 73 % retention of the initial capacity and 66 % of the theoretical value. A long‐term cycling experiment at the ultra‐fast rate of 100 C displayed excellent cycling stability for X‐PNMPT (Figure 5 c). A maximum specific capacity of 64 mAh g^−1^ was reached after 850 cycles, of which 73 % (47 mAh g^−1^) were retained after 10 000 cycles, corresponding to 55 % of the theoretical value.

**Figure 5:**
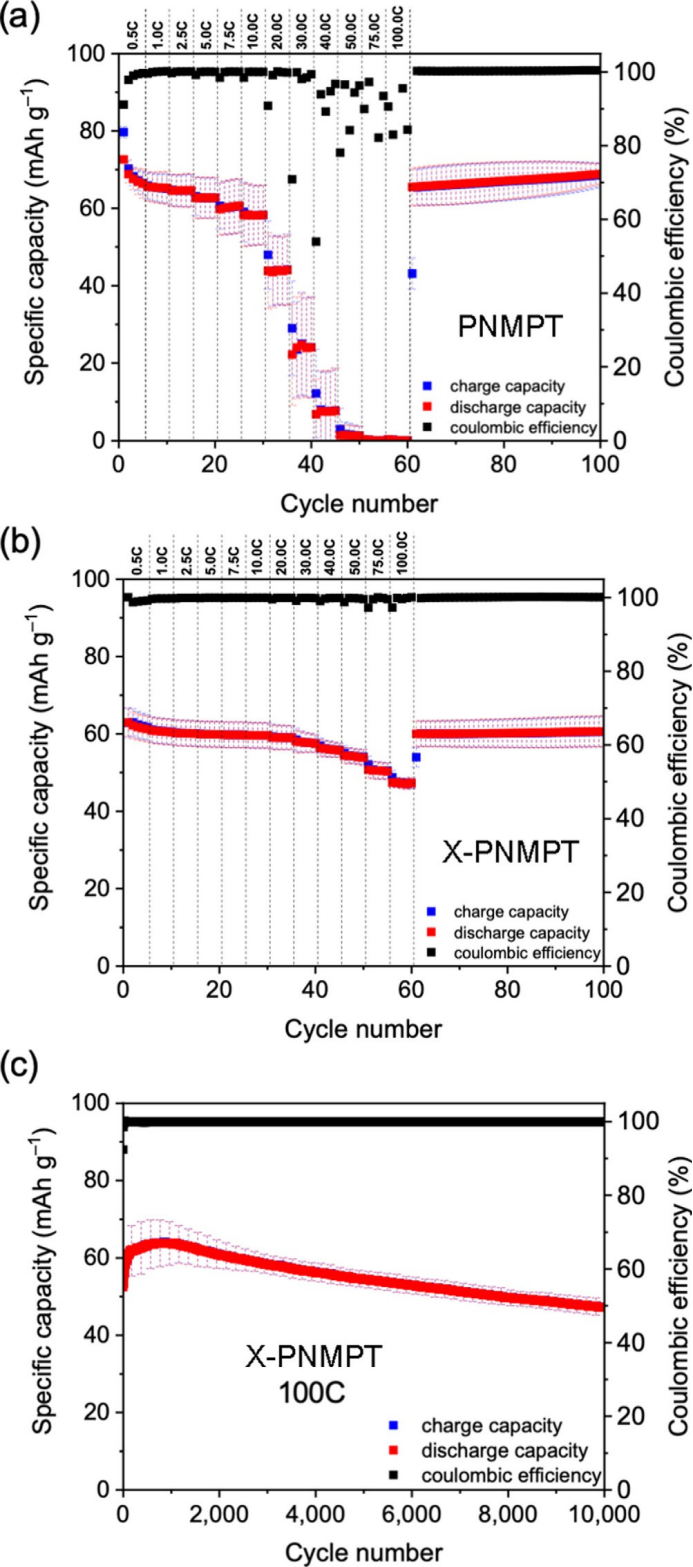
(a, b) Rate‐capability investigations in PNMPT‐ and X‐PNMPT‐based composite electrodes; (c) constant‐current cycling measurement of X‐PNMPT‐based composite electrodes at 100 C rate (average of three different cells).

This is an excellent result compared to other organic cathode materials in the literature.^23^, ^34^ Such a fast rate combined with long‐term cycling stability at a potential of 3.5 V vs. Li/Li^+^ has rarely been reported for organic cathode materials. To the best of our knowledge, long‐term cycling at 100 C has been reported only four times before: 5000 cycles were shown for a hyperbranched triphenylamine polymer with an active material loading of 40 wt % in the composite electrode,^35^ and a PTMA–graphene composite retained 100 mAh g^−1^ after 20 000 cycles, but at a low active material loading of 10 wt % in the composite electrode.^36^ The two other examples were reported by us: The conjugated phenothiazine–dithiopene copolymer P(PT‐T2) showed no capacity loss within 30 000 cycles at 100 C rate,^23^ and phenoxazine‐based X‐PVMPO provided 70 mAh g^−1^ after 10 000 cycles using an active material loading of 50 wt % in the composite electrode.^34^

These results show that the amount of π‐interactions between the redox‐active groups, the exact polymer structure, and the morphology of the composite electrode play a crucial role in the cycling behavior and rate performance of phenothiazine‐based polymers. In PNMPT and X‐PNMPT π‐interactions played a minor role, and hence specific capacities of 69 and 73 %, respectively, of the theoretical value were accessible at 10 C rate. This stands in contrast to the poly(vinylene) PVMPT, which, owing to strong π‐interactions, provided only 50 % of its theoretical specific capacity.^12^, ^22^ The ester linkages in P1 and P2 turned out to be too unstable, significantly lowering the cycling stability. Comparing linear PNMPT and crosslinked X‐PNMPT demonstrated that the network structure of the polymer in the latter was beneficial for its rate performance and provided excellent cycling stability at 100 C rate.

In summary, we presented three novel types of phenothiazine‐functionalized poly(norbornene)s with a direct (PNMPT) or ester‐linked connection (P1 and P2) to the polymer backbone in the form of ten linear and crosslinked polymers. Directly connected PNMPT and its crosslinked version X‐PNMPT provided superior cycling stability, and X‐PNMPT even demonstrated excellent rate capability and cycling stability with a capacity retention of 73 % after 10 000 cycles at 100 C rate. In comparison to other phenothiazine‐based polymers our results show that the choice of polymer backbone crucially influences π‐interactions between redox‐active groups as well as the cycling behavior and rate performance of the polymer in battery cells.

## Conflict of interest

*The authors declare no conflict of interest*.

## Supporting information

As a service to our authors and readers, this journal provides supporting information supplied by the authors. Such materials are peer reviewed and may be re‐organized for online delivery, but are not copy‐edited or typeset. Technical support issues arising from supporting information (other than missing files) should be addressed to the authors.

Supplementary
