# Supplementary material for: Phenothiazine‐Functionalized Poly(norbornene)s as High‐Rate Cathode Materials for Organic Batteries
Source: ChemSusChem. 2020 Jan 28;13(9):2232–8. doi: 10.1002/cssc.201903168 (PMC7317714; doi:10.1002/cssc.201903168)
Supplement: Supplementary file 1 — Supplementary [file CSSC-13-2232-s001.pdf]

ChemSusChem

Supporting Information

Phenothiazine-Functionalized Poly(norbornene)s as High-Rate Cathode Materials for Organic Batteries

Fabian Otteny+,[a] Gauthier Studer+,[a, b, c] Martin Kolek,*[c] Peter Bieker,[c] Martin Winter,[c, d] and Birgit Esser*[a, b, e]

Supplementary information for

Phenothiazine-functionalized poly(norbornene)s as high-rate cathode-materials for organic batteries

Fabian Otteny,[a],+ Gauthier Studer,[a],[b],[c],+ Martin Kolek,*[c] Peter Bieker,[c] Martin Winter,[c],[d] and Birgit Esser*[a],[b],[e]

[a] Institute for Organic Chemistry, University of Freiburg, Albertstraße 21, 79104 Freiburg, Germany

[b] Freiburg Materials Research Center, University of Freiburg, Stefan-Meier-Straße 21, 79104 Freiburg, Germany

[c] MEET Battery Research Center, Institute of Physical Chemistry, University of Münster, Corrensstraße 46, 48149 Münster, Germany

[d] Helmholtz Institute Münster (HI MS), IEK-12, Forschungszentrum Jülich GmbH, Corrensstrasse 46, 48149 Münster, Germany

[e] Cluster of Excellence livMatS @ FIT – Freiburg Center for Interactive Materials and Bioinspired Technologies, University of Freiburg, Georges-Köhler-Allee 105, 79110 Freiburg, Germany

[+] These authors contributed equally to this work.

* Correspondence: besser@oc.uni-freiburg.de, martin.kolek@uni-muenster.de

Contents

1 Synthesis and characterization data 3
1.1 Materials and methods 3
1.2 Synthesis of *N*-methylphenothiazine functionalized poly(norbornene)s 5
1.2.1 Synthesis of monomers 5
1.2.2 Synthesis of linear polymers 9
1.2.3 Synthesis of cross-linker 11
1.2.4 Synthesis of cross-linked polymers 13
1.3 Characterization data 17
1.3.1 NMR spectra 17
1.3.2 Stereochemical determination of norbornyl-*N*-methylphenothiazine **2** 26
1.3.3 FT-IR spectra of **MPT** poly(norbornene)s 30
1.3.4 Thermal gravimetric analyses (TGA) of **MPT** poly(norbornene)s 32
1.3.5 Differential scanning calorimetry (DSC) measurements of **MPT** poly(norbornene)s 33
1.3.6 Solubility tests of **PNMPT**, **P1** and **P2** 35
2 Spectroscopic and electrochemical investigations 36
2.1 UV/Vis/NIR spectroscopy 36
2.2 Cyclic voltammetry in solution 37
3 Investigations on poly(norbornene)-based composite electrodes 39
3.1 Fabrication of composite electrodes 39
3.2 SEM investigation of pristine **MPT** poly(norbornene)-based electrodes 39
3.3 Electrochemical analyses 42
3.3.1 Cyclic voltammetry investigations of **MPT** poly(norbornene)-based electrodes 43
3.3.2 Constant current cycling and rate capability tests of **MPT** poly(norbornene)-based electrodes 45
3.3.3 Constant current cycling of a *Super C65*-based electrode for comparison 50
4 References 51

1 Synthesis and characterization data

1.1 Materials and methods

Commercially available chemicals were purchased from ABCR, ACROS, ALFA AESAR, CHEMPUR, FISHER CHEMICAL, SIGMA-ALDRICH and TCI and used without further purification unless otherwise noted. Solvents purchased in technical grade were distilled prior to use, analytical grade solvents were used as received. Anhydrous solvents were obtained from an M. BRAUN solvent purification system (*MB-SPS-800*) and stored over molecular sieves (3 Å) for a minimum duration of 72 h. Air- and moisture-sensitive reactions were carried out under an argon atmosphere in glassware dried by heating under vacuum using standard Schlenk techniques (Argon 5.0 from SAUERSTOFFWERK FRIEDRICHSHAFEN).

Analytical thin layer chromatography was carried out by using silica gel-coated aluminium plates with a fluorescence indicator (MERCK *60 F_{254}*). Detection was carried out by using short-wave UV light (λ_{max} = 254 nm). Flash column chromatography was carried out by using silica gel *Silica 60* (grain size 40–63 µm, 230–400 mesh) from MACHERY-NAGEL.

NMR spectra were recorded on BRUKER *Avance III HD* (^{1}H = 500 MHz, ^{13}C = 125 MHz) and BRUKER *Avance II* (^{1}H = 400 MHz, ^{13}C = 100 MHz) spectrometers in deuterated solvent solution at 298 K. Chemical shifts are reported in parts per million (ppm, δ scale) and are referenced to the residual solvent signal ($CDCl_3$: δ_H 7.26 ppm, δ_C = 77.16 ppm). Analysis followed first order and data are presented as follows: chemical shift, multiplicity (s = singlet, br. s = broad singlet, d = doublet, dd = doublet of doublet, ddd = doublet of doublet of doublet, m = multiplet), coupling constants (*J*) in Hertz [Hz] and integration.

HRMS spectra were measured on a THERMO FISHER SCIENTIFIC *Exactive* spectrometer with orbitrap analyzer or on an AGILENT *6500 Series Q-TOF* instrument. For ionization APCI and ESI were used.

FT-IR measurements were performed on a THERMO FISHER SCIENTIFIC *Nicolet iS10* spectrometer equipped with a diamond-ATR module. Spectra were recorded at 298 K in the range of 4000–550 cm^{-1} with a resolution of 2 cm^{-1} and averaged over 64 scans. The THERMO FISHER SCIENTIFIC software *OMNIC* was used for acquiring data. All spectra were normalized to 1 and intensities are given as follows: vw = very weak (< 0.2), w = weak (< 0.4), m = medium (< 0.6), s = strong (< 0.8), vs = very strong (≥ 0.8).

UV/Vis/NIR absorption spectra were measured with a *Lambda 950* spectrometer from PERKINELMER using sealable *Quarz Suprasil* cuvettes from HELLMA ANALYTICS.

Elemental analysis was performed on an ELEMENTAR *vario MICRO cube* using a thermal conductivity detector (TCD).

GPC was performed on a *SECcurity GPC System* from PSS POLYMER STANDARDS SERVICE using components of the *1260 Infinity* series from AGILENT TECHNOLOGIES. Measurements in tetrahydrofurane (THF) were performed at 35 °C with a flow rate of 1 mL min^{-1} and a set of three columns (PSS SDV, 8 mm × 50 mm

pre-column, 8 mm × 300 mm columns with a porosity of 1.000 Å and 100.000 Å). For calibration, polystyrene standards by PSS were used. Measurements in *N*,*N*-dimethylacetamide (DMAc; containing 0.5 wt% LiBr) were performed at 50 °C with a flow rate of 1 mL min^{-1} and a set of 4 columns (PSS GRAM with 10 µm particle size, 8 mm × 50 mm pre-column, 8 mm × 300 mm columns with a porosity of 30 Å, 1.000 Å and 1.000 Å). For calibration, poly(methyl methacrylate) (PMMA) standards by PSS were used.

TGA (thermogravimetric analysis) was performed on a NETZSCH *STA 409 C* or *STA 449 F5 Jupiter* and **DSC** (differential scanning calorimetry) on a NETZSCH *DSC 204 F1 Phoenix*. Evaluation of the data was perfomed with *Proteus Thermal Analysis* by NETZSCH.

Cyclic voltammograms (CVs) were measured at ambient temperature inside an argon filled glovebox employing a potentiostat *PGSTAT128N* by METROHM AUTOLAB using a three-electrode-setup. As working electrode (WE) a glassy carbon disc electrode (2 mm diameter) was used, as counter electrode (CE) a platinum rod and as reference electrode (RE) a Ag/$AgNO_3$ electrode containing a silver wire immersed in an inner chamber filled with $AgNO_3$ (0.1 M) and *n*-Bu_4NPF_6 (0.1 M) in anhydrous acetonitrile. Measurements were performed in anhydrous solvent (1 mM of analyte, referenced to the amount of redox-active subunits in case of a polymer) containing *n*-Bu_4NPF_6 (0.1 M) as supporting electrolyte. The redox couple ferrocene/ferrocenium (Fc/Fc^+) was used as internal reference.

1.2 Synthesis of *N*-methylphenothiazine functionalized poly(norbornene)s

1.2.1 Synthesis of monomers

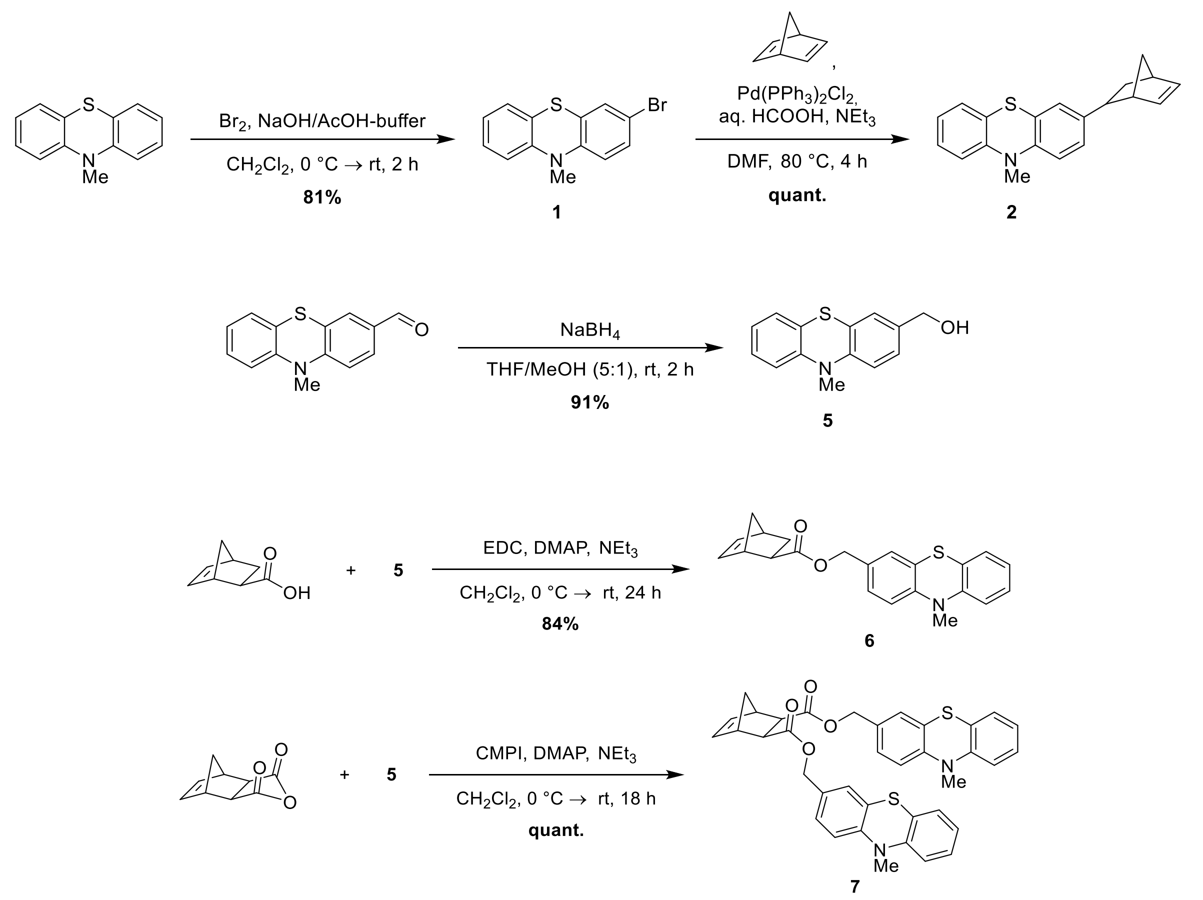

N-Methylphenothiazine and *N*-methylphenothiazinyl-3-carbaldehyde were synthesized according to a previously published procedure.[1,2]

Synthesis of 3-bromo-*N*-methylphenothiazine (1)

1 was prepared following a literature procedure.[3] A solution of *N*-methylphenothiazine (8.53 g, 40.0 mmol) in dichloromethane (80 mL) was added to a sodium acetate buffer, prepared by dissolving sodium hydroxide (4.88 g) in acetic acid (300 mL). After cooling to 0 °C a solution of bromine (2.15 mL, 42.0 mmol) in acetic acid (40 mL) was added within 2 h. The reaction mixture was allowed to warm to room temperature and was stirred for an additional 2 h. The reaction was stopped by removing the solvent under reduced pressure. The pink residue was dissolved in dichloromethane (100 mL) and washed with a saturated aqueous solution of $NaHCO_3$ (100 mL). The aqueous layer was extracted with dichloromethane (3 × 100 mL), the combined organic layers were dried over $MgSO_4$, filtered and evaporated to dryness under reduced pressure. The crude product was pre-purified via filtration

through a silica pad and elution with cyclohexane/ethyl acetate and then further purified via column chromatography (silica gel, cyclohexane/ethyl acetate/triethylamine: 10/0.5/0.2) to afford 3-bromo-*N*-methylphenothiazine (**1**, 9.52 g, 32.6 mmol, 81%) as a colorless crystalline solid. R_f 0.51 (cyclohexane/ethyl acetate: 9/1); ^{1}H NMR (400 MHz, $CDCl_3$): δ 7.26–7.23 (m, 2H), 7.18 (ddd, *J* = 8.1, 7.4, 1.6 Hz, 1H), 7.12 (dd, 7.6, 1.5 Hz, 1H), 6.95 (ddd, 7.5, 7.5, 1.2 Hz, 1H), 6.80 (dd, *J* = 8.1, 1.2 Hz, 1H), 6.63 (d, *J* = 9.2 Hz, 1H), 3.33 (s, 3H); ^{13}C NMR (100 MHz, $CDCl_3$): δ 145.5, 145.1, 130.1, 129.4, 127.8, 127.3, 125.8, 122.9, 122.8, 115.3, 114.8, 114.3, 35.5; HR-MS (ESI+): *m/z* calcd. for $C_{13}H_{10}BrNS$ 290.9712 $[M]^+$, found 290.9714 $[M]^+$.

Synthesis of 3-norbornyl-*N*-methylphenothiazine (2)

A solution of freshly distilled norbornadiene (1.46 mL, 14.4 mmol), aqueous formic acid (90%, v/v, 0.36 mL, 9.5 mmol) and triethylamine (1.46 mL, 10.5 mmol) in *N*,*N*-dimethylformamide (1.9 mL) was saturated with argon for 15 min. 3-Bromo-*N*-methylphenothiazine (**1**, 1.02 g, 3.50 mmol) and bis(triphenylphosphine)palladium dichloride (100 mg, 0.14 mmol) were added and the reaction mixture was stirred under argon atmosphere for 4 hours at 80 °C. After cooling to room temperature, the mixture was diluted with toluene (10 mL) and washed with aqueous hydrochloric acid (2.8 M, 20 mL). The aqueous layer was extracted with toluene (3 × 50 mL), the combined organic layers were dried over $MgSO_4$, filtered and evaporated to dryness under reduced pressure. Column chromatography (silica gel, cyclohexane/ethyl acetate: 30/1) afforded 3-norbornyl-*N*-methylphenothiazine (**2**, 1.07 g, 3.50 mmol, quant.) as a colorless oil that turned solid after several days. **2** was obtained as a mixture of two diastereomeres (*exo*/*endo*) in a ratio of 91/9. For ring opening metathesis polymerizations this mixture was used without prior separation. Unless otherwise noted characterization refers to the *exo*-form as the main diastereomer. R_f (*exo*) 0.66 and R_f (*endo*) 0.68 (cyclohexane/ethyl acetate: 19/1); ^{1}H NMR (500 MHz, $CDCl_3$): δ 7.18–7.14 (m, 2H), 7.08–7.06 (m, 2H), 6.92 (ddd, *J* = 7.5, 7.5, 1.2 Hz, 1H), 6.80 (dd, *J* = 8.0, 0.9 Hz, 1H), 6.75 (d, *J* = 9.0 Hz, 1H), 6.23 (dd, *J* = 5.7, 3.1 Hz, 1H), 6.15 (dd, *J* = 5.7, 2.9 Hz, 1H), 3.36 (s, 3H), 2.95 (br. s, 1H), 2.85–2.84 (m, 1H), 2.62 (dd, *J* = 8.7, 4.7 Hz, 1H), 1.68 (ddd, *J* = 11.8, 4.7, 3.5 Hz, 1H), 1.60 (ddd, *J* = 11.4, 8.8, 2.4 Hz, 1H), 1.54–1.53 (m, 1H), 1.43–1.40 (m, 1H); ^{13}C NMR (125 MHz, $CDCl_3$): δ 146.1, 143.6, 140.6, 137.5, 137.3, 127.5, 127.3, 126.6, 126.4, 123.4, 123.3, 122.3, 114.0, 113.9, 48.5, 45.9, 43.0, 42.4, 35.4, 33.7; HR-MS (ESI+): *m/z* calcd. for $C_{20}H_{20}NS$ 306.1311 $[M+H]^+$, found 306.1312 $[M+H]^+$.

Synthesis of 3-hydroxymethyl-*N*-methylphenothiazine (5)

5 was prepared following a literature procedure.[4] To a solution of *N*-methylphenothiazinyl-3-carbaldehyde (4.67 g, 19.4 mmol) in a mixture of tetrahydrofuran (10 mL) and methanol (2 mL) was

added $NaBH_4$ (1.13 g, 29.9 mmol) in three portions within 20 min. The reaction mixture was stirred for 2 h at room temperature. The solvent was removed under reduced pressure, the residue was diluted with ethyl acetate (100 mL), washed with brine (50 mL) and extracted with ethyl acetate (3 × 50 mL). The combined organic layers were dried over $MgSO_4$, filtered and evaporated to dryness under reduced pressure. Column chromatography (silica gel, cyclohexane/ethyl acetate: 3/1 to 1/1) afforded 3-hydroxymethyl-*N*-methylphenothiazine (**5**, 4.28 g, 17.6 mmol, 91%) as a colorless solid. R_f 0.32 (cyclohexane/ethyl acetate: 2/1); ^{1}H NMR (500 MHz, $CDCl_3$): δ 7.19–7.13 (m, 4H), 6.93 (dd, *J* = 7.5, 7.5 Hz, 1H), 6.81 (d, *J* = 8.1 Hz, 1H), 6.78 (d, *J* = 8.7 Hz, 1H), 4.57 (s, 2H), 3.37 (s, 3H), 1.64 (br. s, 1H); ^{13}C NMR (125 MHz, $CDCl_3$): δ 145.8, 145.5, 135.2, 127.6, 127.3, 126.5, 126.2, 123.8, 123.3, 122.6, 114.2, 114.1, 64.8, 35.5; HR-MS (ESI+): *m/z* calcd. for $C_{14}H_{14}NOS$ 244.0791 $[M+H]^+$, found 244.0791 $[M+H]^+$.

Synthesis of *exo*-5-norbornene-2-carboxylic acid (*N*-methylphenothiazin-3-yl)methyl ester (6)

To a mixture of *exo*-5-norbornene-2-carboxylic acid[1] (536 mg, 3.88 mmol) and 3-hydroxymethyl-*N*-methylphenothiazine (**5**, 1.89 g, 7.75 mmol) in dry dichloromethane (5.5 mL) was added 1-ethyl-3-(3-dimethylaminopropyl)carbodiimide (EDC, 1.00 mL, 5.70 mmol) at 0 °C, followed by the addition of *N*,*N*-dimethylpyridin-4-amine (DMAP, 95 mg, 0.78 mmol) and triethylamine (NEt_3, 0.35 mL, 2.5 mmol). The suspension was allowed to warm to room temperature and stirred for 24 h. The yellow solution was diluted with dichloromethane (20 mL), washed with aqueous hydrochloric acid (1 vol-%, 20 mL), and extracted with dichloromethane (4 × 50 mL). The combined organic layers were dried over $MgSO_4$, filtered and the solvent was removed under reduced pressure. Column chromatography (silica gel, cyclohexane/ethyl acetate: 3/1) afforded *exo*-5-norbornene-2-carboxylic acid (*N*-methylphenothiazin-3-yl)methyl ester (**6**, 1.18 g, 3.25 mmol, 84%) as a colorless oil. R_f 0.53 (cyclohexane/ethyl acetate: 5/1); ^{1}H NMR (500 MHz, $CDCl_3$): δ 7.19–7.15 (m, 3H), 7.14 (dd, *J* = 7.6, 1.5 Hz, 1H), 6.93 (ddd, *J* = 7.5, 7.5, 1.2 Hz, 1H), 6.81 (dd, *J* = 8.1, 1.1 Hz, 1H), 6.78 (d, *J* = 8.5 Hz, 1H), 6.13 (dd, *J* = 5.6, 3.0 Hz, 1H), 6.09 (dd, *J* = 5.6, 3.1 Hz, 1H), 5.01 (s, 2H), 3.37 (s, 3H), 3.05–3.04 (m, 1H), 2.91 (br. s, 1H), 2.24 (dd, *J* = 10.2, 4.6 Hz, 1H), 1.92 (ddd, *J* = 11.8, 4.0, 4.0 Hz, 1H), 1.53–1.51 (m, 1H), 1.39–1.34 (m, 2H); ^{13}C NMR (125 MHz, $CDCl_3$): δ 176.2, 146.0, 145.7, 138.2, 135.9, 130.6, 127.9, 127.7, 127.4, 127.3, 123.8, 123.2, 122.7, 114.3, 114.1, 65.8, 46.8, 46.5, 43.3, 41.8, 35.5, 30.5; HR-MS (ESI+): *m/z* calcd. for $C_{22}H_{22}NO_2S$ 364.1366 $[M+H]^+$, found 364.1365 $[M+H]^+$.

---

[1] Pure *exo*-5-norbornene-2-carboxylic acid was obtained from commercially available racemic *exo*/*endo* mixture after applying a literature procedure, where the availability of the *endo* diastereomere to undergo iodolactonization was used for segregation.[6]

Synthesis of *cis*-*exo*-5-norbornene-2,3-dicarboxylic acid bis(*N*-methylphenothiazin-3-yl)methyl ester (7)

To a mixture of *cis*-*exo*-5-norbornene-2,3-dicarboxylic anhydride (164 mg, 1.00 mmol) and 3-hydroxymethyl-*N*-methylphenothiazine (**5**, 608 mg, 2.50 mmol) in dry dichloromethane (8 mL) was added 2-chloro-1-methylpyridinium iodide (CMPI, 307 mg, 1.20 mmol) at 0 °C, followed by the addition of *N*,*N*-dimethylpyridin-4-amine (DMAP, 49 mg, 0.40 mmol) and triethylamine (NEt_3, 0.41 mL, 2.9 mmol). The suspension was allowed to warm to room temperature and stirred for 18 h. Dichloromethane (20 mL) was added, the mixture was washed with aqueous hydrochloric acid (1 vol-%, 20 mL) and extracted with dichloromethane (3 × 20 mL). The combined organic layers were dried over $MgSO_4$, filtered and the solvent was removed under reduced pressure. Column chromatography (silica gel, cyclohexane/ethyl acetate: 4/1) afforded *cis*-*exo*-5-norbornene-2,3-dicarboxylic acid bis(*N*-methylphenothiazin-3-yl)methyl ester (**7**, 631 mg, 1.00 mmol, quant.) as a colorless solid. R_f 0.58 (cyclohexane/ethyl acetate: 2/1); ^{1}H NMR (500 MHz, $CDCl_3$): δ 7.14 (ddd, *J* = 8.1, 7.5, 1.5 Hz, 2H), 7.10 (dd, *J* = 7.6, 1.5 Hz, 2H), 7.04–7.02 (m, 4H), 6.91 (ddd, *J* = 7.5, 7.5, 1.1 Hz, 2H), 6.76 (dd, *J* = 8.1, 0.9 Hz, 2H), 6.67 (d, *J* = 8.1 Hz, 2H), 6.18 (dd, *J* = 1.9, 1.9 Hz, 2H), 4.91 (d, *J* = 12.1 Hz, 2H), 4.82 (d, *J* = 12.1 Hz, 2H), 3.30 (s, 6H), 3.10–3.08 (m, 2H), 2.62 (d, *J* = 1.8 Hz, 2H), 2.17–2.14 (m, 1H), 1.52–1.48 (m, 1H); ^{13}C NMR (125 MHz, $CDCl_3$): δ 173.5, 145.9, 145.6, 138.1, 130.0, 128.1, 127.6, 127.5, 127.2, 123.6, 123.2, 122.7, 114.2, 114.0, 66.1, 47.4, 45.8, 45.5, 35.4; HR-MS (APCI−): *m/z* calcd. for $C_{37}H_{32}ClN_2O_4S_2$ 667.1497 $[M+Cl]^-$, found 667.1495 $[M+Cl]^-$.

1.2.2 Synthesis of linear polymers

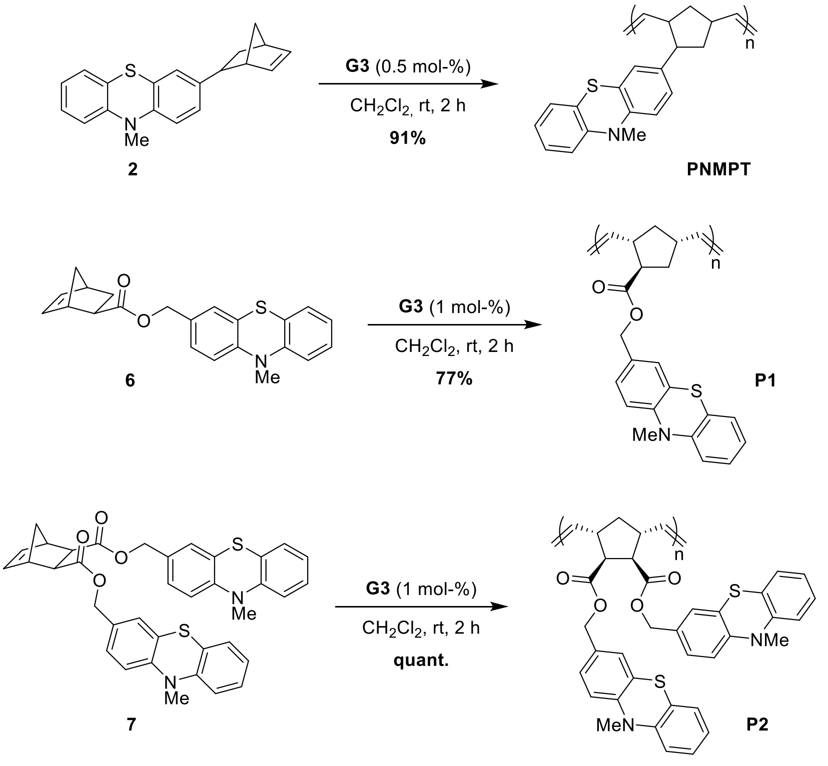

General procedure (exact conditions see Table S1)

To a rigorously stirred solution of the respective monomer in dichloromethane was added a solution of the 3rd generation Grubbs catalyst (**G3**, dichloro[1,3-bis(2,4,6-trimethylphenyl)-2-imidazolidinyl-idene](benzylidene)bis(3-bromopyridine)ruthenium(II)) in dichloromethane and the mixture was stirred for 2 h at room temperature. The reaction was stopped by the addition of ethyl vinyl ether (5 mL), and the solvent was removed under reduced pressure. The residue was dissolved in dichloromethane, and the polymer was precipitated from cold acetone and cyclohexane for purification.

Table S1. Polymerization conditions and results for the linear polymers **PNMPT**, **P1** and **P2**.

Polymer	Monomer	m_{mon} (mg)	n_{mon} (mmol)	$V_{LM, mon}$ (mL)	m_{G3} (mg)	n_{G3} (µmol)	$V_{LM, G3}$ (mL)	yield (%)	M_n [a] (g mol^{-1})	PDI [a]
**PNMPT**	**2**	560	1.83	10	8.1	9.2	2.5	91	$1 \cdot 10^5$	6.9
**P1**	**6**	315	0.87	3.4	7.7	8.7	2.4	77	$6 \cdot 10^4$	1.4
**P2**	**7**	323	0.51	2.0	4.5	5.1	1.4	quant.	-	-

[a] Due to the solubility properties of the polymers GPC systems based on different eluents had to be used. Depicted values for **PNMPT** refer to THF-GPC, **P1** to DMAc-GPC. **P2** was not completely soluble in appropriate solvents, but showed modes in the range from 10^5 to 10^8 g mol^{-1} indicating broad weight distribution and high polymerization degrees.

Poly(3-norbornyl-*N*-methylphenothiazine) (PNMPT)

^{1}H NMR (400 MHz, $CDCl_3$): δ 7.19–6.38 (m, 7H), 5.48–4.76 (m, 2H), 3.45–2.95 (m, 3H), 2.91–2.18 (m, 3H), 2.16–1.62 (m, 2H), 1.39–0.64 (m, 2H); FT-IR (ATR): $\tilde{\nu}_{max}$ = 744 (vs), 809 (m), 1038 (w), 1109 (m), 1141 (m), 1260 (m), 1332 (s), 1464 (vs), 2938 (w) cm^{-1}; UV/Vis (CH_2Cl_2): λ_{max} (log ε) = 313 nm (3.81); elemental analysis: calcd (%) for $C_{20}H_{19}NS$: C 78.65, H 6.27, N 4.59, S 10.50; found: C 78.56, H 6.42, N 4.14, S 10.77; GPC (eluent THF, polystyrene standard): M_n 1.0 × 10^5, M_w/M_n 6.9; TGA (10 °C min^{-1}, N_2): onset 397 °C, T_d10% (temperature for 10% weight loss) 401 °C; DSC (10 °C min^{-1}, air): T_g 149 °C.

Poly(*exo*-5-norbornene-2-carboxylic acid (*N*-methylphenothiazin-3-yl)methyl ester) (P1)

^{1}H NMR (500 MHz, $CDCl_3$): 7.18–6.96 (m, 4H), 6.93–6.80 (m, 1H), 6.78–6.53 (m, 2H), 5.39–5.05 (m, 2H), 5.01–4.82 (m, 2H), 3.36–3.14 (m, 3H), 3.12–2.31 (m, 3H), 2.17–1.71 (m, 2H), 1.68–1.57 (m, 1H), 1.19–0.92 (m, 1H); FT-IR (ATR): $\tilde{\nu}_{max}$ = 747 (vs), 810 (s), 966 (m), 1049 (m), 1108 (m), 1140 (vs), 1160 (s), 1259 (s), 1332 (s), 1464 (vs), 1725 (vs), 2850 (m), 2925 (m) cm^{-1}; UV/Vis (CH_2Cl_2): λ_{max} (log ε) = 313 nm (3.66); elemental analysis: calcd (%) for $C_{22}H_{21}NO_2S$: C 72.70, H 5.82, N 3.85, S 8.82; found: C 72.79, H 6.51, N 3.22, S 8.31; GPC (eluent DMAc, polystyrene standard): M_n 6.1 × 10^4, M_w/M_n 1.4; TGA (10 °C min^{-1}, N_2): onset 314 °C, T_d10% (temperature for 10% weight loss) 84 °C (due to solvent loss, decomposition at ca. 310 °C); DSC (10 °C min^{-1}, air): T_g 96 °C.

Poly(*cis*-*exo*-5-norbornene-2,3-dicarboxylic acid bis(*N*-methylphenothiazin-3-yl)methyl ester) (P2)

^{1}H NMR (500 MHz, $CDCl_3$): 7.15–6.75 (m, 10H), 6.68–6.37 (m, 4H), 5.40–4.96 (m, 2H), 4.93–4.52 (m, 4H), 3.56–2.39 (m, 10H), 2.37–1.70 (m, 1H), 1.21–0.91 (m, 1H); FT-IR (ATR): $\tilde{\nu}_{max}$ = 747 (vs), 810 (s), 1038 (m), 1109 (s), 1140 (vs), 1164 (s), 1260 (s), 1332 (s), 1464 (vs), 1733 (s), 2851 (m), 2925 (m) cm^{-1}; UV/Vis (CH_2Cl_2): λ_{max} (log ε) = 314 nm (3.74); elemental analysis: calcd (%) for $C_{37}H_{32}N_2O_4S_2$: C 70.23, H 5.10, N 4.43, S 10.13; found: C 69.78, H 6.05, N 3.61, S 9.53; TGA (10 °C min^{-1}, N_2): onset 277 °C, T_d10% (temperature for 10% weight loss) 115 °C (due to solvent loss, decomposition at ca. 270 °C); DSC (10 °C min^{-1}, air): T_g 107 °C.

1.2.3 Synthesis of cross-linker

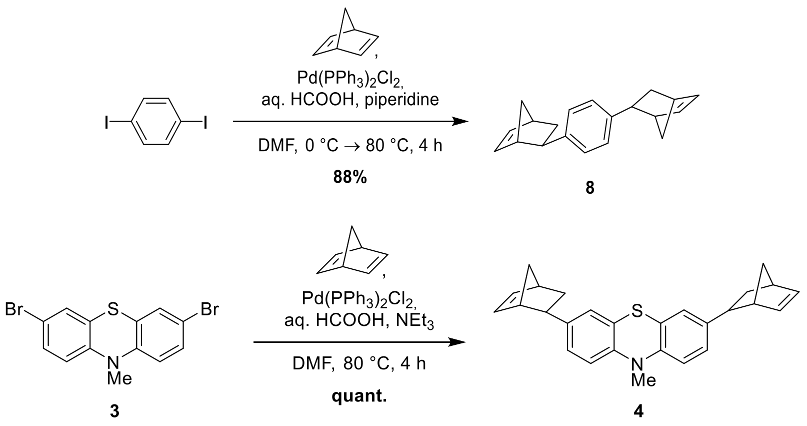

3,7-Dibromo-*N*-methylphenothiazine (**3**) was synthesized from *N*-methylphenothiazine according to a previously published procedure.[2]

Synthesis of 1,4-di-(*exo*-norbornen-2-yl)benzene (8)

8 was prepared following a modified literature procedure.[5] A degassed mixture of freshly distilled norbornadiene (4.88 mL, 48.0 mmol) and piperidine (2.18 mL, 22.0 mmol) in *N*,*N*-dimethylformamide (5 mL) was added to 1,4-diiodobenzene (1.32 g, 4.00 mmol) and bis(triphenylphosphine)palladium dichloride (0.22 g, 0.32 mmol). The mixture was stirred at 0 °C and degassed formic acid (0.83 mL, 22.0 mmol) was slowly added. After allowing to warm up to room temperature within 3 h, the mixture was heated to 60 °C and stirred for an additional 4 h. After cooling to room temperature, water (20 mL) was added and the mixture was extracted with diethyl ether (3 × 30 mL). The combined organic layers were dried over $MgSO_4$, filtered and evaporated to dryness under reduced pressure. Column chromatography (silica gel, cyclohexane) yielded 1,4-di-(*exo*-norbornen-2-yl)benzene (**8**, 920 mg, 3.51 mmol, 88%) as a white solid. R_f 0.43 (cyclohexane); ^{1}H NMR (500 MHz, $CDCl_3$): δ 7.23–7.22 (m, 4H), 6.27–6.25 (m, 2H), 6.18–6.16 (m, 2H), 2.97 (br. s, 2H), 2.90 (br. s, 2H), 2.72–2.69 (m, 2H), 1.78–1.73 (m, 2H), 1.65–1.59 (m, 4H), 1.44–1.42 (m, 2H); ^{13}C NMR (125 MHz, $CDCl_3$): δ 143.4, 137.5, 137.4, 127.6, 48.5, 45.9, 43.4, 42.4, 33.7; HR-MS (APCI+): *m/z* calcd. for $C_{20}H_{23}$ 263.1794 $[M+H]^+$, found 263.1796 $[M+H]^+$.

Synthesis of 3,7-dinorbornyl-*N*-methylphenothiazine (4)

Aqueous formic acid (90%, v/v, 50.0 µL, 1.35 mmol) and triethylamine (230 µL, 1.62 mmol) in *N*,*N*-dimethylformamide (0.4 mL) were saturated with argon for 15 min and added to a mixture of 3,7-dibromo-*N*-methylphenothiazine (**3**, 100 mg, 269 µmol) and bis(triphenylphosphine)palladium

dichloride (15 mg, 22 µmol). Freshly distilled norbornadiene (220 µL, 2.15 mmol) was added, the mixture further saturated with argon for 10 min and then stirred under argon atmosphere for 4 h at 80 °C. After cooling to room temperature, the mixture was diluted with toluene (2 mL), quenched with water (1 mL), and washed with aqueous hydrochloric acid (2.9 M, 10 mL). The aqueous layer was extracted with toluene (3 × 20 mL), the combined organic layers were dried over $MgSO_4$, filtered and evaporated to dryness under reduced pressure. Column chromatography (silica gel, cyclohexane/ethyl acetate: 30/1) afforded 3,7-dinorbornyl-*N*-methylphenothiazine (**4**, 107 mg, 269 µmol, quant.) as a colorless oil that turned solid after several days. **4** was obtained as a mixture of diastereomers (*exo*/*endo*) in a ratio of 91/9. For ring opening metathesis polymerizations this mixture was used without prior separation. Unless otherwise noted characterization refers to the *exo*,*exo*-form as the main diastereomer. R_f (*exo*,*exo*) 0.66 and R_f (*exo*,*endo*) 0.70 (cyclohexane/ethyl acetate: 19/1); ^{1}H NMR (500 MHz, $CDCl_3$): δ 7.07–7.05 (m, 4H), 6.73 (d, *J* = 8.1 Hz, 2H), 6.23 (dd, *J* = 5.6, 3.1 Hz, 2H), 6.14 (dd, *J* = 5.7, 2.9 Hz, 2H), 3.34 (s, 3H), 2.94 (br. s, 2H), 2.84–2.82 (m, 2H), 2.61 (dd, *J* = 8.6, 4.7 Hz, 2H), 1.66 (ddd, *J* = 11.9, 4.4, 3.8 Hz, 2H), 1.59 (ddd, *J* = 11.5, 8.8, 2.3 Hz, 2H), 1.52 (d, *J* = 8.6 Hz, 2H), 1.42–1.39 (m, 2H); ^{13}C NMR (125 MHz, $CDCl_3$): δ 143.8, 140.3, 137.5, 137.3, 126.6, 126.4, 123.2, 113.8, 48.5, 45.9, 43.0, 42.4, 35.3, 33.7; HR-MS (APCI+): *m/z* calcd. for $C_{27}H_{28}NS$ 398.1937 $[M+H]^+$, found 398.1939 $[M+H]^+$.

1.2.4 Synthesis of cross-linked polymers

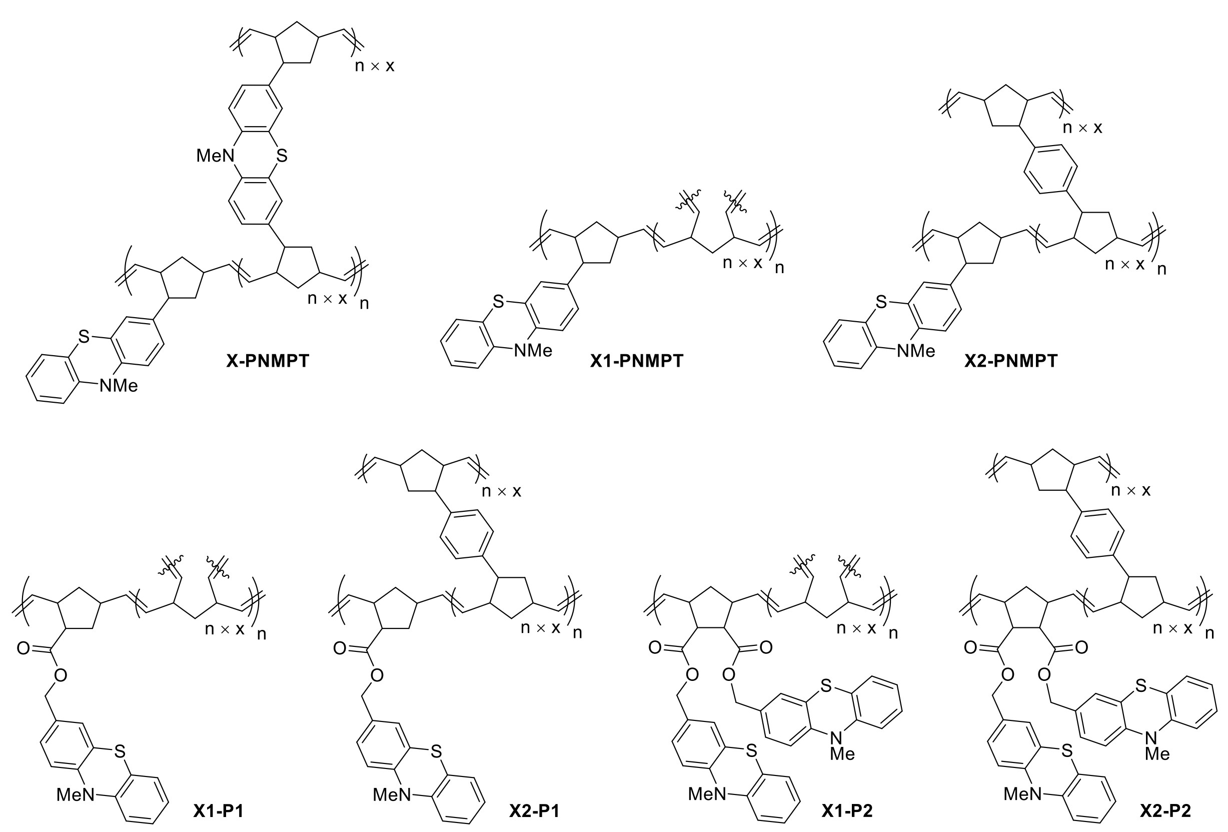

General procedure (exact conditions see Table S2-Table S4)

The cross-linked polymers shown above were synthesized by ring opening metathesis polymerization in analogy to their linear derivatives (see chapter 1.2.2), but in the presence of the cross-linker 3,7-dinorbornyl-*N*-methylphenothiazine (**4**), norbornadiene (norb.) or 1,4-di-(*exo*-norbornen-2-yl)benzene (**8**). The coefficient x displayed in the cross-linked polymer structures is based on the theoretical ratio of cross-linker inside the polymer ($x = n_X * (n_X + n_{mon})^{-1}$) and represents the degree of cross-linking. As a consequence, n × x is the statistical chain length between two branches with n as the number of repetitive units.

To a rigorously stirred solution of monomer and cross-linker in dichloromethane was added a solution of **G3** in dichloromethane and the mixture was stirred at room temperature for 1.5 h in the case of **PNMPT**-based polymers and for 2 h in the case of **P1**- and **P2**-based polymers. The reaction was stopped by addition of ethyl vinyl ether (5 mL), and the solvent was removed under reduced pressure.

The crude product was pestled into a powder,[2] suspended with dichloromethane, allowed to swell until the phase looked homogeneous,[3] and precipitated from cold acetone and cyclohexane for purification.

Table S2. Polymerization conditions and results for the cross-linked polymers based on **PNMPT**.

Polymer	X-linker	m_{mon} (mg)	n_{mon} (mmol)	m_X (mg)	n_X (µmol)	$V_{LM, mon+X}$ (mL)	m_{G3} (mg)	n_{G3} (µmol)	$V_{LM, G3}$ (mL)	yield (%)
**X-PNMPT**	**4**	340	1.11	44	111	4.7	10.6	12	2.8	82
**X1-PNMPT**	norb.	410	1.34	6.2	67	5.5	11.5	13	3.0	67
**X2-PNMPT**	**8**	442	1.45	19	73	5.9	12.8	15	3.4	81

Table S3. Polymerization conditions and results for the cross-linked polymers based on **P1**.

Polymer	X-linker	m_{mon} (mg)	n_{mon} (mmol)	m_X (mg)	n_X (µmol)	$V_{LM, mon+X}$ (mL)	m_{G3} (mg)	n_{G3} (µmol)	$V_{LM, G3}$ (mL)	yield (%)
**X1-P1**	norb.	301	0.83	3.8	42	3.7	7.3	8.3	2.3	quant.
**X2-P1**	**8**	213	0.59	7.7	29	2.4	5.5	6.2	1.7	87

Table S4. Polymerization conditions and results for the cross-linked polymers based on **P2**.

Polymer	X-linker	m_{mon} (mg)	n_{mon} (mmol)	m_X (mg)	n_X (µmol)	$V_{LM, mon+X}$ (mL)	m_{G3} (mg)	n_{G3} (µmol)	$V_{LM, G3}$ (mL)	yield (%)
**X1-P2**	norb.	313	0.49	2.3	25	2.5	4.3	4.9	1.3	quant.
**X2-P2**	**8**	193	0.31	4.0	15	1.2	2.8	3.2	0.9	87

Poly(3-norbornyl-*N*-methylphenothiazine-*co*-3,7-dinorbornyl-*N*-methylphenothiazine) (X-PNMPT)

FT-IR (ATR): $\tilde{\nu}_{max}$ = 744 (vs), 809 (m), 1109 (w), 1141 (m), 1259 (m), 1332 (s), 1464 (vs), 2864 (w), 2935 (w) cm^{-1}; elemental analysis: calcd (%) for $C_{20}H_{19}NS$: C 78.65, H 6.27, N 4.59, S 10.50; found: C 79.88, H 6.78, N 3.78, S 9.86; TGA (10 °C min^{-1}, N_2): onset 395 °C, T_d10% (temperature for 10% weight loss) 398 °C; DSC (10 °C min^{-1}, air): no T_g detectable.

[2] For some polymers, especially those containing cross-linker **8**, pestling was not possible due to their gum-like texture. In those cases, the swollen polymers were shredded in dichloromethane with a spatula to enlarge the surface and enhance diffusion through the polymeric particles. Furthermore, longer swelling times were applied.

[3] Polymers obtained using norbornadiene as cross-linker showed limited solubility in dichloromethane. This might be taken as evidence for a lower degree of cross-linkage.

Poly(3-norbornyl-*N*-methylphenothiazine-*co*-norbornadiene) (X1-PNMPT)

FT-IR (ATR): $\tilde{\nu}_{max}$ = 744 (vs), 809 (m), 1038 (w), 1109 (w), 1141 (m), 1259 (m), 1332 (m), 1464 (vs), 2864 (w), 2938 (w) cm^{-1}; elemental analysis: calcd (%) for $C_{20}H_{19}NS$: C 78.65, H 6.27, N 4.59, S 10.50; found: C 79.65, H 6.63, N 3.85, S 9.81; TGA (10 °C min^{-1}, N_2): onset 394 °C, T_d10% (temperature for 10% weight loss) 402 °C; DSC (10 °C min^{-1}, air): T_g 132 °C.

Poly(3-norbornyl-*N*-methylphenothiazine-*co*-dinorbornylbenzene) (X2-PNMPT)

FT-IR (ATR): $\tilde{\nu}_{max}$ = 572 (m), 741 (vs), 808 (s), 965 (m), 1038 (m), 1109 (m), 1140 (s), 1259 (m), 1332 (s), 1463 (vs), 2863 (w), 2935 (m) cm^{-1}; elemental analysis: calcd (%) for $C_{20}H_{19}NS$: C 78.65, H 6.27, N 4.59, S 10.50; found: C 79.84, H 6.52, N 3.89, S 9.55; TGA (10 °C min^{-1}, N_2): onset 396 °C, T_d10% (temperature for 10% weight loss) 401 °C; DSC (10 °C min^{-1}, air): T_g 132 °C.

Poly(*exo*-5-norbornene-2-carboxylic acid (*N*-methylphenothiazin-3-yl)methyl ester-*co*-norbornadiene) (X1-P1)

FT-IR (ATR): $\tilde{\nu}_{max}$ = 748 (s), 811 (s), 966 (m), 1049 (s), 1108 (s), 1140 (vs), 1161 (vs), 1261 (s), 1332 (s), 1464 (vs), 1726 (vs), 2849 (s), 2923 (s) cm^{-1}; elemental analysis: calcd (%) for $C_{22}H_{21}NO_2S$: C 72.70, H 5.82, N 3.85, S 8.82; found: C 69.20, H 6.32, N 3.10, S 7.93; TGA (10 °C min^{-1}, N_2): onset 305 °C, T_d10% (temperature for 10% weight loss) 90 °C (due to solvent loss, decomposition at ca. 310 °C); DSC (10 °C min^{-1}, air): T_g 96 °C.

Poly(*exo*-5-norbornene-2-carboxylic acid (*N*-methylphenothiazin-3-yl)methyl ester-*co*-dinorbornylbenzene) (X2-P1)

FT-IR (ATR): $\tilde{\nu}_{max}$ = 746 (vs), 809 (s), 965 (m), 1049 (m), 1108 (s), 1140 (vs), 1160 (s), 1258 (s), 1332 (s), 1464 (vs), 1725 (vs), 2849 (m), 2924 (m) cm^{-1}; elemental analysis: calcd (%) for $C_{22}H_{21}NO_2S$: C 72.70, H 5.82, N 3.85, S 8.82; found: C 67.10, H 5.79, N 3.04, S 7.58; TGA (10 °C min^{-1}, N_2): onset 321 °C, T_d10% (temperature for 10% weight loss) 110 °C (due to solvent loss, decomposition at ca. 310 °C); DSC (10 °C min^{-1}, air): T_g 98 °C.

Poly(*cis*-*exo*-5-norbornene-2,3-dicarboxylic acid bis(*N*-methylphenothiazin-3-yl)methyl ester-*co*-norbornadiene) (X1-P2)

FT-IR (ATR): $\tilde{\nu}_{max}$ = 747 (s), 810 (s), 966 (m), 1039 (m), 1109 (m), 1140 (s), 1175 (s), 1259 (s), 1332 (s), 1464 (vs), 1734 (s), 2850 (w), 2924 (w) cm^{-1}; elemental analysis: calcd (%) for $C_{37}H_{32}N_2O_4S_2$: C 70.23, H 5.10, N 4.43, S 10.13; found: C 71.00, H 6.03, N 3.69, S 9.85; TGA (10 °C min^{-1}, N_2): onset 288 °C, T_d10%

(temperature for 10% weight loss) 116 °C (due to solvent loss, decomposition at ca. 280 °C); DSC (10 °C min^{-1}, air): T_g 99 °C.

Poly(*cis*-*exo*-5-norbornene-2,3-dicarboxylic acid bis(*N*-methylphenothiazin-3-yl)methyl ester-*co*-dinorbornylbenzene) (X2-P2)

FT-IR (ATR): $\tilde{\nu}_{max}$ = 747 (vs), 810 (s), 1038 (m), 1108 (s), 1140 (vs), 1164 (s), 1259 (s), 1332 (s), 1464 (vs), 1731 (s), 2881 (w), 2953 (w) cm^{-1}; elemental analysis: calcd (%) for $C_{37}H_{32}N_2O_4S_2$: C 70.23, H 5.10, N 4.43, S 10.13; found: C 70.23, H 5.33, N 4.07, S 10.23; TGA (10 °C min^{-1}, N_2): onset 292 °C, T_d10% (temperature for 10% weight loss) 321 °C; DSC (10 °C min^{-1}, air): T_g 125 °C.

1.3 Characterization data

1.3.1 NMR spectra

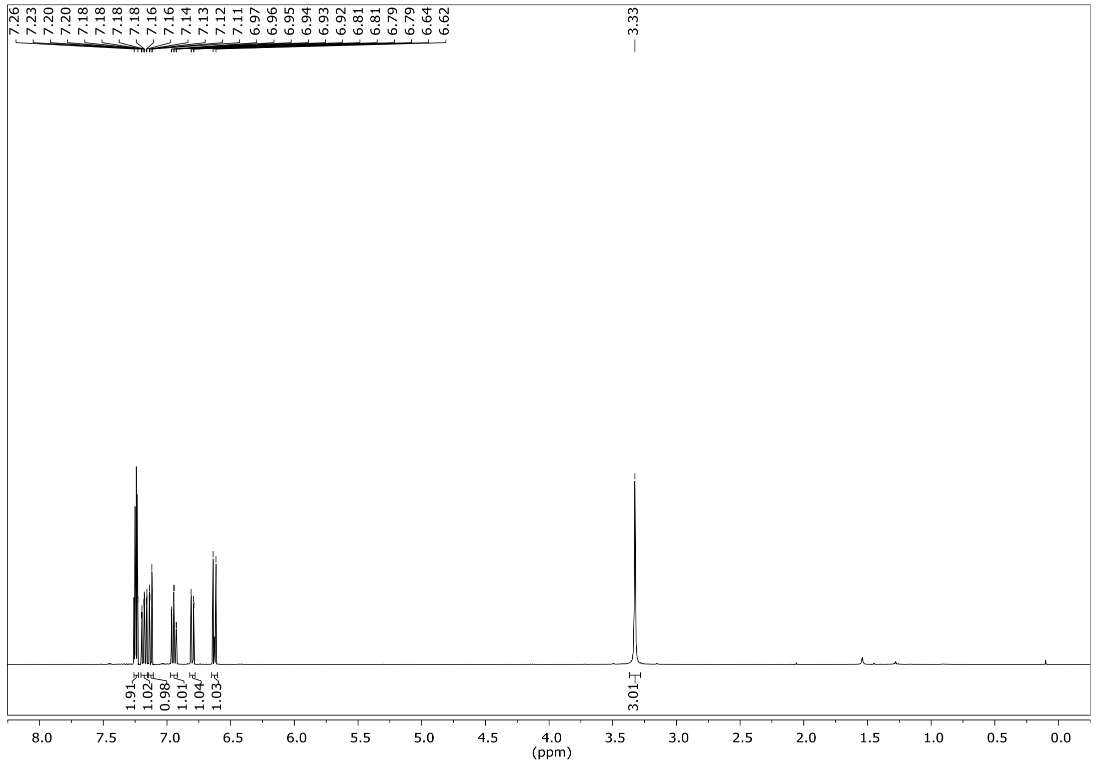

Figure S1. 400 MHz ^{1}H NMR spectrum of 3-bromo-*N*-methylphenothiazine (**1**) in $CDCl_3$.

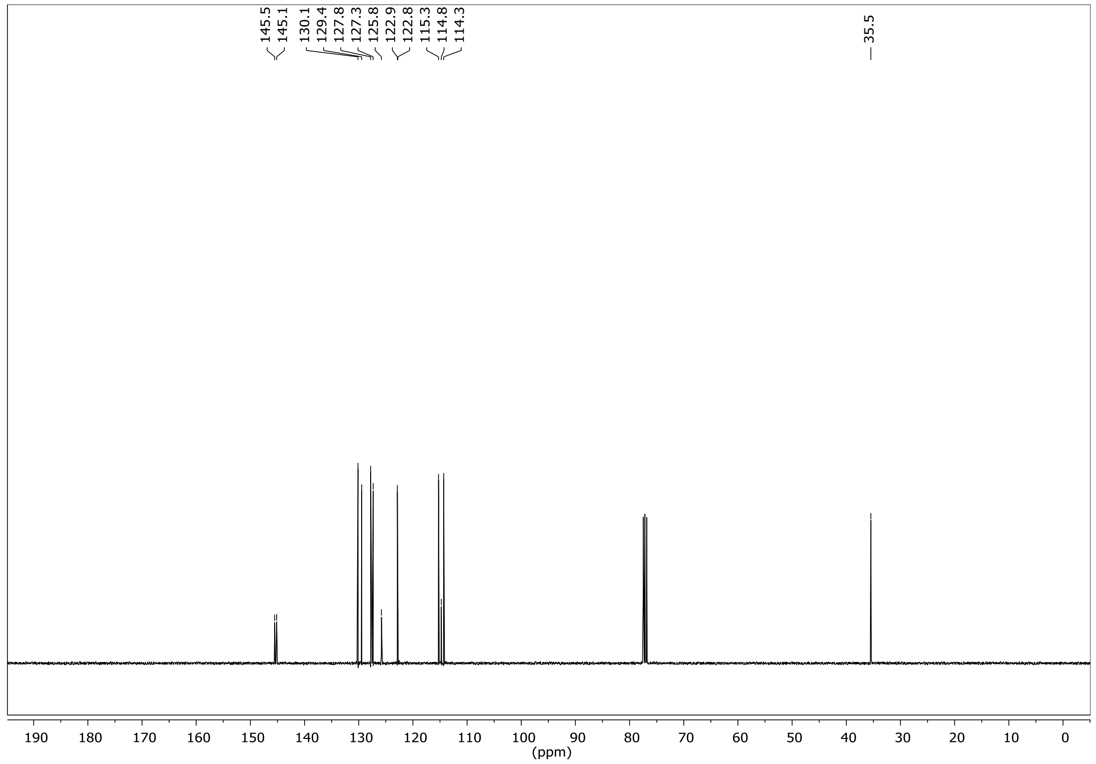

Figure S2. 100 MHz ^{13}C NMR spectrum of 3-bromo-*N*-methylphenothiazine (**1**) in $CDCl_3$.

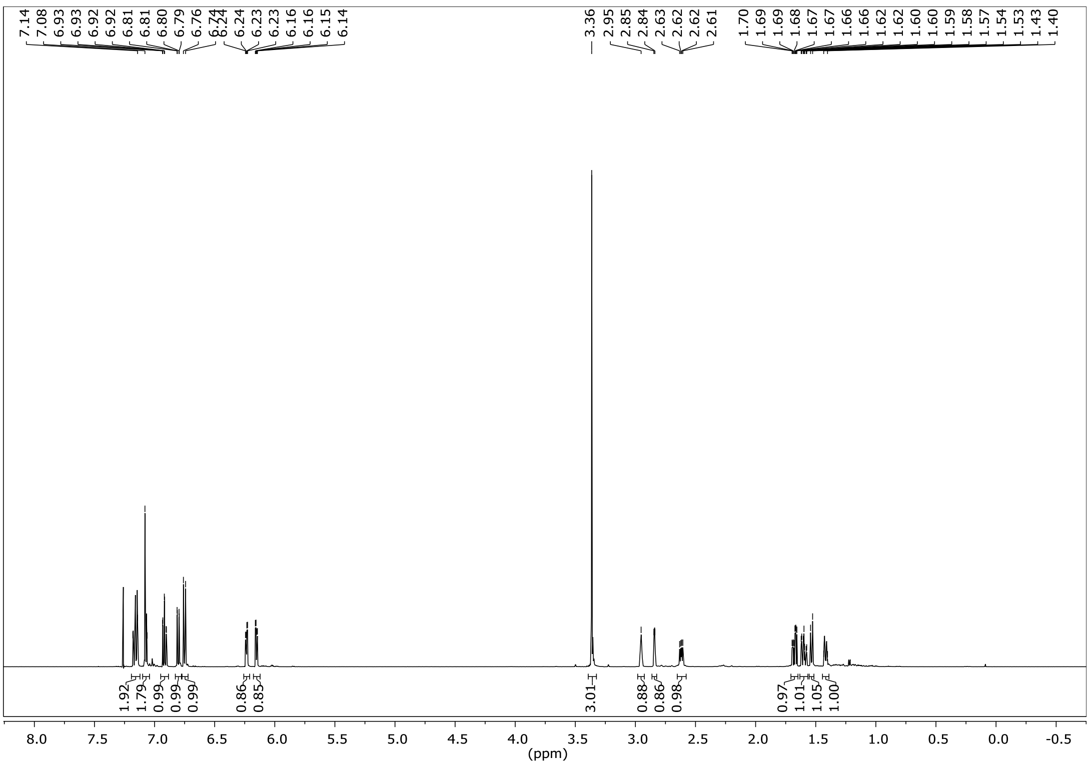

Figure S3. 500 MHz ^{1}H NMR spectrum of 3-norbornyl-*N*-methylphenothiazine (**2**) in $CDCl_3$.

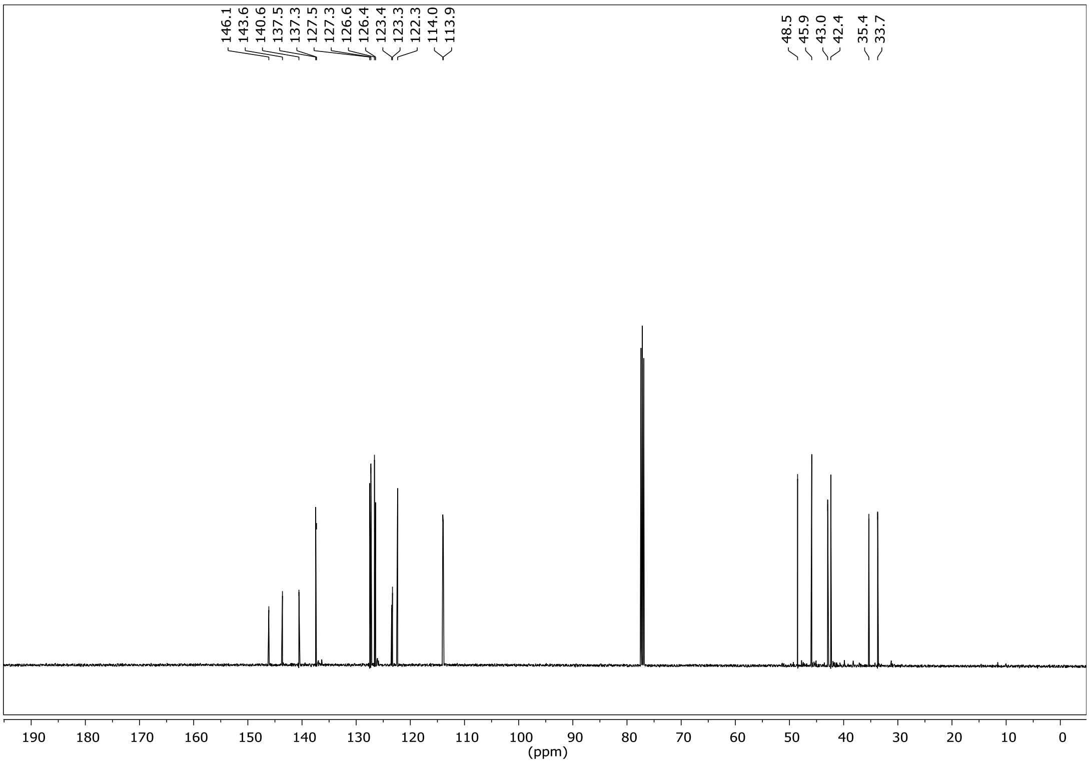

Figure S4. 125 MHz ^{13}C NMR spectrum of 3-norbornyl-*N*-methylphenothiazine (**2**) in $CDCl_3$.

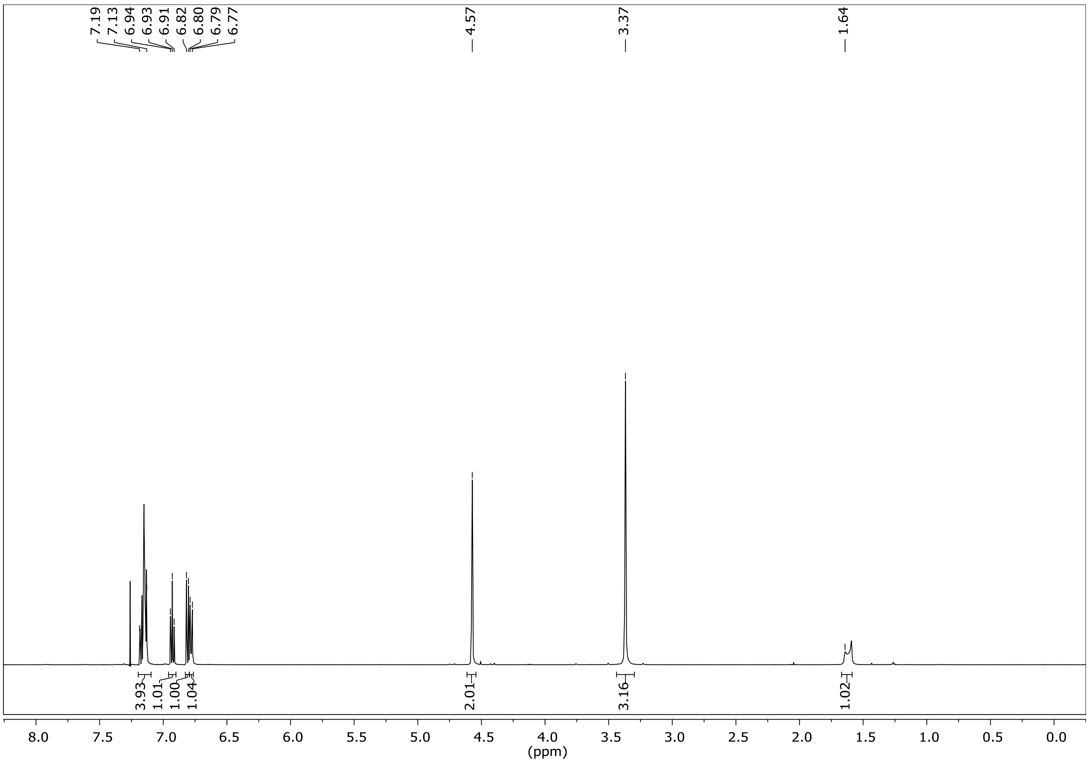

Figure S5. 500 MHz ^{1}H NMR spectrum of 3-hydroxymethyl-*N*-methylphenothiazine (**5**) in $CDCl_3$.

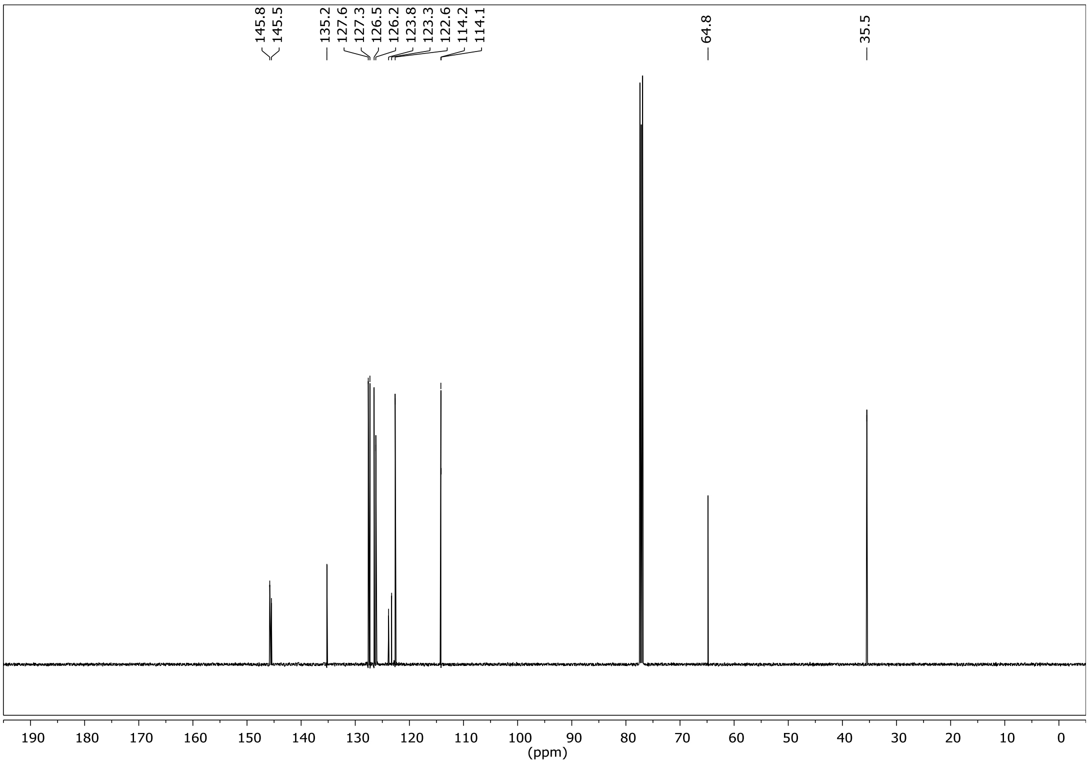

Figure S6. 125 MHz ^{13}C NMR spectrum of 3-hydroxymethyl-*N*-methylphenothiazine (**5**) in $CDCl_3$.

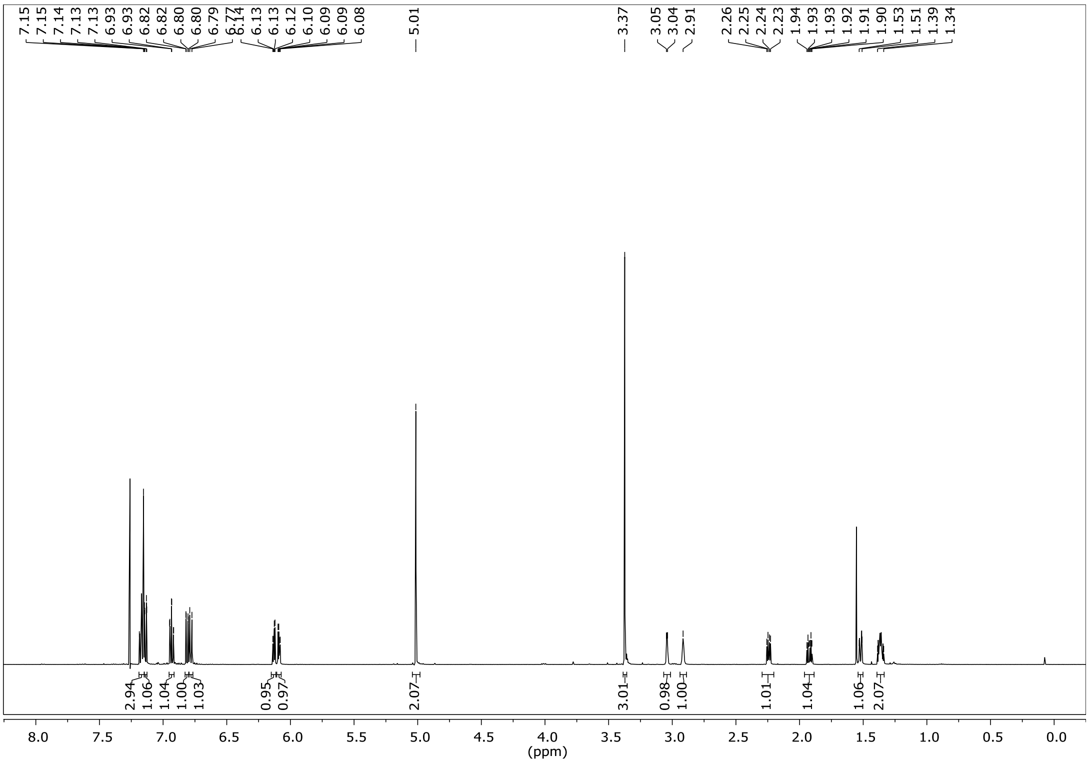

Figure S7. 500 MHz ^{1}H NMR spectrum of *exo*-5-norbornene-2-carboxylic acid (*N*-methylphenothiazin-3-yl)methyl ester (**6**) in $CDCl_3$.

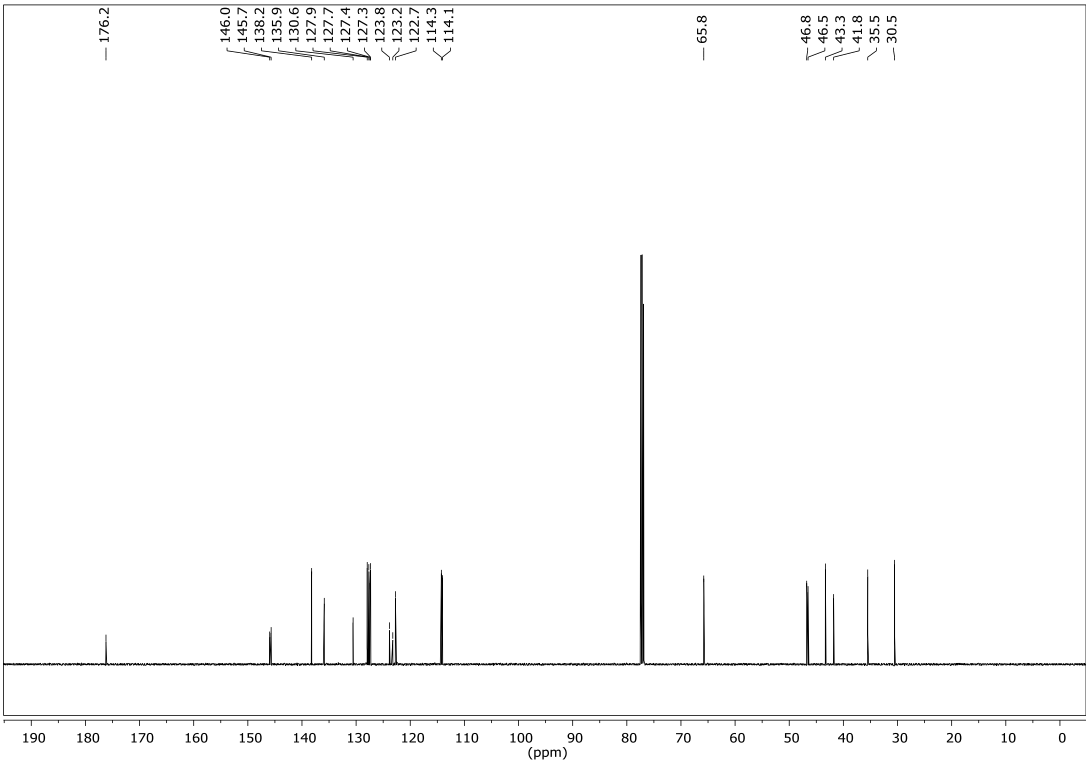

Figure S8. 125 MHz ^{13}C NMR spectrum of *exo*-5-norbornene-2-carboxylic acid (*N*-methylphenothiazin-3-yl)-methyl ester (**6**) in $CDCl_3$.

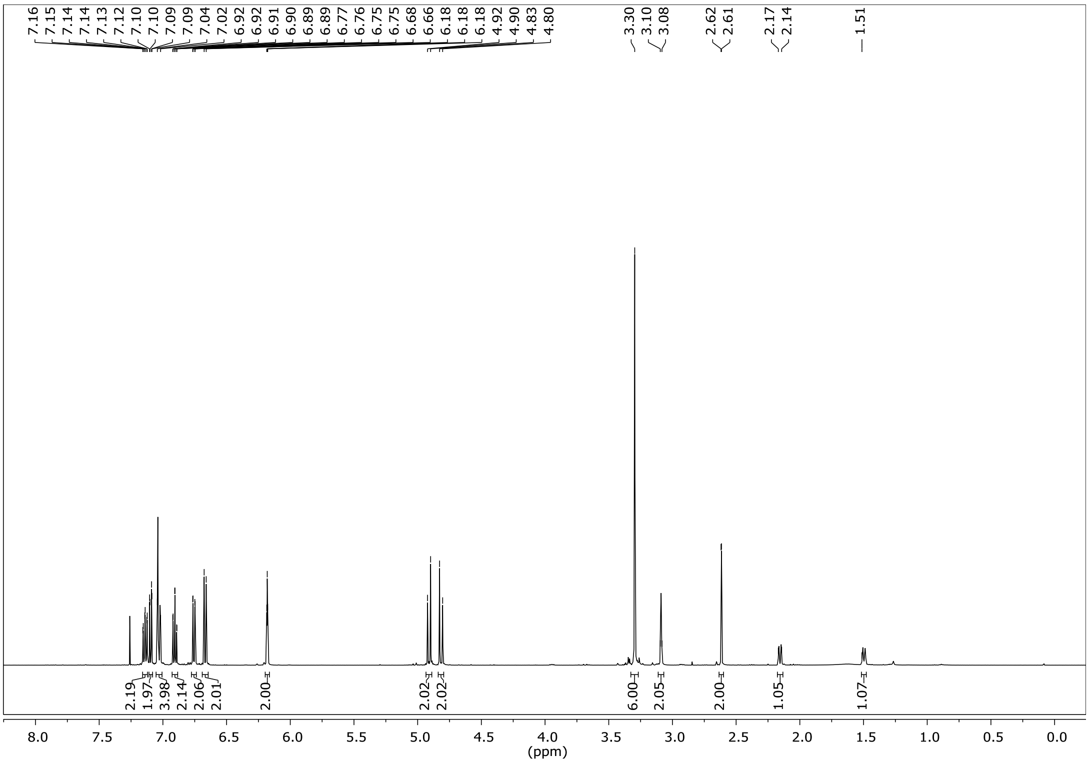

Figure S9. 500 MHz ^{1}H NMR spectrum of *cis*-*exo*-5-norbornene-2,3-dicarboxylic acid bis(*N*-methylphenothiazin-3-yl)methyl ester (**7**) in $CDCl_3$.

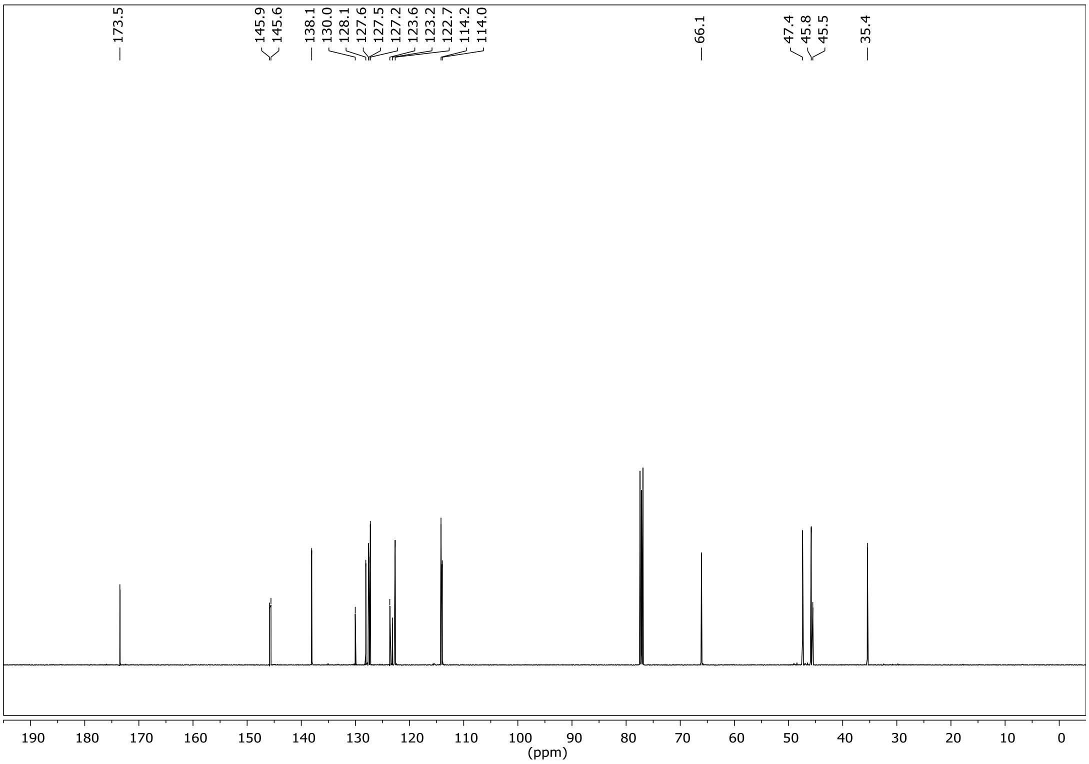

Figure S10. 125 MHz ^{13}C NMR spectrum of *cis*-*exo*-5-norbornene-2,3-dicarboxylic acid bis(*N*-methylphenothiazin-3-yl)methyl ester (**7**) in $CDCl_3$.

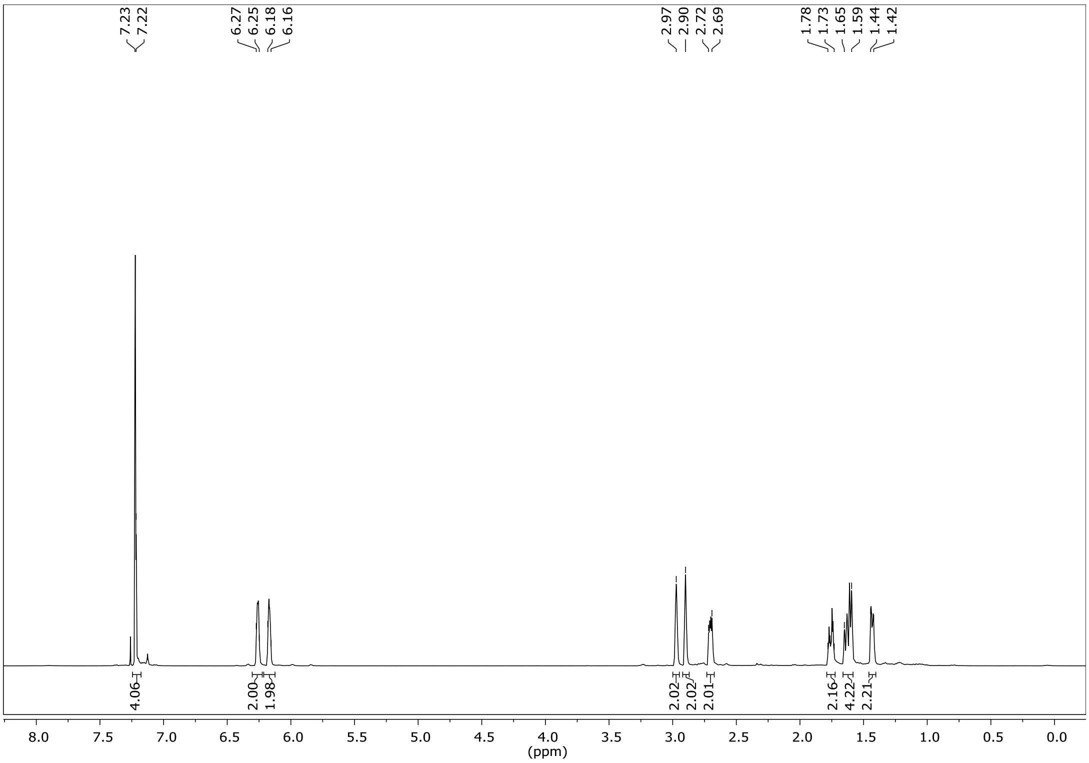

Figure S11. 500 MHz ^{1}H NMR spectrum of 1,4-di-(*exo*-norbornen-2-yl)benzene (**8**) in $CDCl_3$.

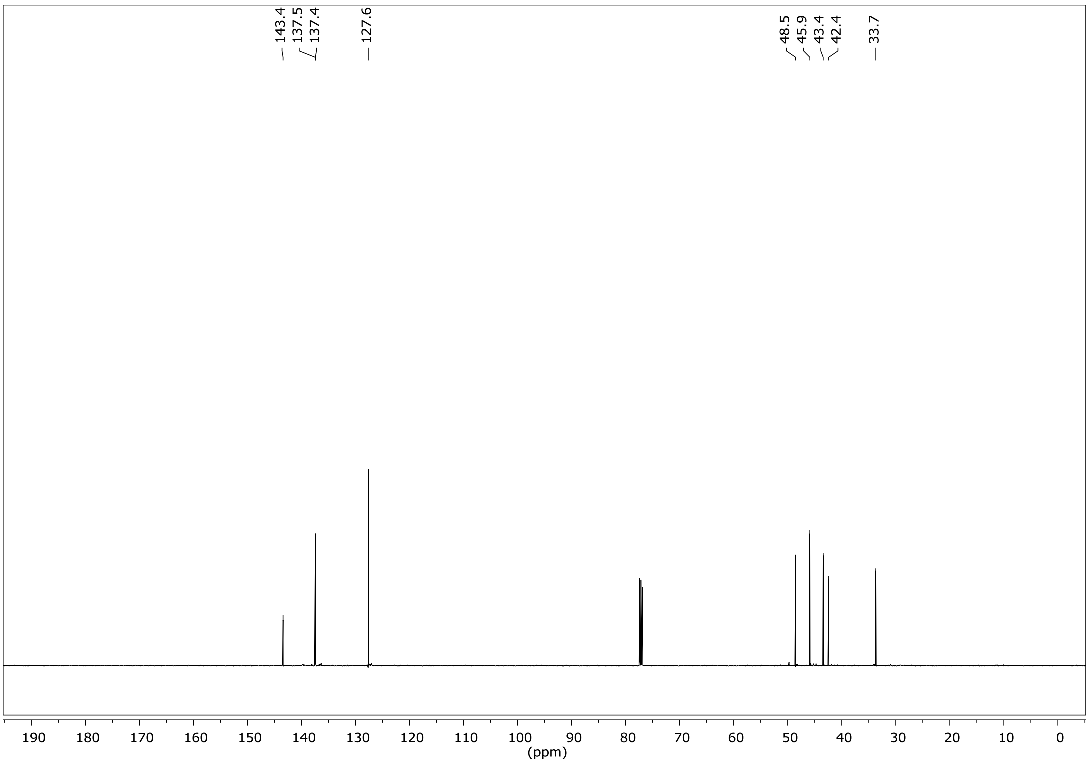

Figure S12. 125 MHz ^{13}C NMR spectrum of 1,4-di-(*exo*-norbornen-2-yl)benzene (**8**) in $CDCl_3$.

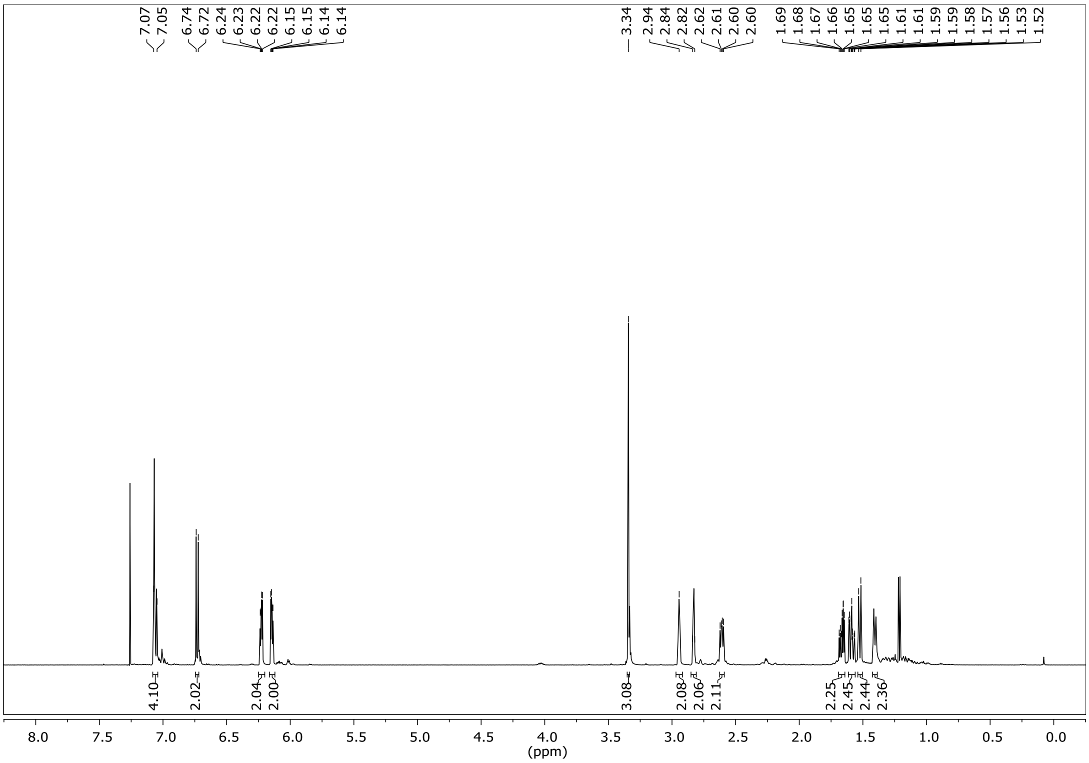

Figure S13. 400 MHz ^{1}H NMR spectrum of 3,7-dinorbornyl-*N*-methylphenothiazine (**4**) in $CDCl_3$.

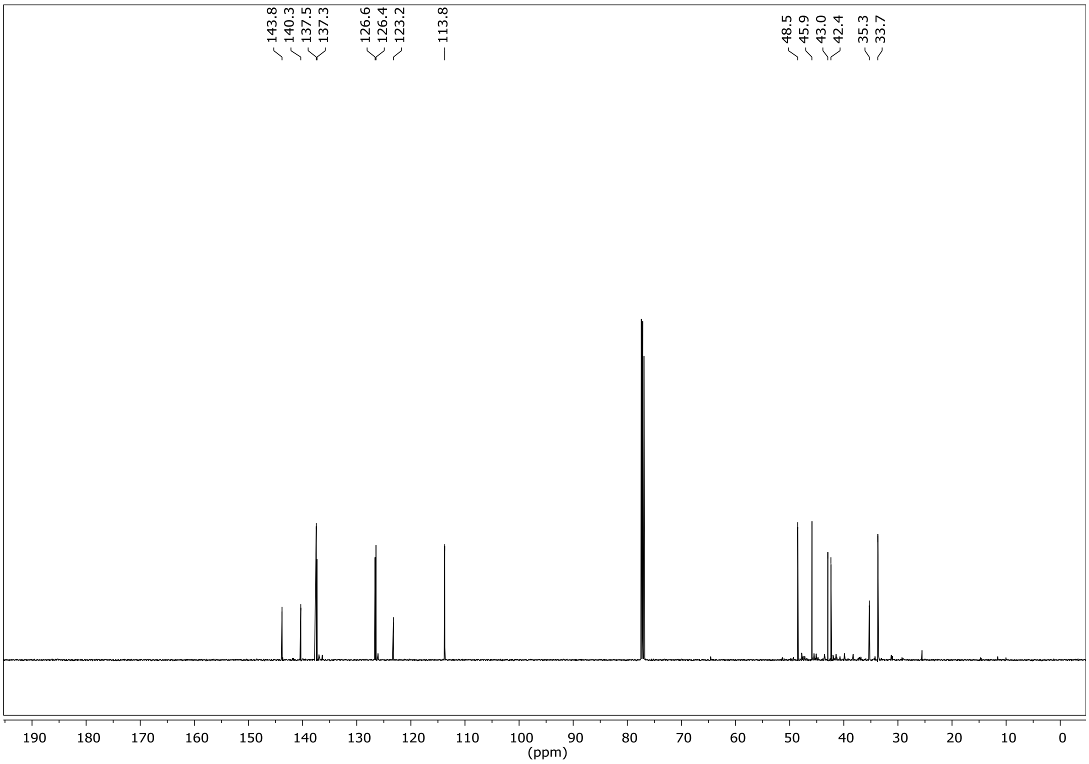

Figure S14. 100 MHz ^{13}C NMR spectrum of 3,7-dinorbornyl-*N*-methylphenothiazine (**4**) in $CDCl_3$.

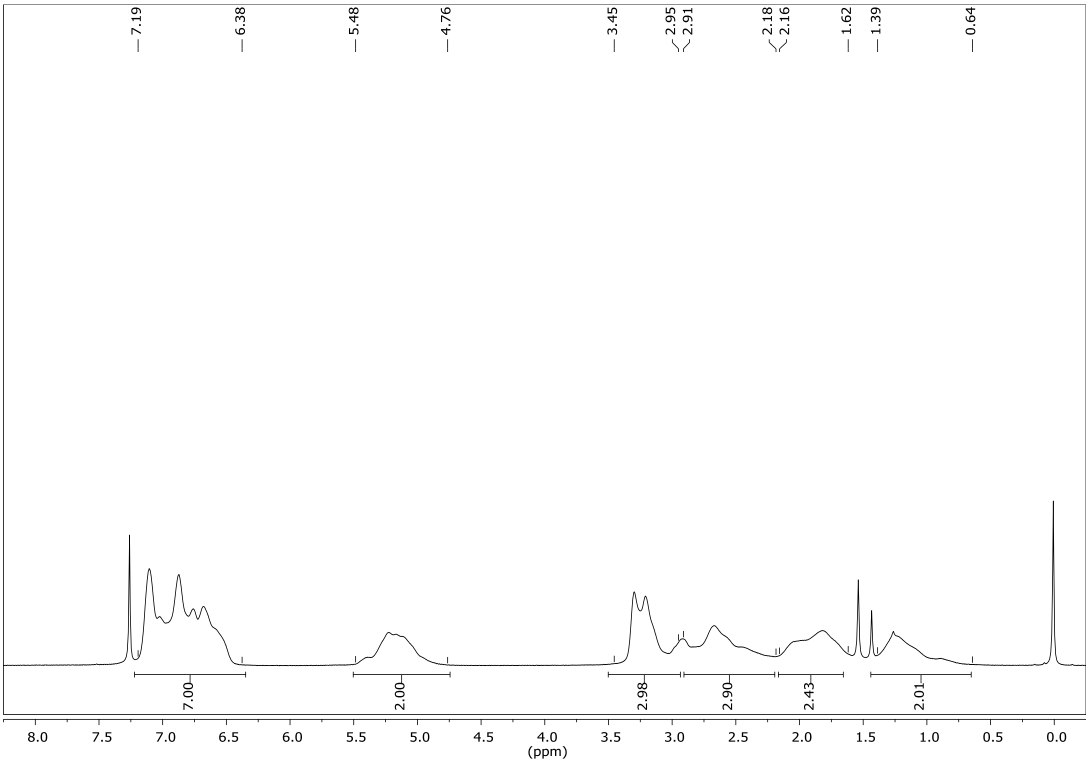

Figure S15. 400 MHz ^{1}H NMR spectrum of poly(3-norbornyl-*N*-methylphenothiazine) (**PNMPT**) in $CDCl_3$.

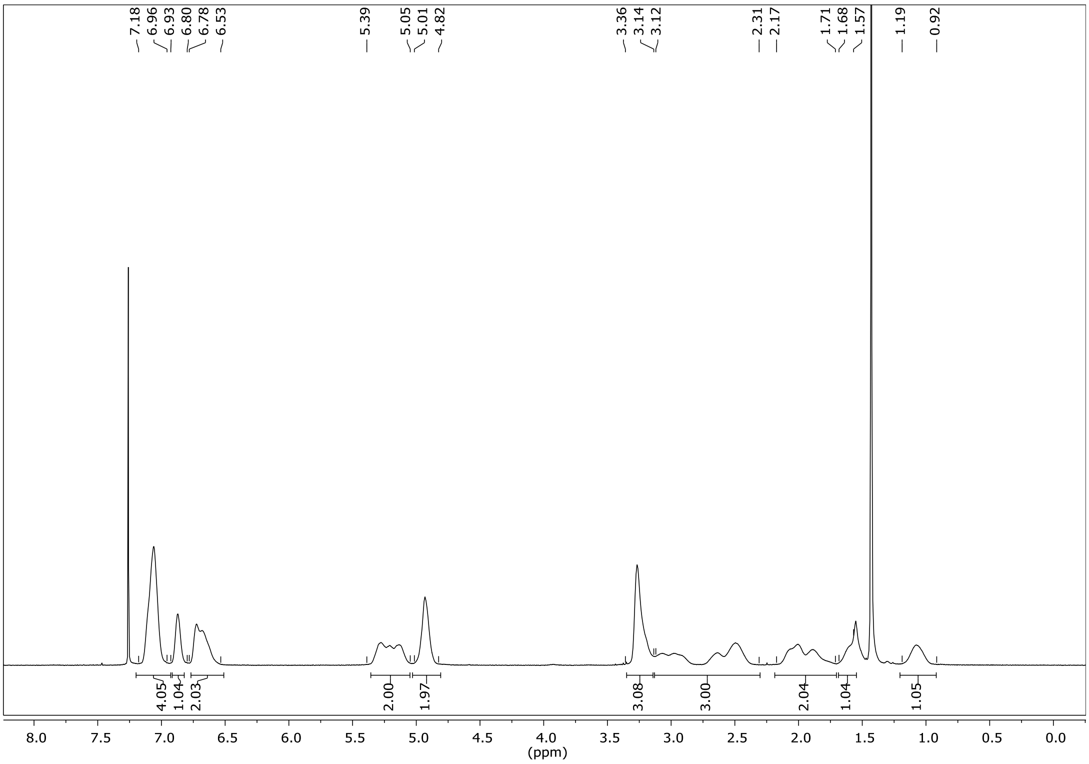

Figure S16. 500 MHz ^{1}H NMR spectrum of poly(*exo*-5-norbornene-2-carboxylic acid (*N*-methylphenothiazin-3-yl)methyl ester) (**P1**) in $CDCl_3$.

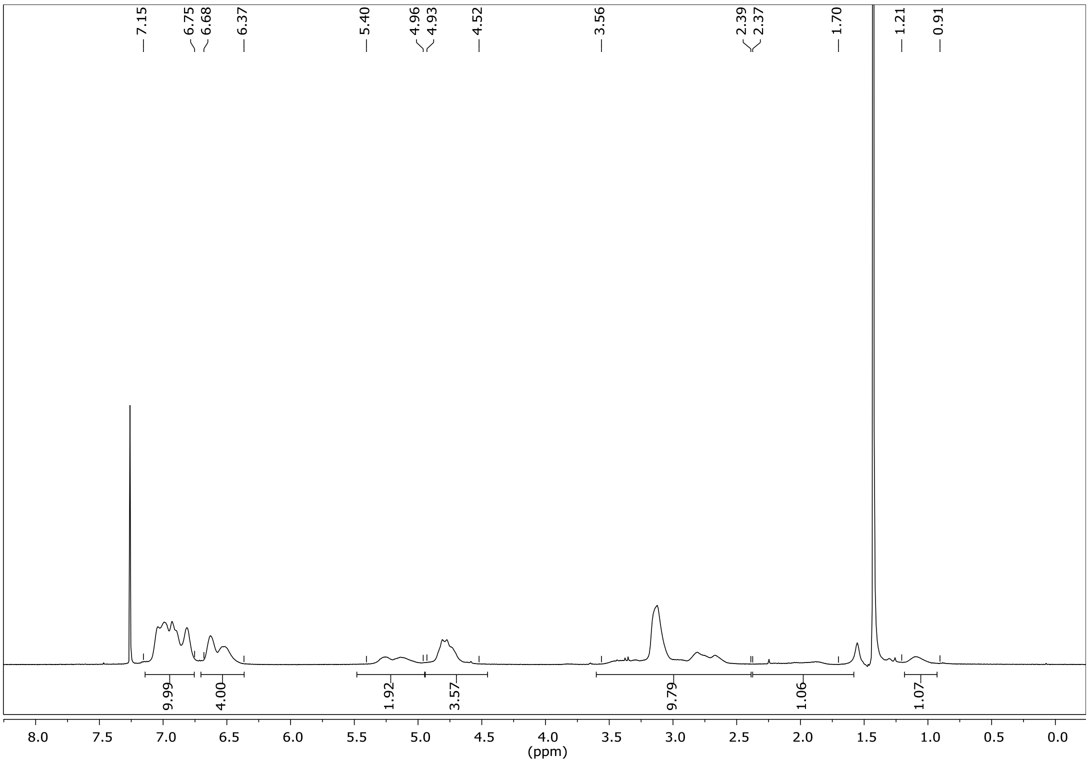

Figure S17. 500 MHz ^{1}H NMR spectrum of poly(*cis*-*exo*-5-norbornene-2,3-dicarboxylic acid bis(*N*-methyl-phenothiazin-3-yl)methyl ester) (**P2**) in $CDCl_3$.

1.3.2 Stereochemical determination of norbornyl-*N*-methylphenothiazine 2

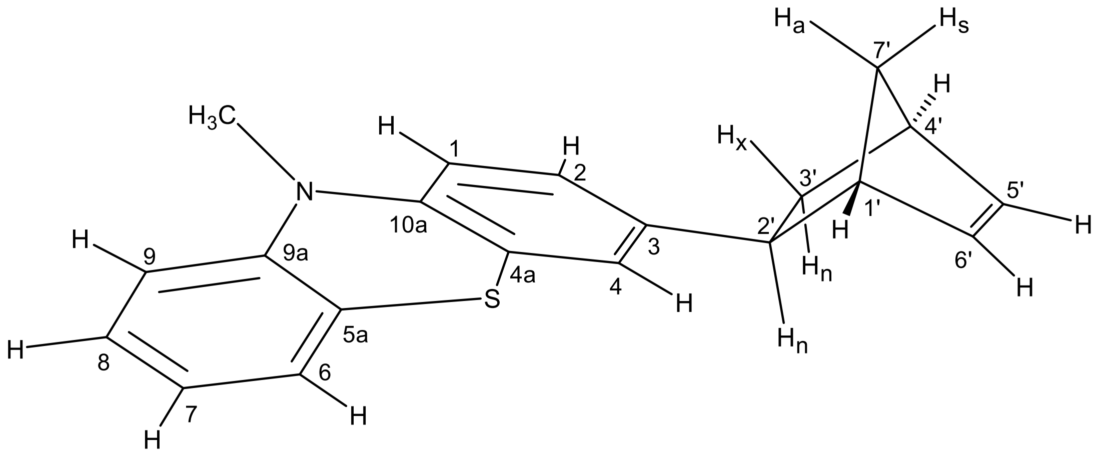

Table S5. Correlation of ^{1}H and ^{13}C NMR signals determined by 2D NMR experiments.

position	δ ^{1}H (ppm)	multiplicity with J (Hz)	intensity	δ ^{13}C (ppm)
7‘s	1.43–1.40	m	1	45.9
7‘a	1.54–1.53	m	1	45.9
3‘n	1.60	ddd, *J* = 11.4, 8.8, 2.4 Hz	1	33.7
3‘x	1.68	ddd, *J* = 11.8, 4.7, 3.5 Hz	1	33.7
2‘n	2.62	dd, *J* = 8.7, 4.7 Hz	1	43.0
1‘	2.85–2.84	m	1	48.5
4‘	2.95	br. s	1	42.4
N-CH_3	3.36	s	3	35.4
5‘	6.15	dd, *J* = 5.7, 2.9 Hz	1	137.5
6‘	6.23	dd, *J* = 5.7, 3.1 Hz	1	137.3
1	6.75	d, *J* = 9.0 Hz	1	113.9
9/6	6.80	dd, *J* = 8.0, 0.9 Hz	1	114.0
7/8	6.92	ddd, *J* = 7.5, 7.5, 1.2 Hz	1	122.3
2, 4	7.08–7.06	m	2	126.4, 126.6
9/6, 7/8	7.18–7.14	m	2	127.3, 127.5
4a/5a				123.3
4a/5a				123.4
9a/10a				143.6
9a/10a				146.1

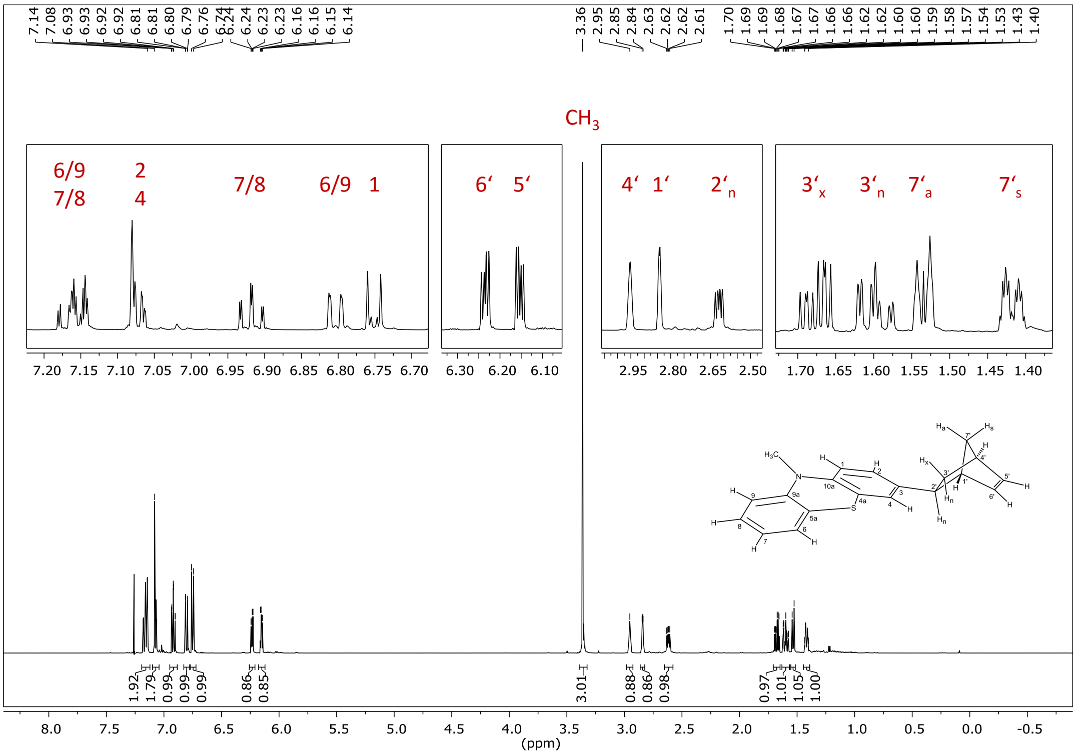

Figure S18. 500 MHz ^{1}H NMR spectrum of 3-norbornyl-*N*-methylphenothiazine (**2**) in $CDCl_3$.

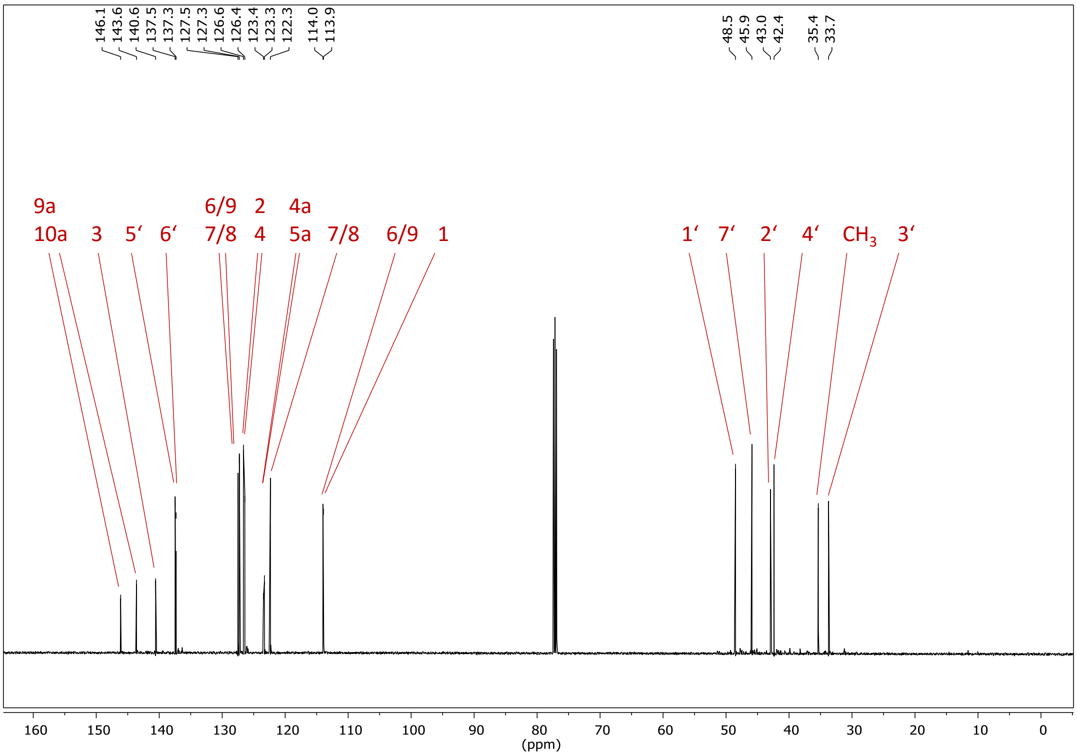

Figure S19. 125 MHz ^{13}C NMR spectrum of 3-norbornyl-*N*-methylphenothiazine (**2**) in $CDCl_3$.

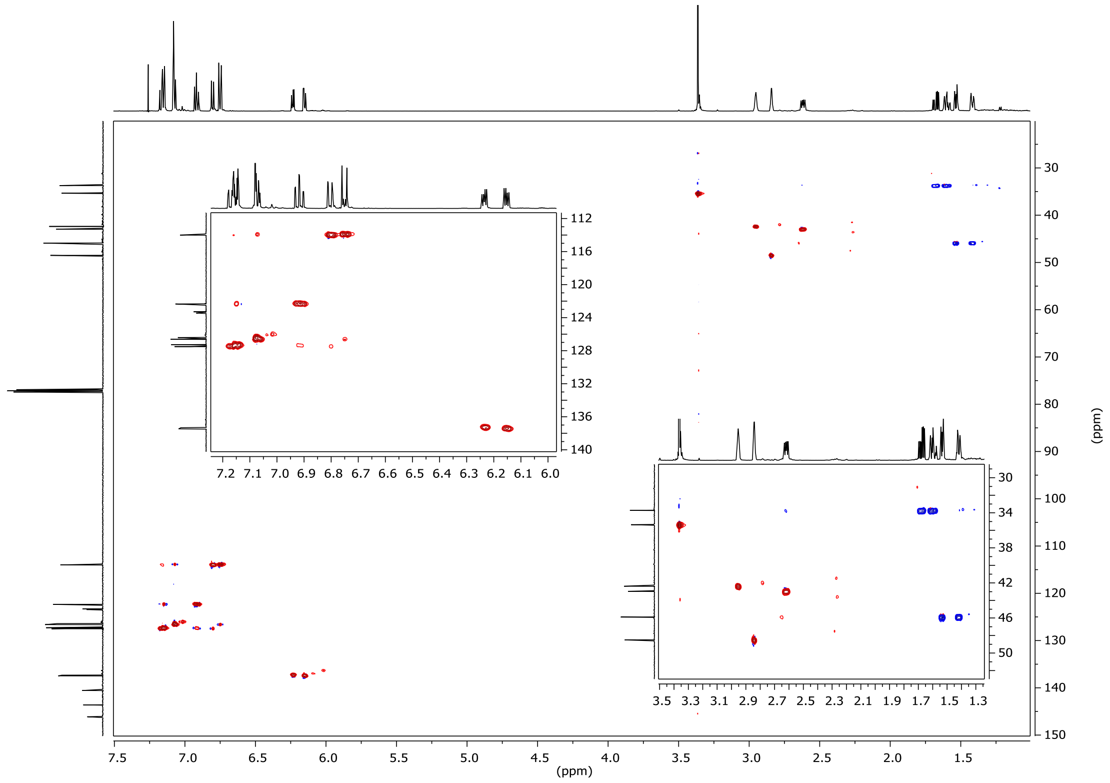

Figure S20. HSQC 2D NMR experiment of 3-norbornyl-*N*-methylphenothiazine (**2**) in $CDCl_3$.

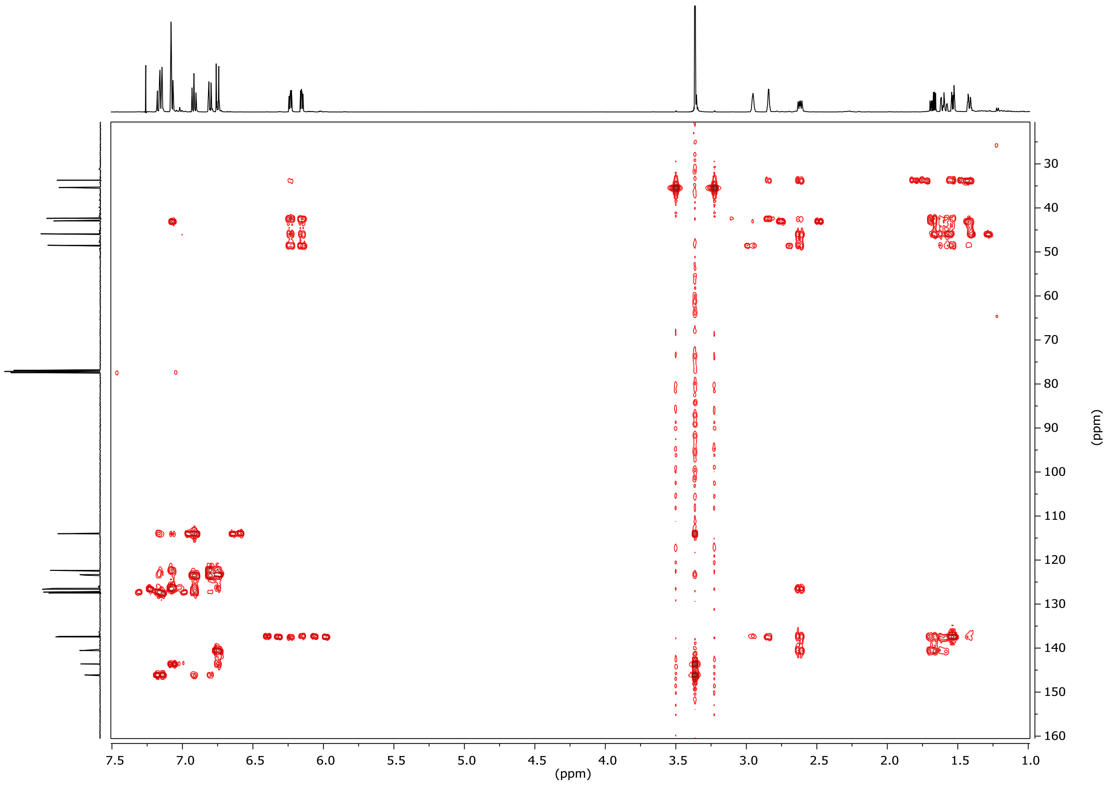

Figure S21. HMBC 2D NMR experiment of 3-norbornyl-*N*-methylphenothiazine (**2**) in $CDCl_3$.

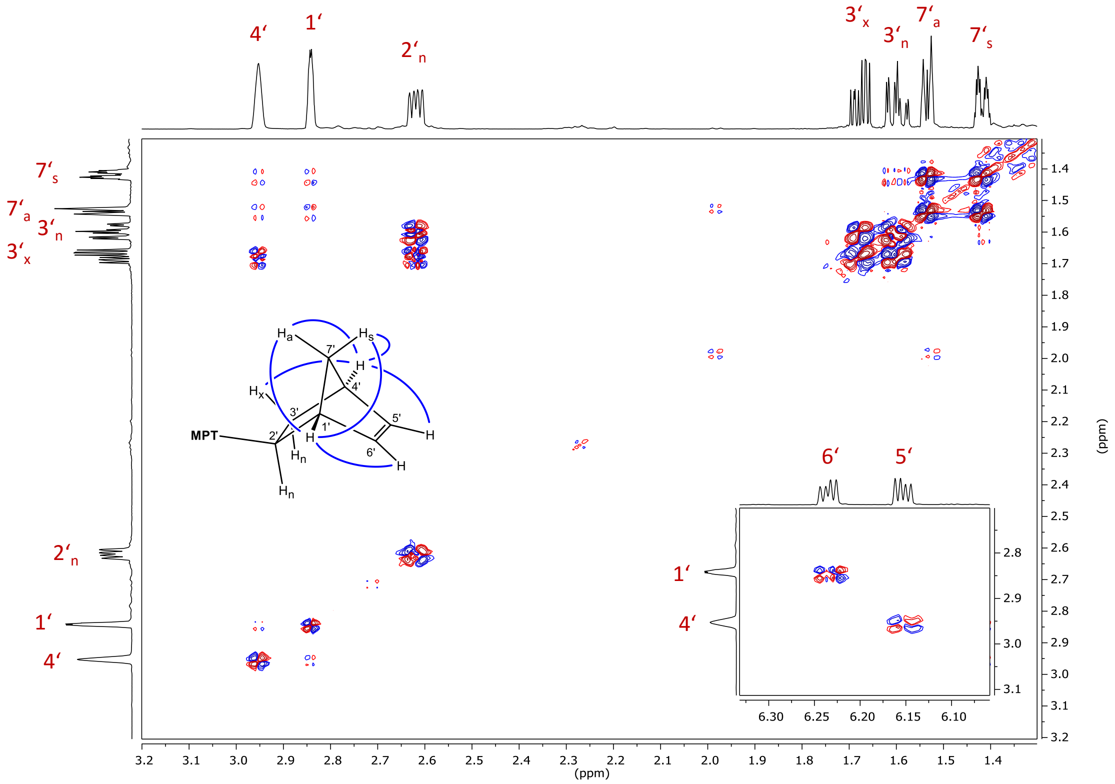

Figure S22. DQF-Cosy 2D NMR experiment of 3-norbornyl-*N*-methylphenothiazine (**2**) in $CDCl_3$.

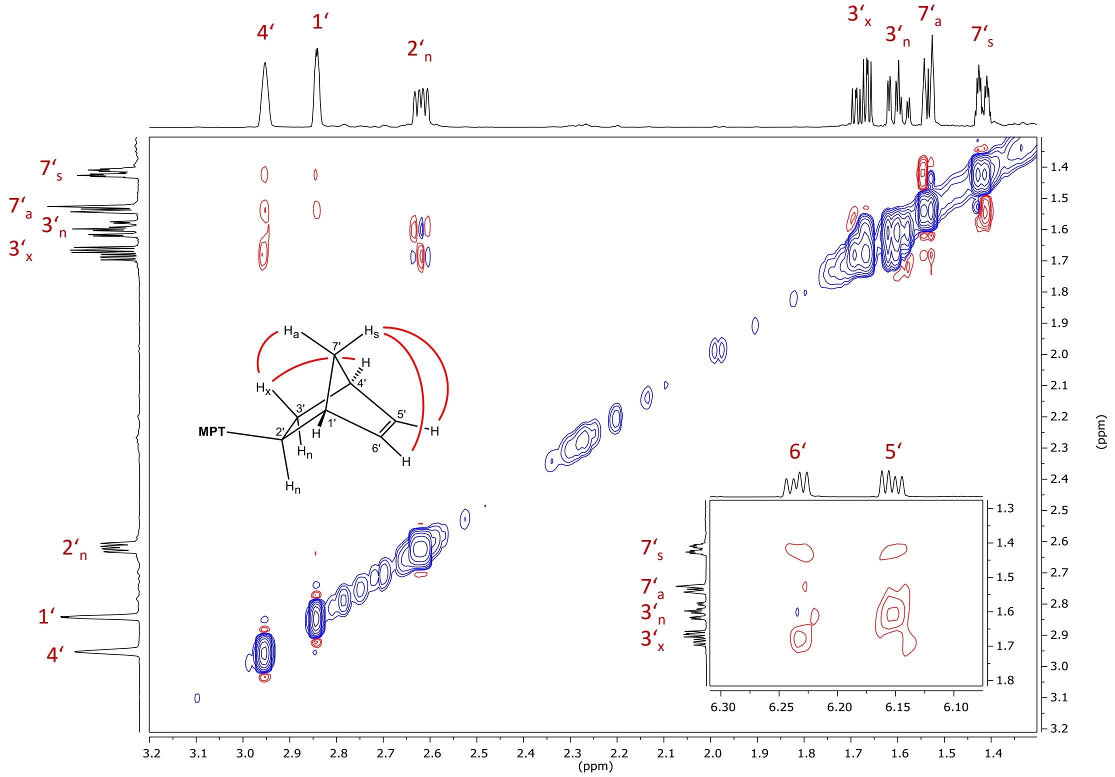

Figure S23. NOESY 2D NMR experiment of 3-norbornyl-*N*-methylphenothiazine (**2**) in $CDCl_3$.

1.3.3 FT-IR spectra of MPT poly(norbornene)s

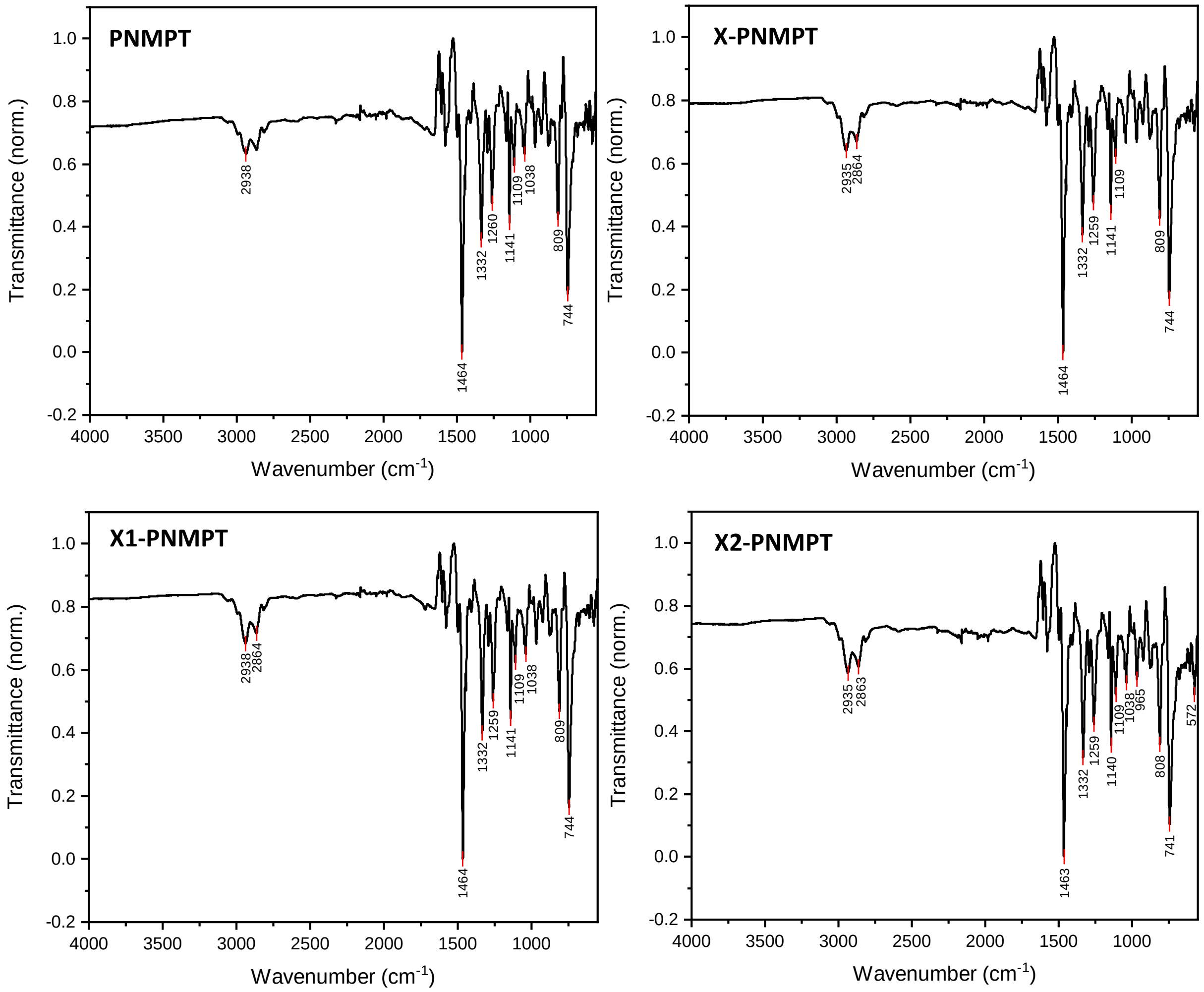

Figure S24. ATR FT-IR spectra (diamond, 64 scans, res. 2 cm^{-1}) of **PNMPT** and its cross-linked derivatives **X-PNMPT**, **X1-PNMPT** and **X2-PNMPT**.

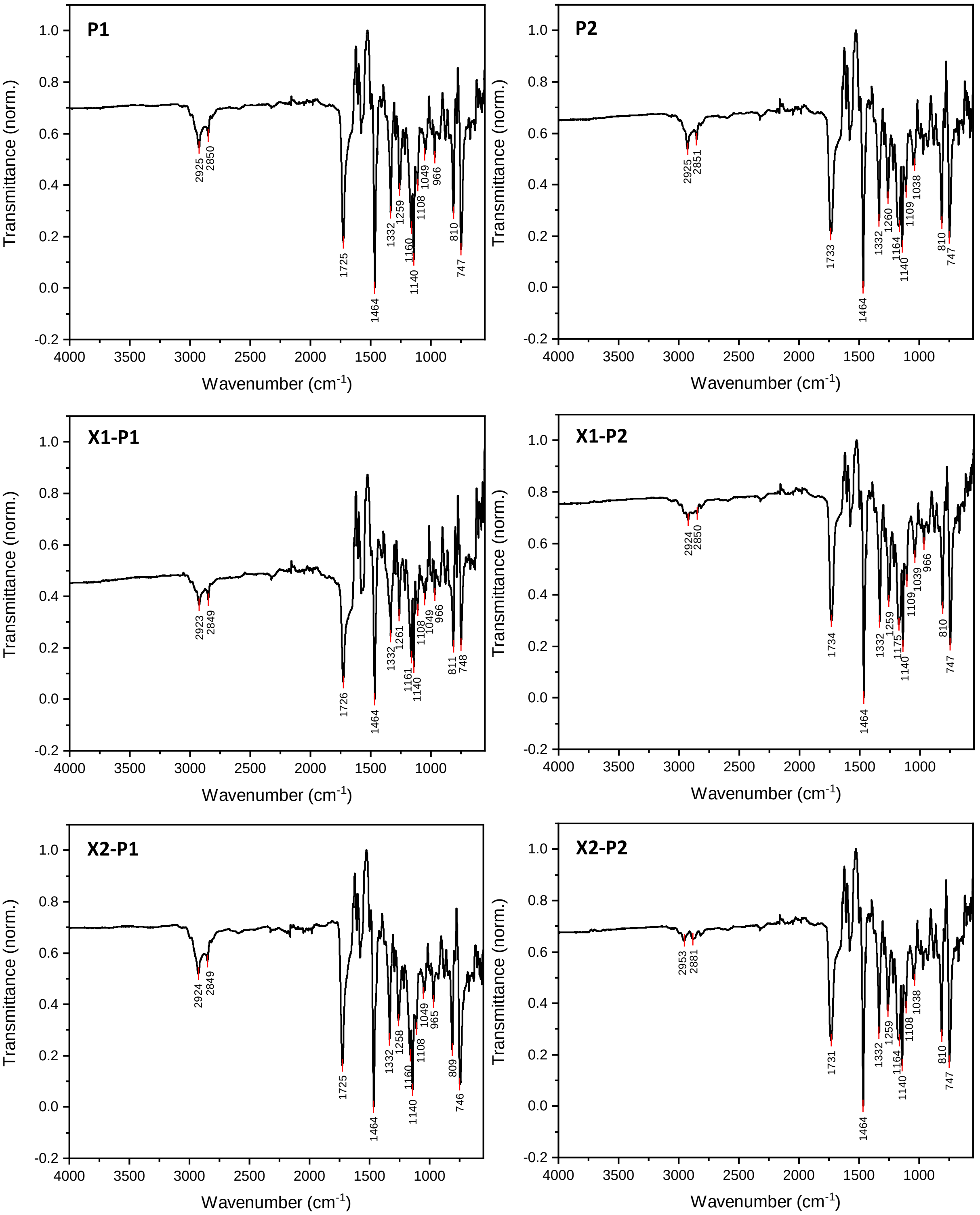

Figure S25. ATR FT-IR spectra (diamond, 64 scans, res. 2 cm^{-1}) of the poly(norbornyl ester) polymers **P1** and **P2** as well as their cross-linked derivatives **X1-P1**, **X2-P1**, **X1-P2** and **X2-P2**.

1.3.4 Thermal gravimetric analyses (TGA) of MPT poly(norbornene)s

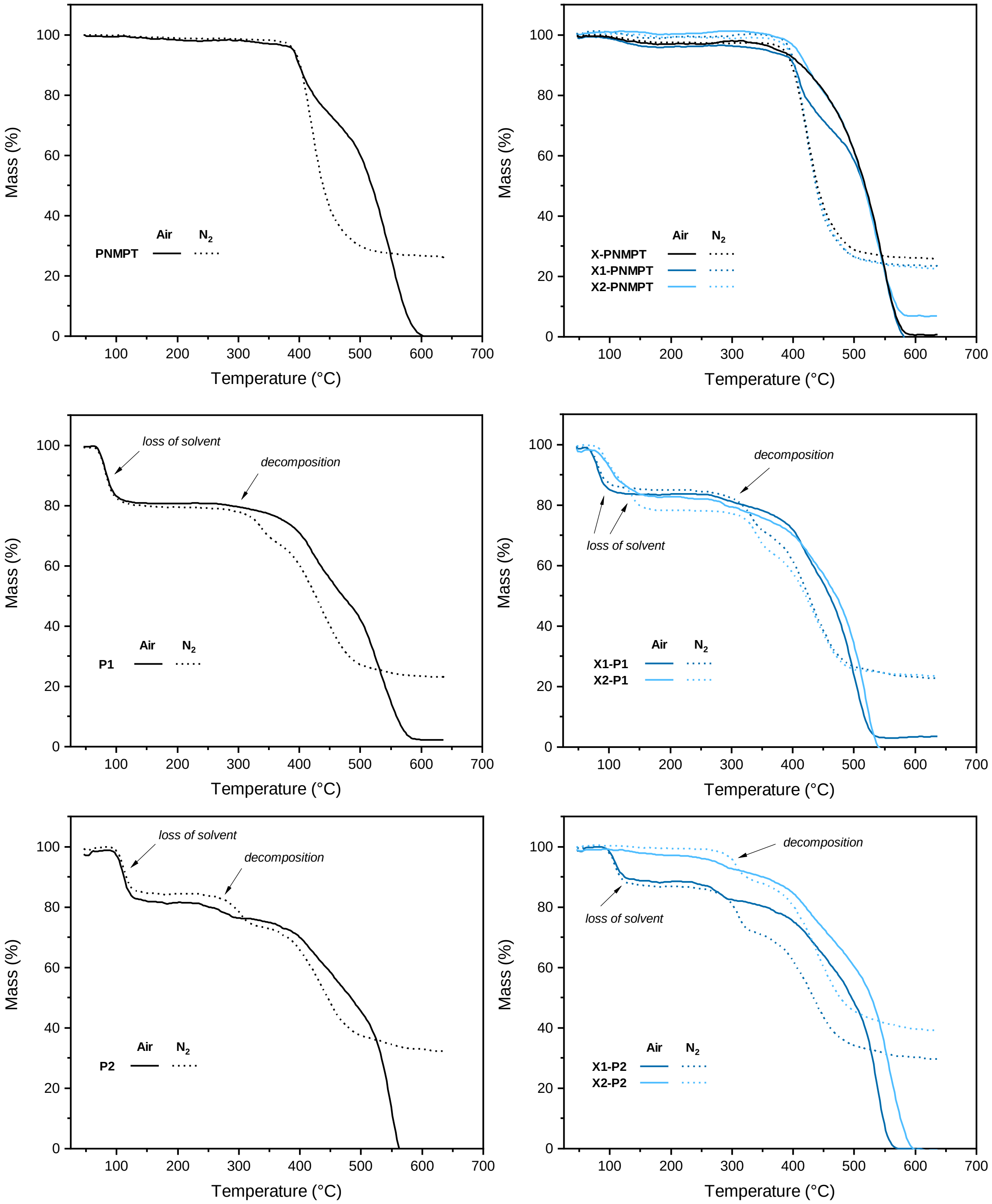

Figure S26. TGA curves of **PNMPT**, **P1** and **P2** (from top to bottom, left) as well as their cross-linked derivatives (right) in ambient air atmosphere and N_2 at a heating rate of 10 °C min^{-1}. For investigations in battery test cells, the samples were further dried at 60 °C under high vacuum to remove remaining solvent (see chapter 3.1).

1.3.5 Differential scanning calorimetry (DSC) measurements of MPT poly(norbornene)s

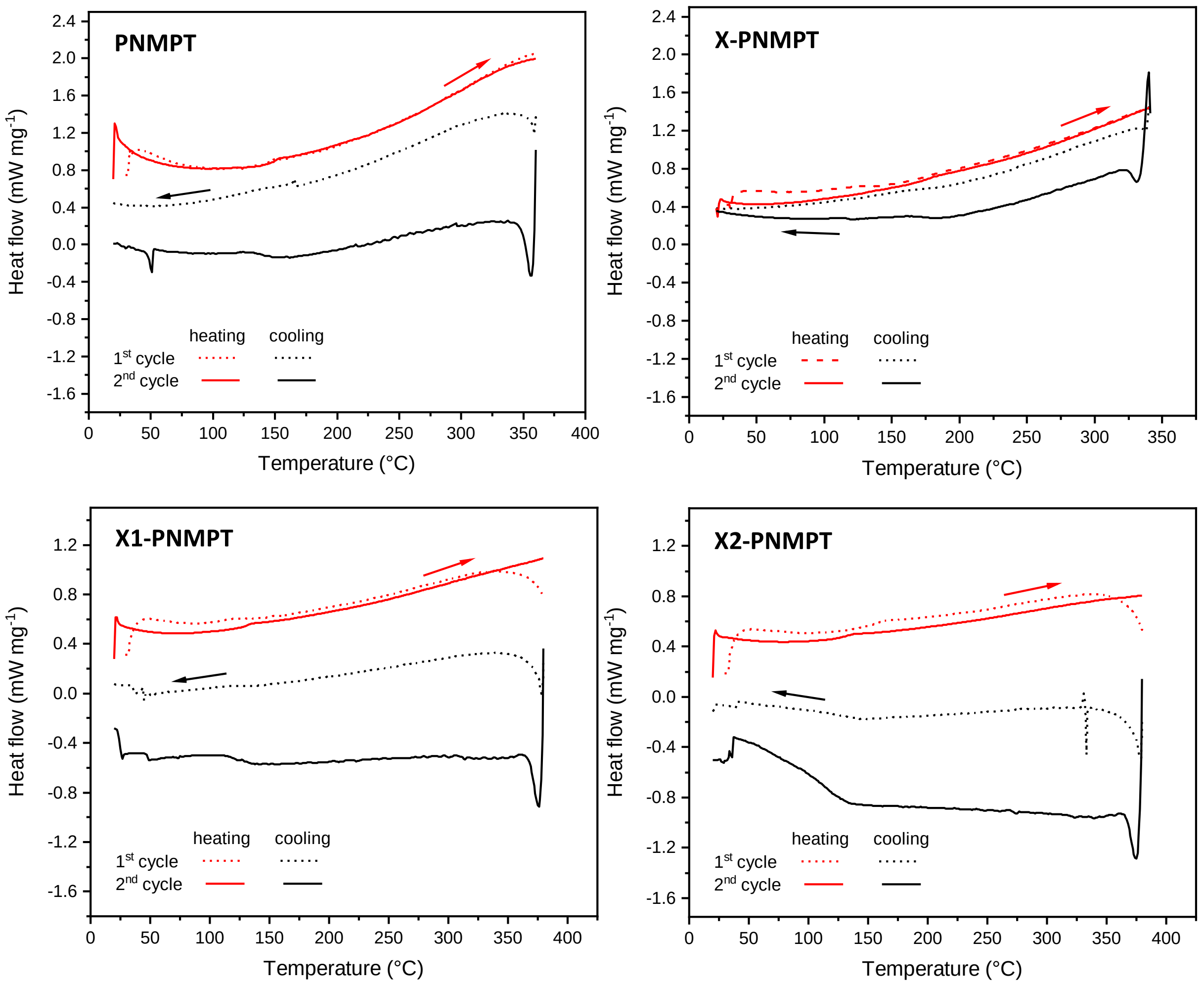

Figure S27. DSC measurements of **PNMPT** and its cross-linked derivatives **X-PNMPT**, **X1-PNMPT** and **X2-PNMPT** at a heating rate of 10 °C min^{-1}.

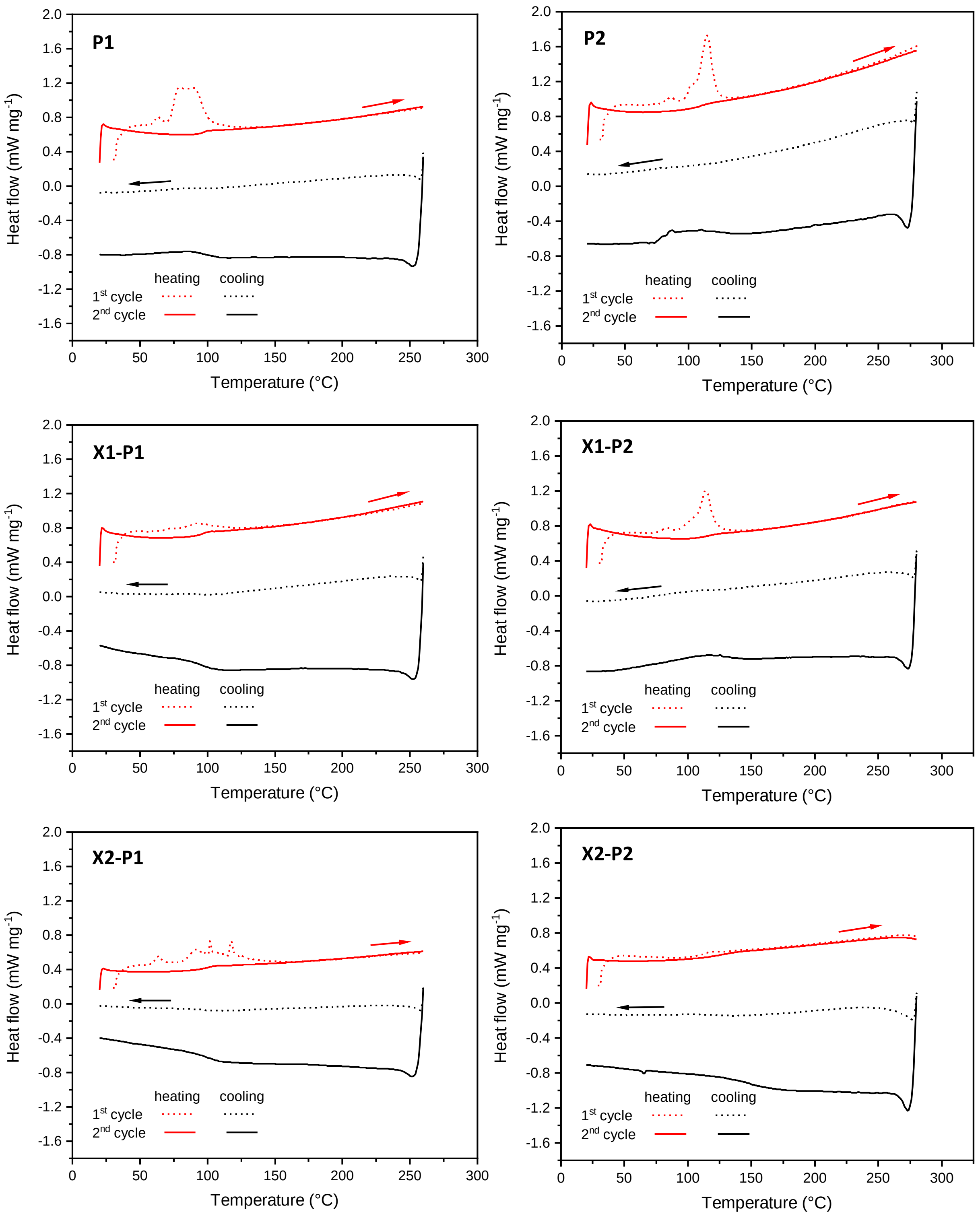

Figure S28. DSC measurements of the poly(norbornyl ester) polymers **P1** (left) and **P2** (right) as well as their cross-linked derivatives **X1-P1**, **X2-P1**, **X1-P2** and **X2-P2** at a heating rate of 10 °C min^{-1}.

1.3.6 Solubility tests of PNMPT, P1 and P2

Solubility tests of **PNMPT**, **P1** and **P2** and their oxidized forms were carried out under an argon atmosphere in the standard battery electrolyte (EC:DMC 1:1 with 1 M $LiPF_6$). Oxidation of the polymers was carried out in dichloromethane by combining the polymer solution or suspension with an appropriate solution of the oxidant silver hexafluorophosphate (0.5 eq or 1.0 eq, corresponding to a 50% oxidized or 100% oxidized sample, respectively). The resulting purple-colored hexafluoro-phosphate polymer salts precipitated, and the solvent was removed by evaporation at room temperature overnight. The neutral or oxidized polymers (0.31 mg **PNMPT**, 0.36 mg **P1** and 0.32 mg **P2**, respectively) were diluted with the standard battery electrolyte (1.0 mL). After several days no change in solubility as judged by color intensity was observed.

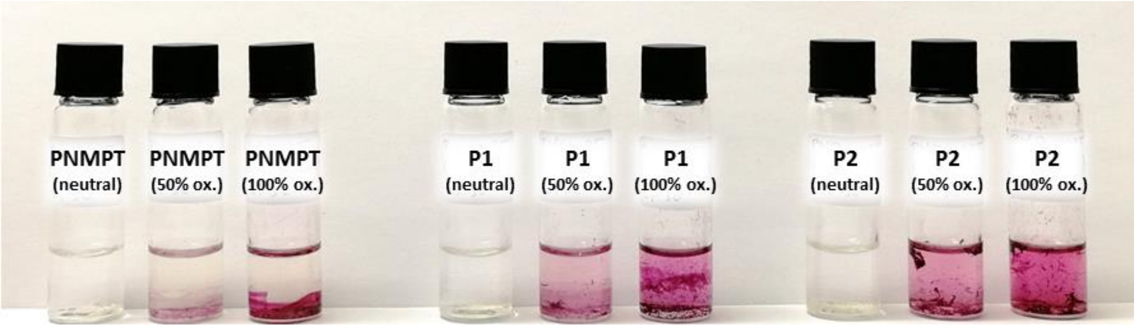

Figure S29. Solubility tests of **PNMPT** (left), **P1** (middle) and **P2** (right) with different oxidation degrees (each from left to right: neutral, 50% oxidized and 100% oxidized) in the battery electrolyte EC:DMC 1:1 with 1 M $LiPF_6$.

2 Spectroscopic and electrochemical investigations

2.1 UV/Vis/NIR spectroscopy

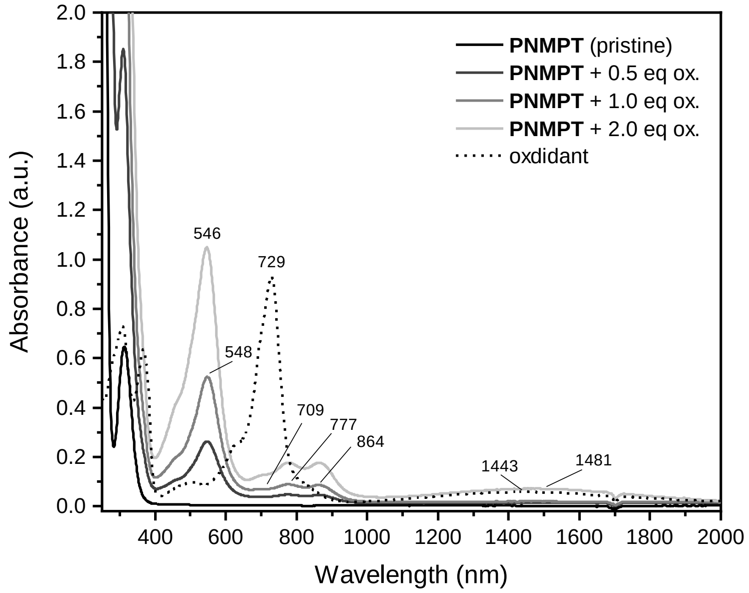

Figure S30. UV/Vis/NIR spectra of **PNMPT** in its pristine state and after the addition of 0.5, 1.0 and 2.0 equivalents of the oxidant tris-(4-bromophenyl)-ammoniumyl hexachloroantimonate in CH_2Cl_2 (0.1 mM referred to the redox active group).

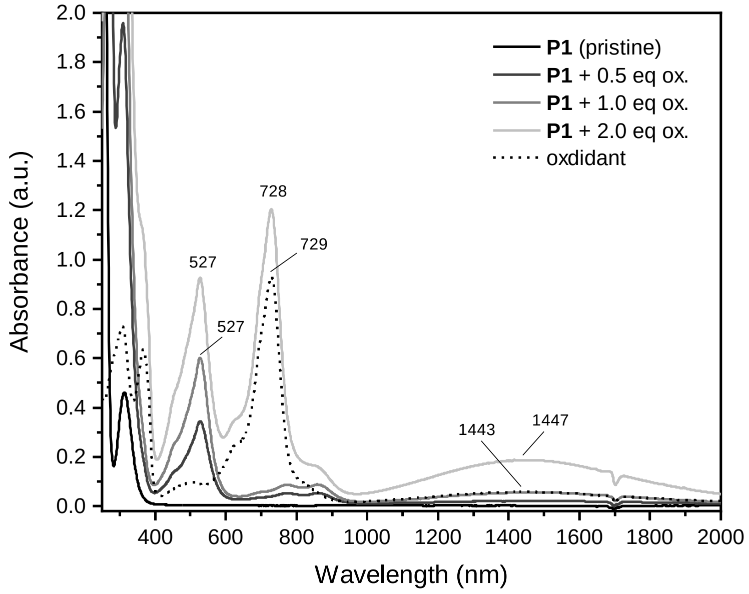

Figure S31. UV/Vis/NIR spectra of **P1** in its pristine state and after the addition of 0.5, 1.0 and 2.0 equivalents of the oxidant tris-(4-bromophenyl)-ammoniumyl hexachloroantimonate in CH_2Cl_2 (0.1 mM referred to the redox active group).

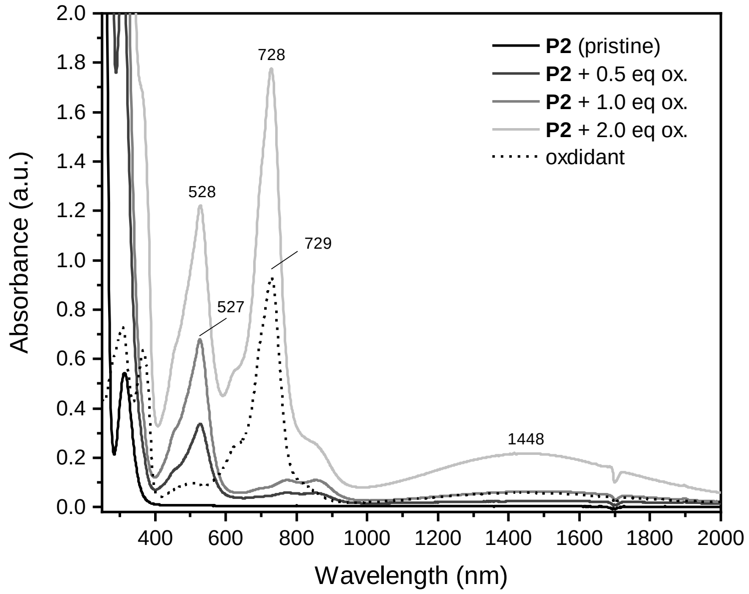

Figure S32. UV/Vis/NIR spectra of **P2** in its pristine state and after the addition of 0.5, 1.0 and 2.0 equivalents of the oxidant tris-(4-bromophenyl)-ammoniumyl hexachloroantimonate in CH_2Cl_2 (0.1 mM referred to the redox active group).

2.2 Cyclic voltammetry in solution

a) b)

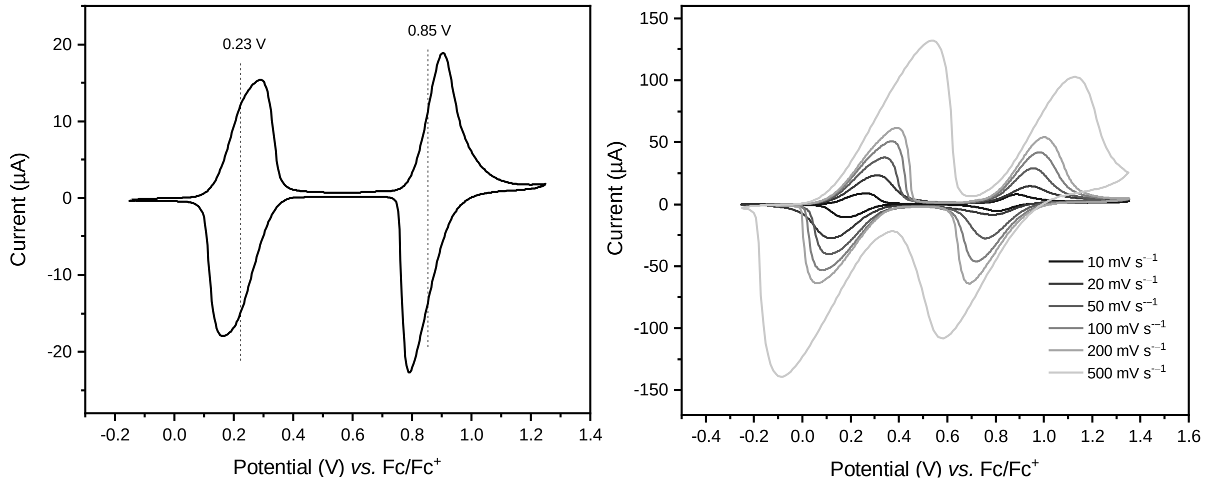

Figure S33. a) Cyclic voltammogram at 100 mV s^{-1} and b) scan rate test of **PNMPT**. Measurements performed in CH_2Cl_2, 1 mM (referred to the redox active group) with 0.1 M *n*-Bu_4NPF_6; WE: GC, CE: Pt, RE: Ag/$AgNO_3$ (referenced to internal standard Fc/Fc^+).

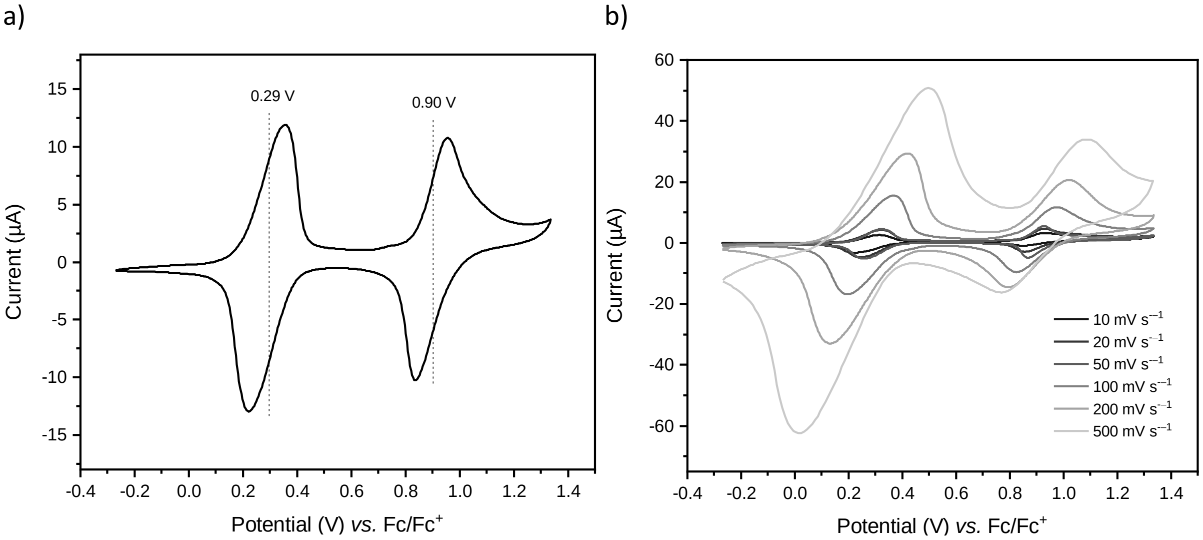

Figure S34. a) Cyclic voltammogram at 100 mV s^{-1} and b) scan rate test of **P1**. Measurements performed in CH_2Cl_2, 1 mM (referred to the redox active group) with 0.1 M *n*-Bu_4NPF_6; WE: GC, CE: Pt, RE: $Ag/AgNO_3$ (referenced to internal standard Fc/Fc^+).

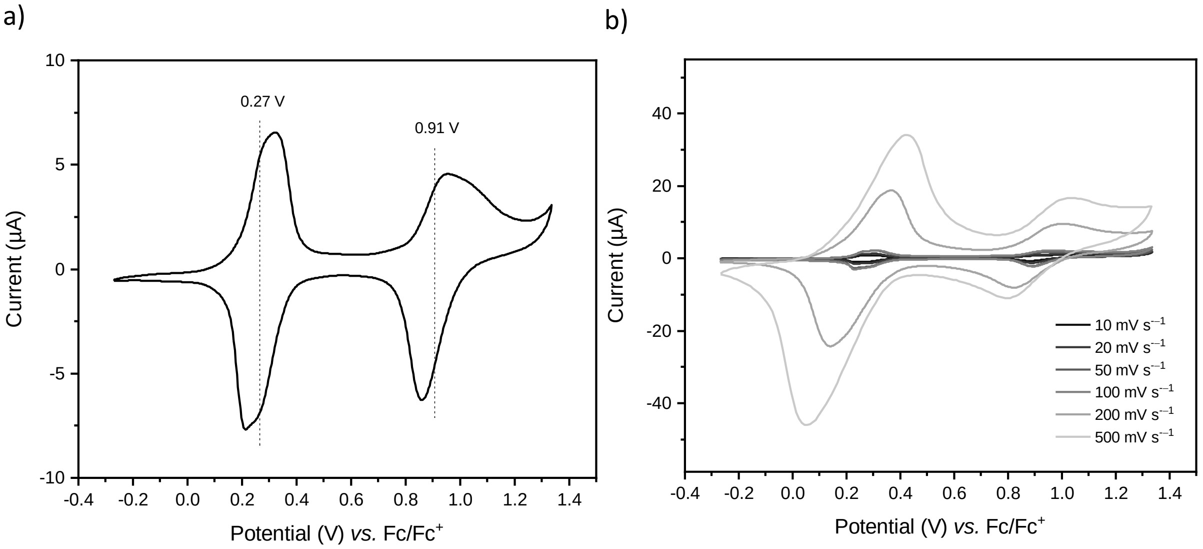

Figure S35. a) Cyclic voltammogram at 100 mV s^{-1} and b) scan rate test of **P2**. Measurements performed in CH_2Cl_2, 1 mM (referred to the redox active group) with 0.1 M *n*-Bu_4NPF_6; WE: GC, CE: Pt, RE: $Ag/AgNO_3$ (referenced to internal standard Fc/Fc^+).

3 Investigations on poly(norbornene)-based composite electrodes

3.1 Fabrication of composite electrodes

Electrode preparation was performed in a dry room with less than 0.02% air moisture. The electrodes were prepared using the same process for all polymers. Predried 50 wt% MPT-polymer, 45 wt% carbon black (*Super C65*, IMERYS) and 5 wt% PVdF (*KynarFlex 761a*, ARKEMA) were mixed and dissolved in *N*-methyl-2-pyrrolidone (NMP, SIGMA-ALDRICH, stored over molecular sieves). The electrode formulation was stirred for 24 h and then cast onto a KOH-etched aluminum foil (GOODFELLOW, thickness: 20 µm, >99.8%), using a blade-coating technique (wet film thickness: 50 µm; coating speed: 50 mm s^{-1}). The resulting coated foils were dried at ambient pressure for 4 h at 60 °C in a drying oven. Afterwards, electrodes with a diameter of 12 mm were punched out and dried *in vacuo* (10^{-3} mbar, 60 °C, 24 h). The mass loadings of pristine electrodes laid between 0.09–0.27 mg cm^{-2}.

3.2 SEM investigation of pristine MPT poly(norbornene)-based electrodes

Scanning electron microscopy (SEM) micrographs were recorded on a ZEISS *AURIGA CrossBeam Workstation* with an INLENS detector (SE-detector). 3 kV accelerating voltage was used at a working distance of 3 mm.

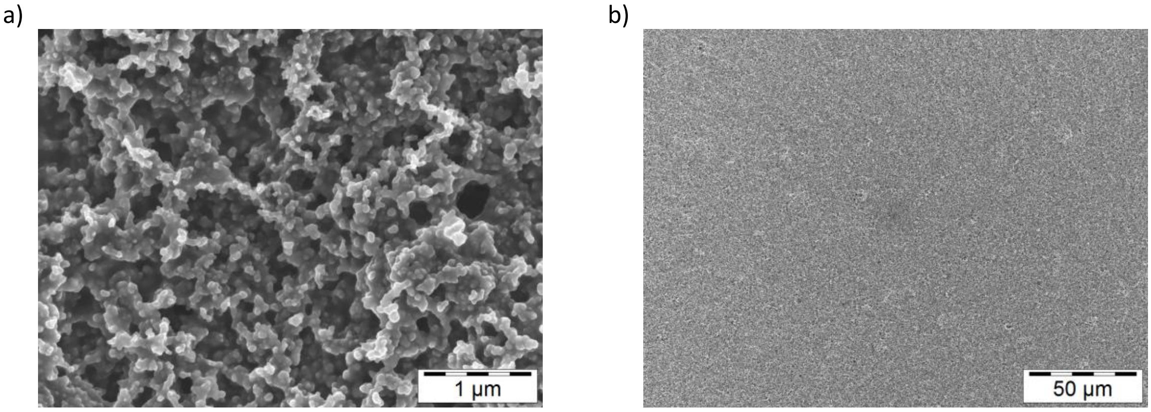

Figure S36. SEM micrographs of a pristine **X-PNMPT**-based electrode at a) 25000× magnification and b) 500× magnification.

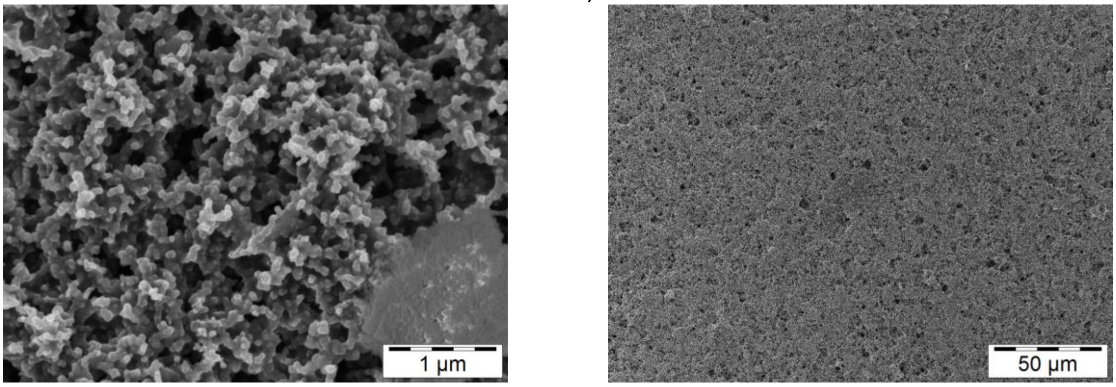

Figure S37. SEM micrographs of a pristine **X1-PNMPT**-based electrode at a) 25000× magnification and b) 500× magnification.

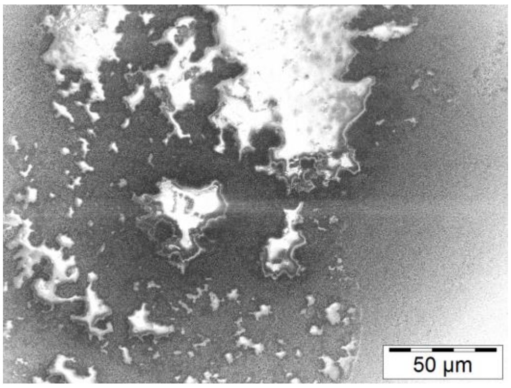

Figure S38. SEM micrograph of a pristine **X2-PNMPT**-based electrode at 500× magnification.

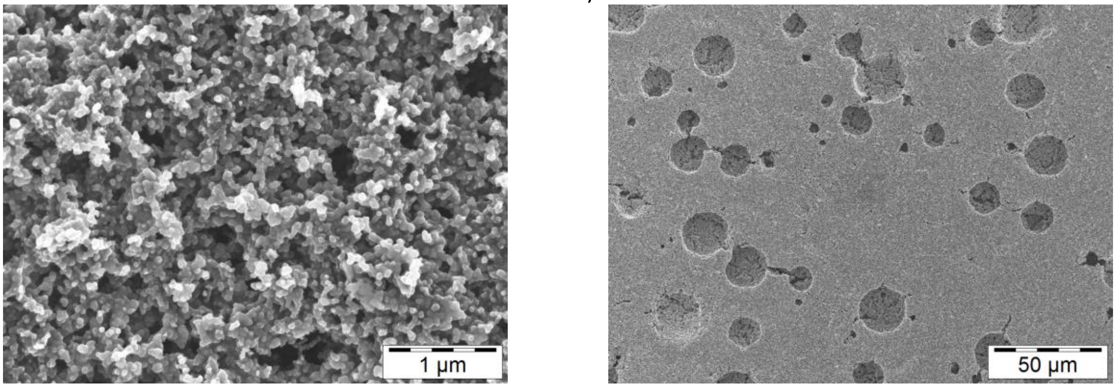

Figure S39. SEM micrographs of a pristine **P1**-based electrode at a) 25000× magnification and b) 500× magnification.

a) b)

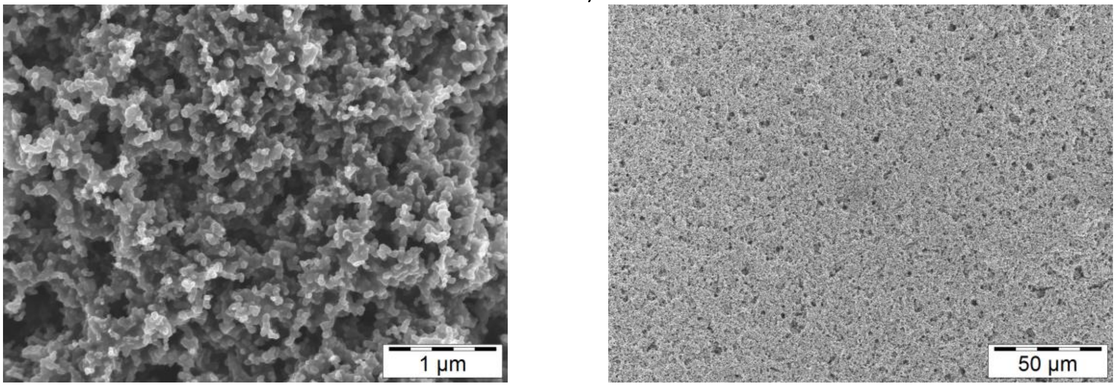

Figure S40. SEM micrographs of a pristine **X1-P1**-based electrode at a) 25000× magnification and b) 500× magnification.

a) b)

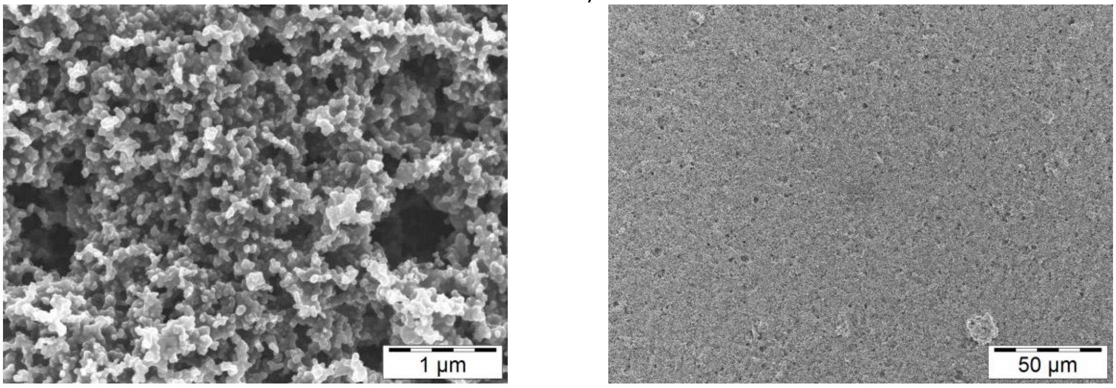

Figure S41. SEM micrographs of a pristine **X2-P1**-based electrode at a) 25000× magnification and b) 500× magnification.

a) b)

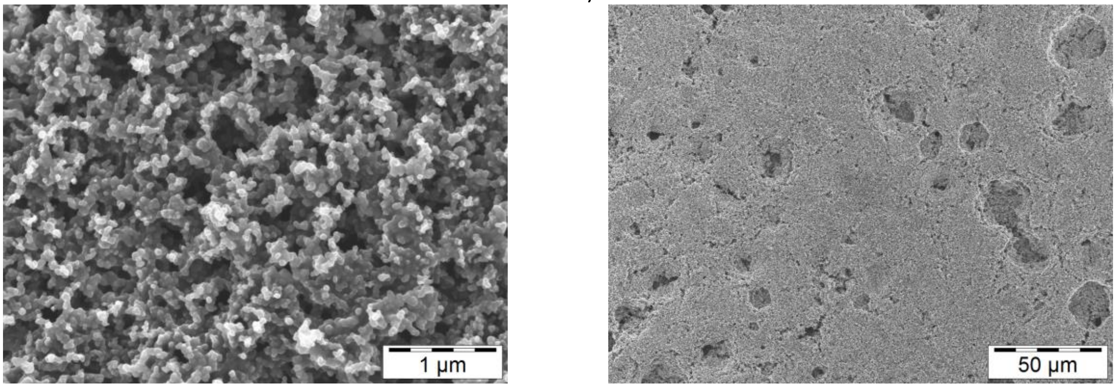

Figure S42. SEM micrographs of a pristine **P2**-based electrode at a) 25000× magnification and b) 500× magnification.

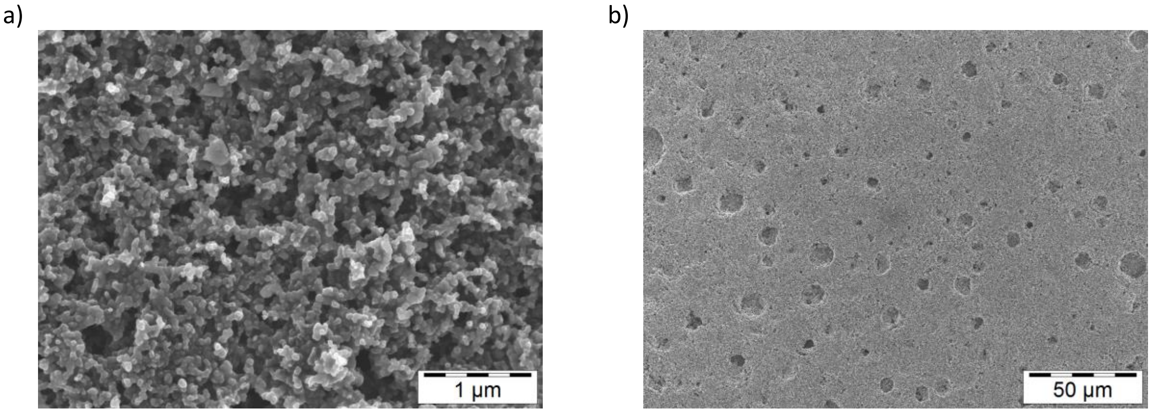

Figure S43. SEM micrographs of a pristine **X1-P2**-based electrode at a) 25000× magnification and b) 500× magnification.

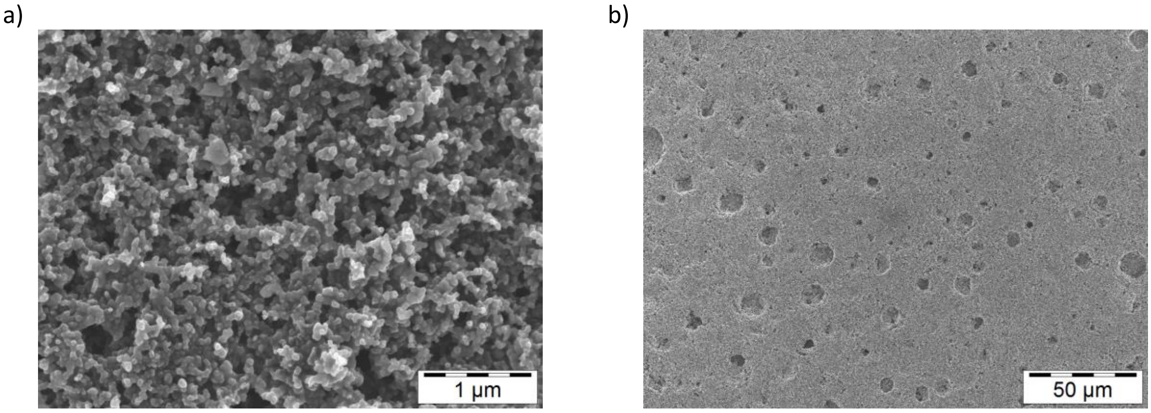

Figure S44. SEM micrographs of a pristine **X2-P2**-based electrode at a) 25000× magnification and b) 500× magnification.

3.3 Electrochemical analyses

Electrochemical experiments were performed using an adapted *Swagelok*® three-electrodes cell setup. All the cells were assembled in a dry room with less than 0.02% air moisture. The previously fabricated polymer-based electrodes were used as working electrode (WE, Ø = 12 mm). Li-metal was used as reference electrode (RE, Ø = 5 mm) and counter electrode (CE, Ø = 12 mm). Six layered FREUDENBERG *2190* separators were placed between the WE and CE (Ø = 13 mm) and at the RE (Ø = 10 mm) and soaked with 130 µL and 60 µL of electrolyte (1 M $LiPF_6$ in EC:DMC, 1: 1, BASF *Selectilyte*), respectively. Prior to use, the separators were dried in a vacuum oven (10^{-3} mbar, 120 °C, 48 h). BoPET foil (PPI ADHESIVE PRODUCTS, 100 µm) was used as an insulator to avoid any contact between the internal components and the stainless-steel cell body.

Cyclic voltammetry measurements were conducted on a *VMP3* potentiostat (Biologic Science Instruments), and constant current cycling investigations were performed on a MACCOR *4000 Series* battery cycler. All electrochemical measurements on test cells were conducted in climatic chambers at 20 °C.

3.3.1 Cyclic voltammetry investigations of MPT poly(norbornene)-based electrodes

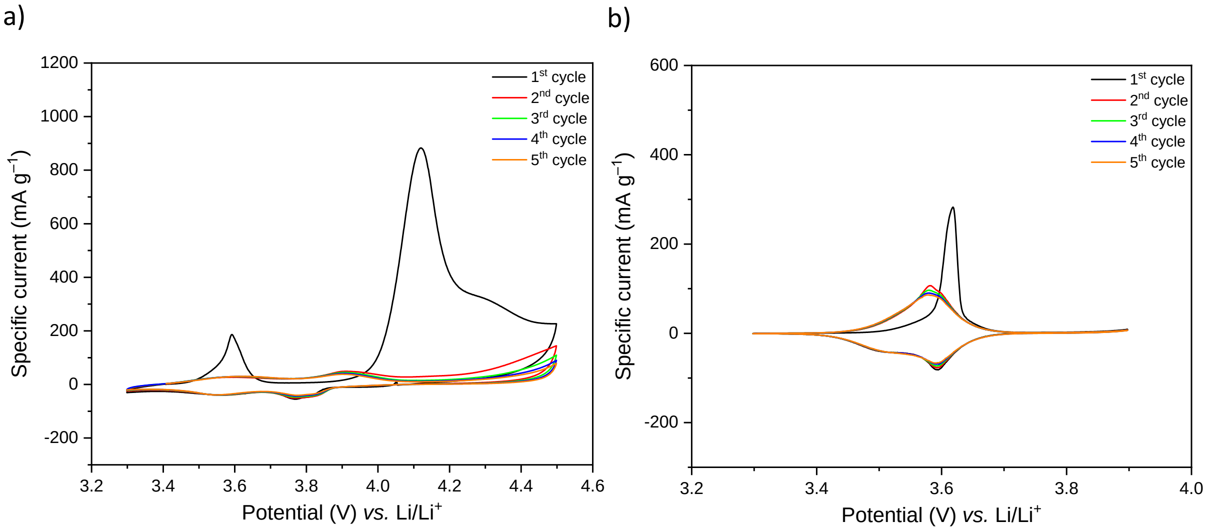

Figure S45. Cyclic voltammograms of a) **PNMPT**- (including second oxidation) and b) **X-PNMPT**-based electrodes. Scan rate: 50 µV s^{-1}.

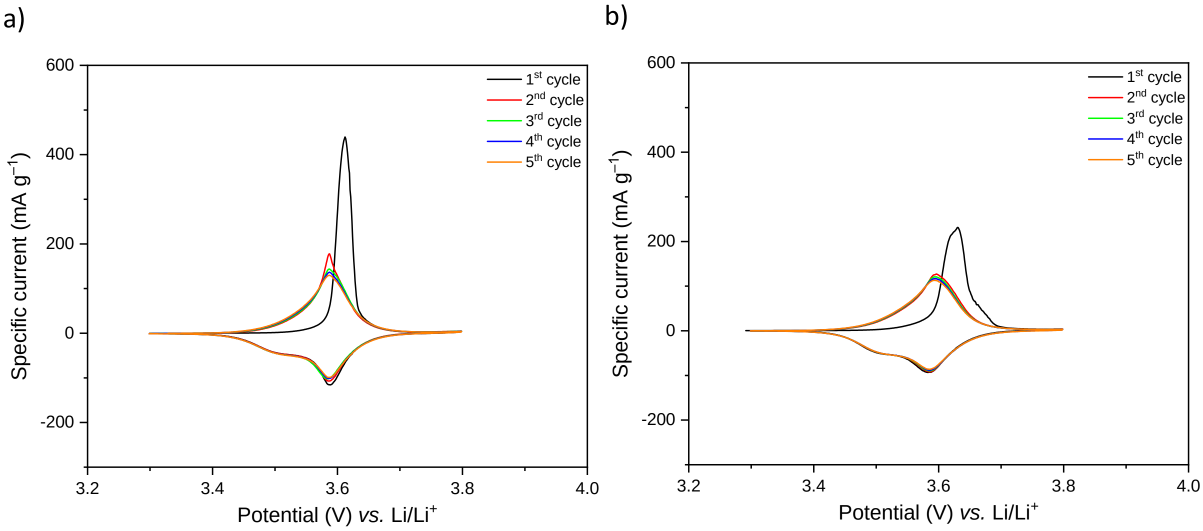

Figure S46. Cyclic voltammograms of a) **X1-PNMPT**- and b) **X2-PNMPT**-based electrodes. Scan rate: 50 µV s^{-1}.

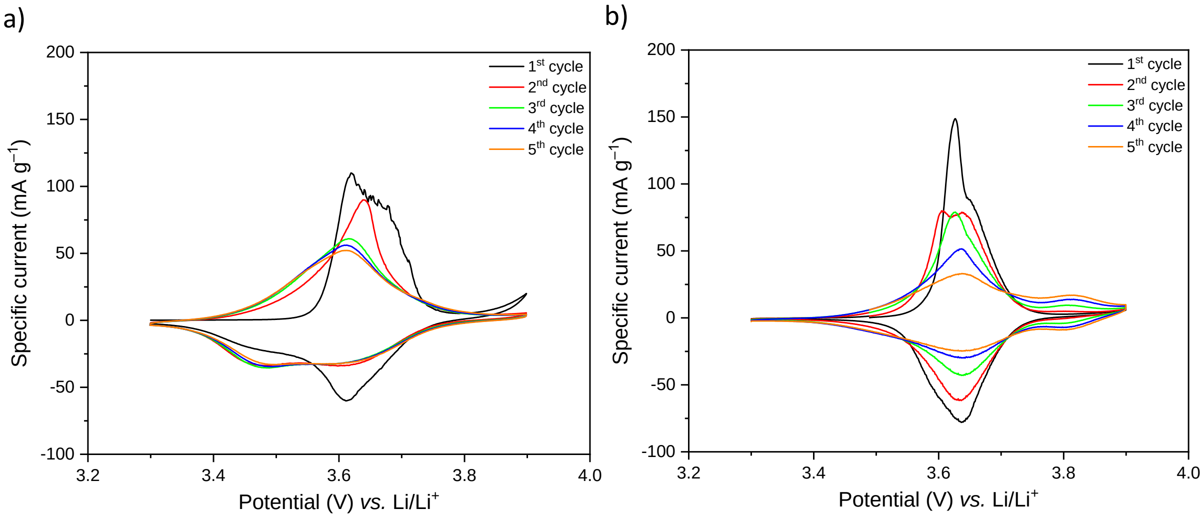

Figure S47. Cyclic voltammograms of a) **X1-P1**- and b) **X2-P1**-based electrode. Scan rate: 50 µV s^{-1}.

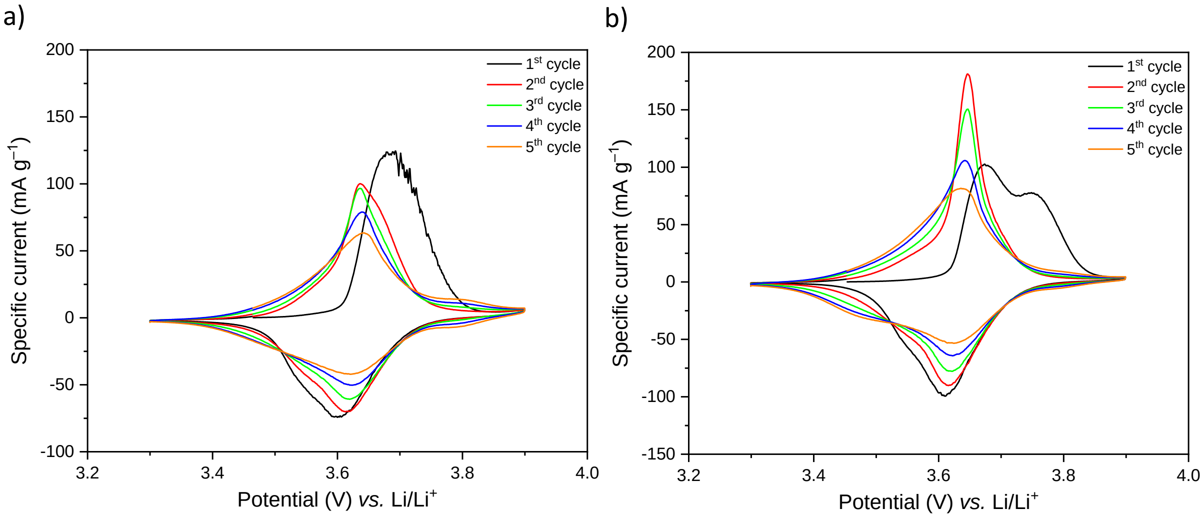

Figure S48. Cyclic voltammograms of a) **X1-P2**- and b) **X2-P2**-based electrodes. Scan rate: 50 µV s^{-1}.

3.3.2 Constant current cycling and rate capability tests of MPT poly(norbornene)-based electrodes

a) b)

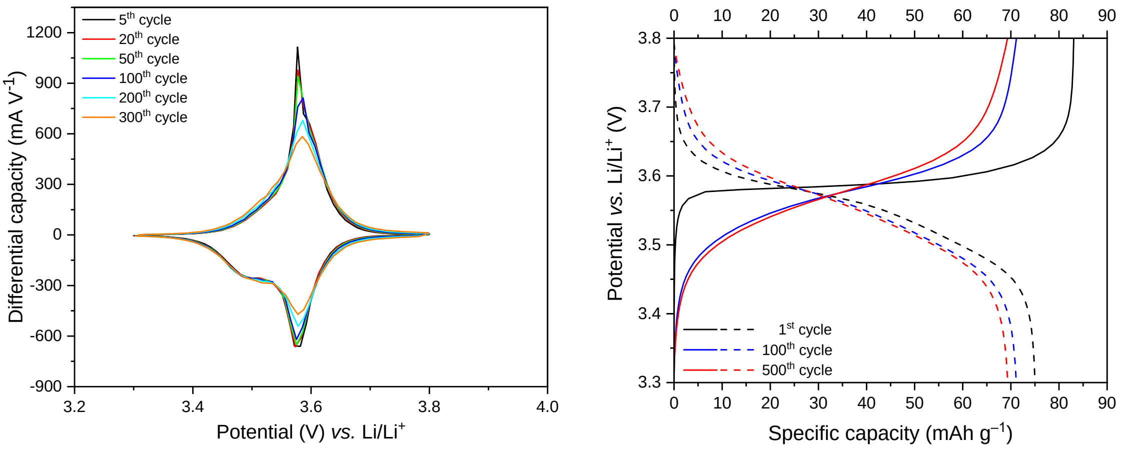

Figure S49. a) Differential capacity plot and b) charge/discharge profiles from constant current cycling measurement of a **PNMPT**-based electrode at 1C.

a) b)

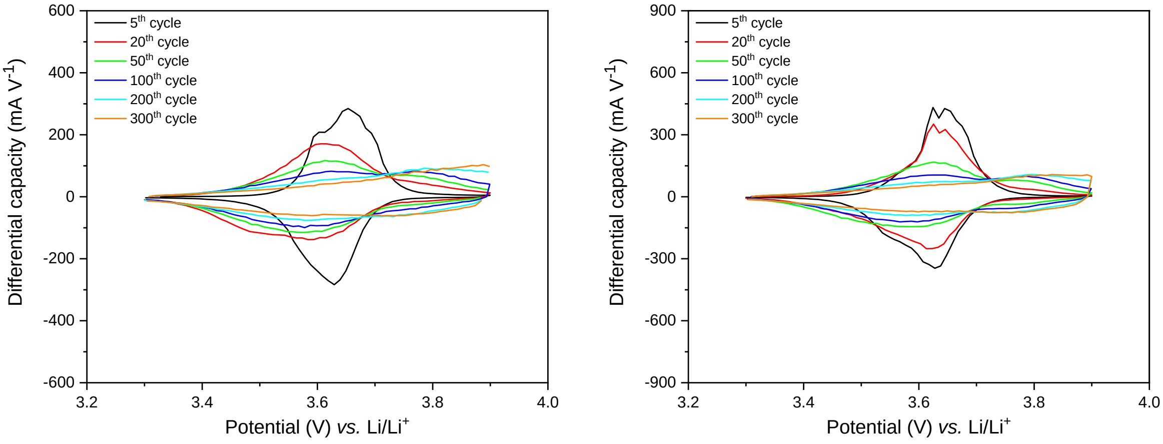

Figure S50. Differential capacity plot of a) a **P1**-based electrode and b) a **P2**-based electrode from constant current cycling measurements at 1C.

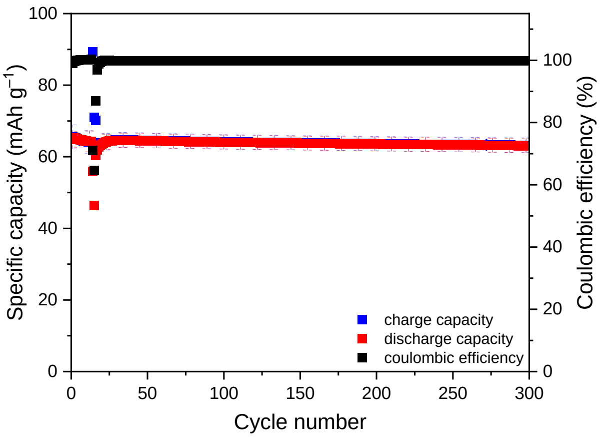

Figure S51. Constant current cycling measurements of **X-PNMPT**-based electrodes at 1C. Potential range: 3.3–3.9 V *vs.* Li/Li$^+$ (average of three measurements shown with error bars). Due to maintenance on the cycling device, the procedure was temporarily interrupted for several hours. A rest step of undetermined duration leads to an inconsistency around cycle 15.

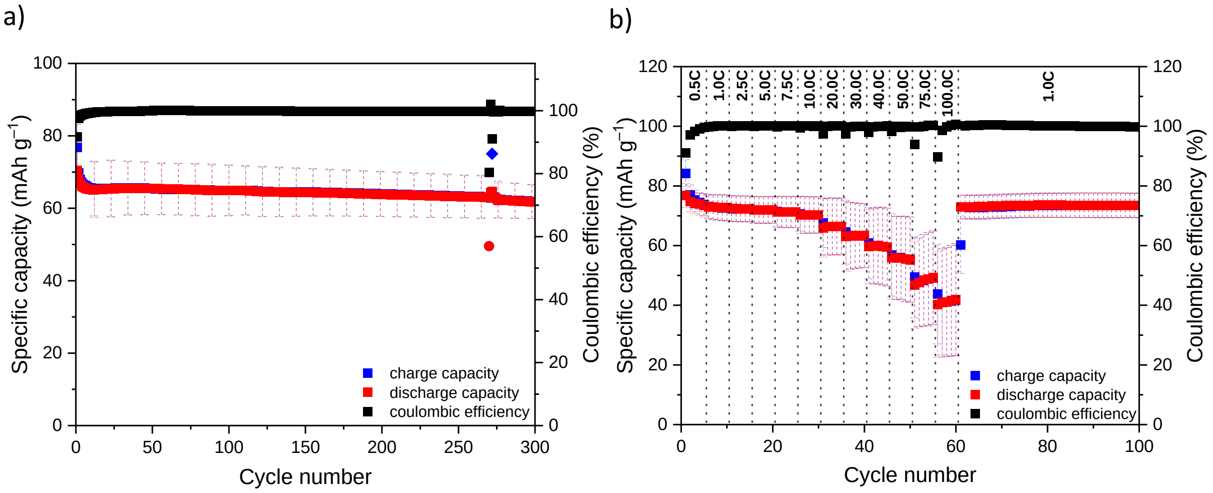

Figure S52. a) Constant current cycling measurements at 1C and b) rate performance measurements of **X1-PNMPT**-based electrodes. Potential range: 3.3–3.8 V *vs.* Li/Li$^+$ (average of three measurements shown with error bars). Due to maintenance on the cycling device, the procedure was temporarily interrupted for several hours. A rest step of undetermined duration leads to an inconsistency around cycle 270.

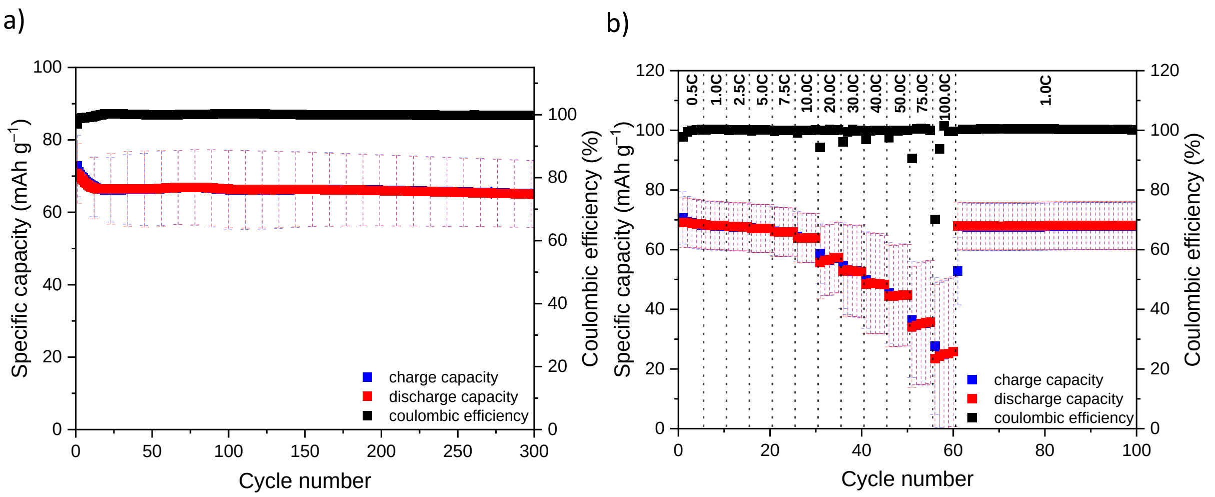

Figure S53. a) Constant current cycling measurements at 1C and b) rate performance measurements of **X2-PNMPT**-based electrodes. Potential range: 3.3–3.8 V *vs.* Li/Li^+ (average of three measurements shown with error bars).

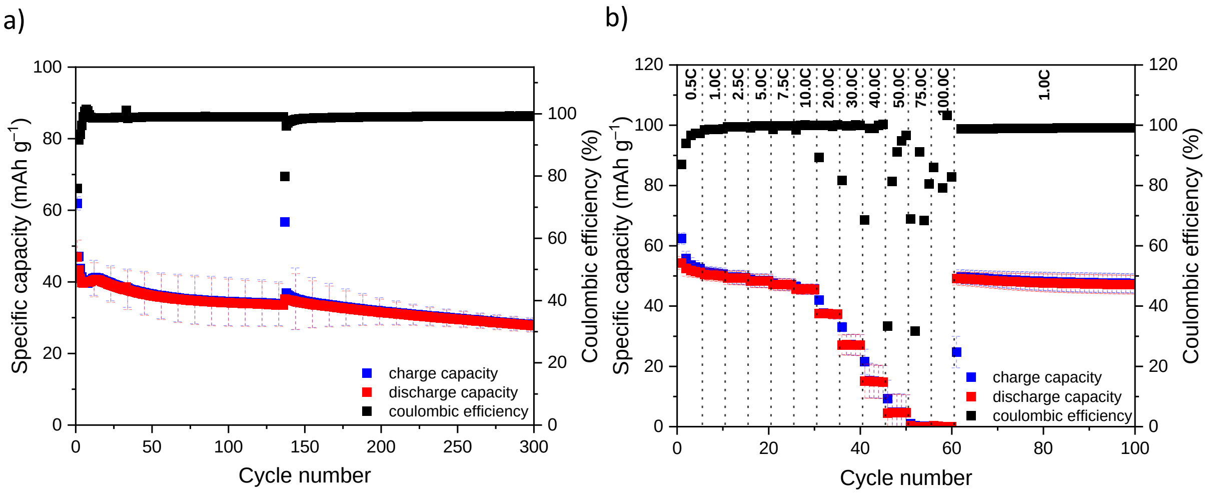

Figure S54. a) Constant current cycling measurements at 1C and b) rate performance measurements of **P1**-based electrodes. Potential range: 3.3–3.9 V *vs.* Li/Li^+ (average of three measurements shown with error bars). Due to maintenance on the cycling device, the procedure was temporarily interrupted for several hours. A rest step of undetermined duration leads to an inconsistency around cycle 140.

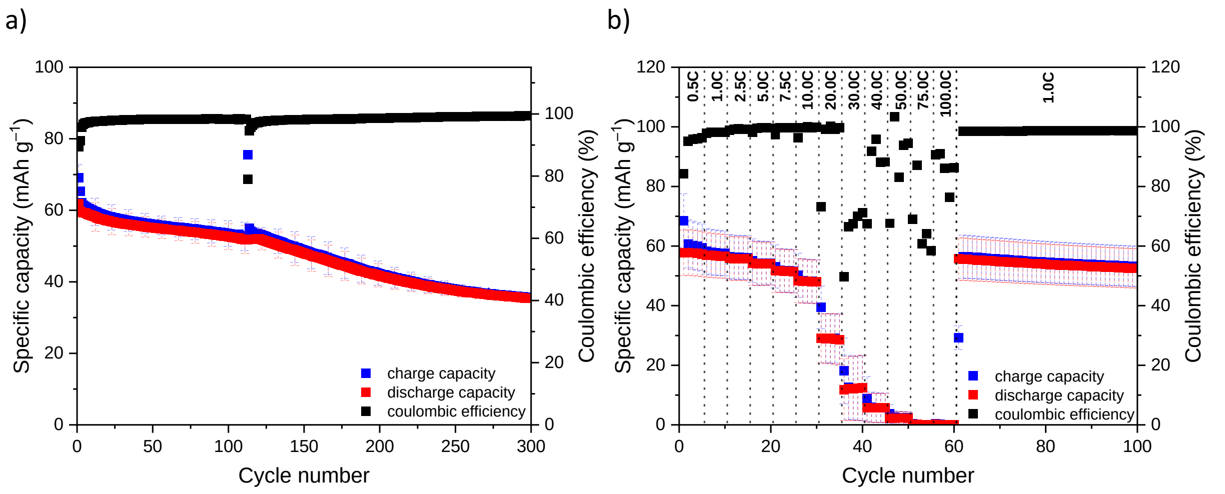

Figure S55. a) Constant current cycling measurements at 1C and b) rate performance measurements of **X1-P1**-based electrodes. Potential range: 3.3–3.9 V *vs.* Li/Li^{+} (average of three measurements shown with error bars). Due to maintenance on the cycling device, the procedure was temporarily interrupted for several hours. A rest step of undetermined duration leads to an inconsistency around cycle 115.

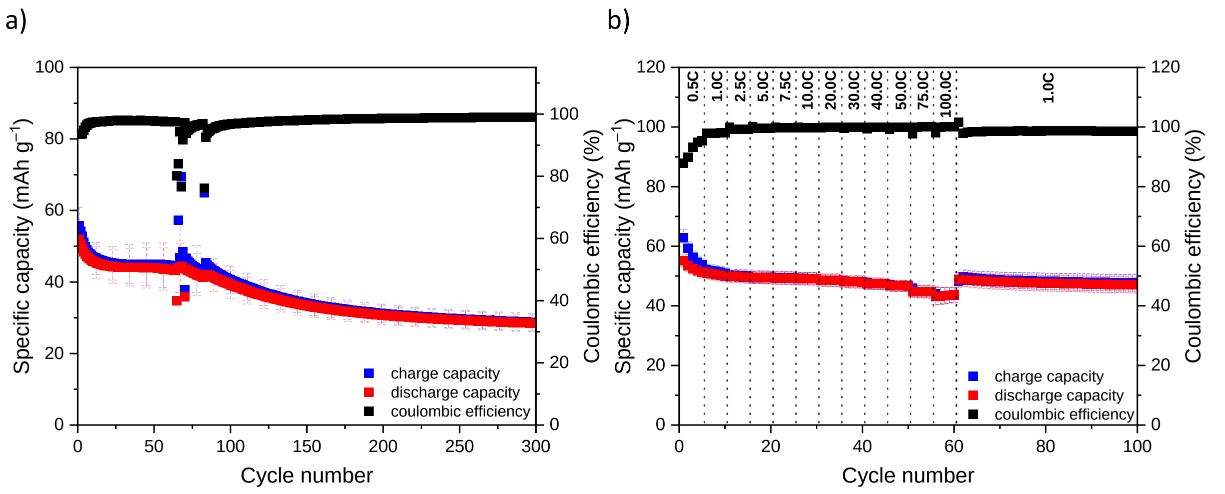

Figure S56. a) Constant current cycling measurements at 1C and b) rate performance measurements of **X2-P1**-based electrodes. Potential range: 3.3–3.9 V *vs.* Li/Li^{+} (average of three measurements shown with error bars). Due to maintenance on the cycling device, the procedure was temporarily interrupted for several hours. A rest step of undetermined duration leads to an inconsistency around cycle 70.

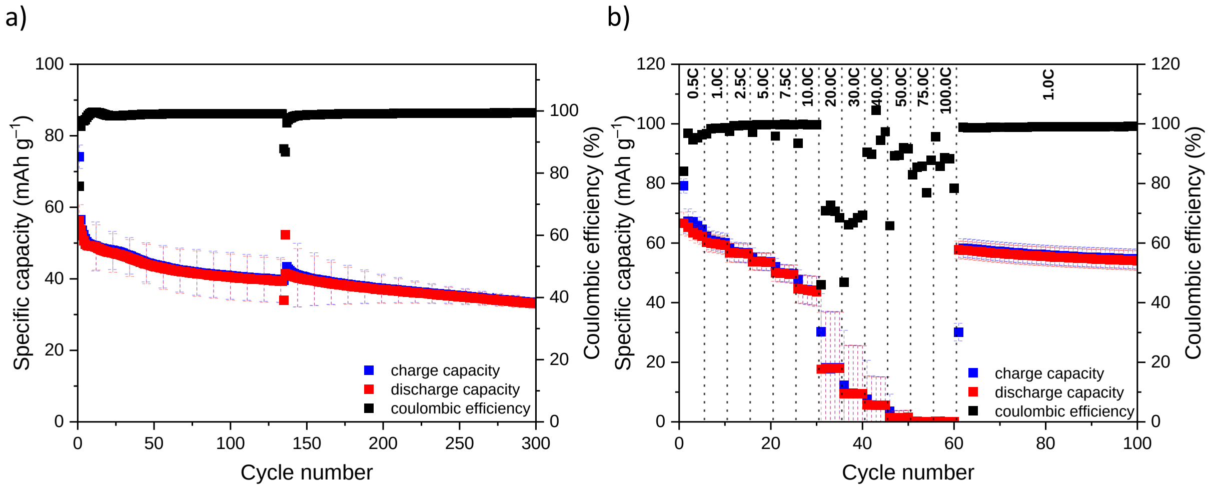

Figure S57. a) Constant current cycling measurements at 1C and b) rate performance measurements of **P2**-based electrodes. Potential range: 3.3–3.9 V *vs.* Li/Li^+ (average of three measurements shown with error bars). Due to maintenance on the cycling device, the procedure was temporarily interrupted for several hours. A rest step of undetermined duration leads to an inconsistency around cycle 140.

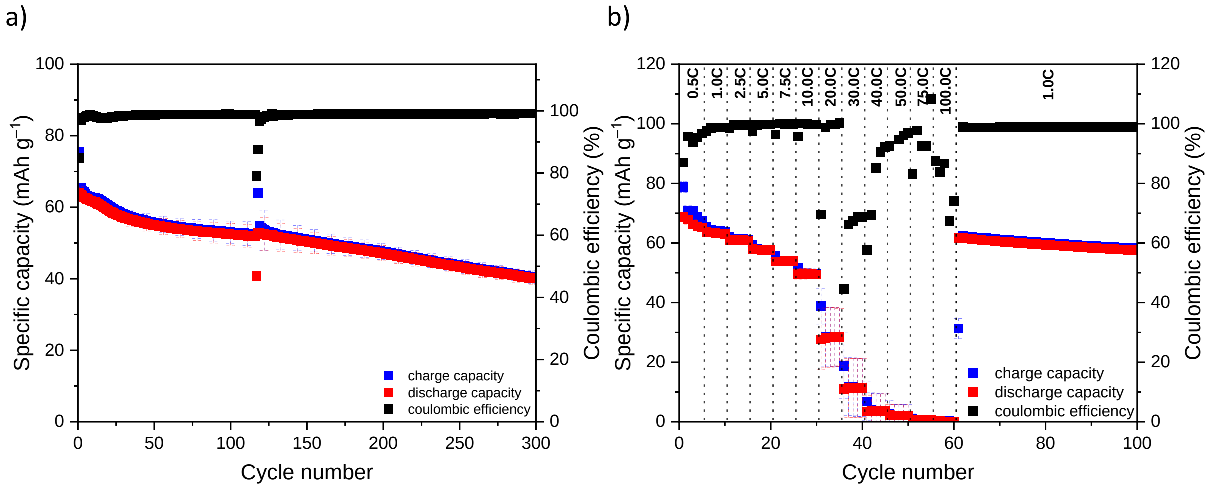

Figure S58. a) Constant current cycling measurements at 1C and b) rate performance measurements of **X1-P2**-based electrodes. Potential range: 3.3–3.9 V *vs.* Li/Li^+ (average of three measurements shown with error bars). Due to maintenance on the cycling device, the procedure was temporarily interrupted for several hours. A rest step of undetermined duration leads to an inconsistency around cycle 120.

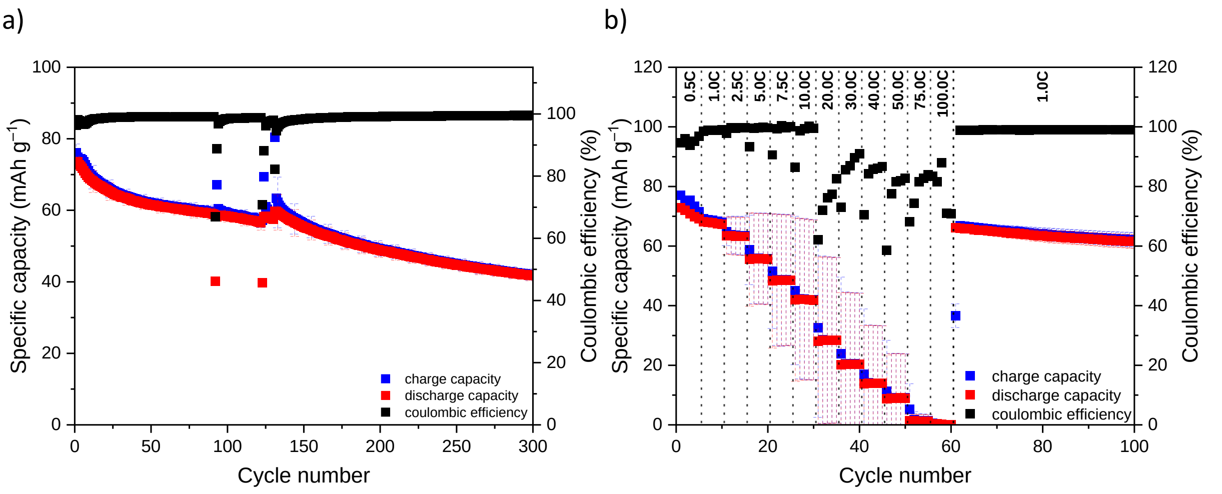

Figure S59. a) Constant current cycling measurements at 1C and b) rate performance measurements of **X2-P2**-based electrodes. Potential range: 3.3–3.9 V *vs.* Li/Li^+ (average of three measurements shown with error bars). Due to maintenance on the cycling device, the procedure was temporarily interrupted for several hours. Rest steps of undetermined duration leads to inconsistencies around cycle 90 and 125.

3.3.3 Constant current cycling of a *Super C65*-based electrode for comparison

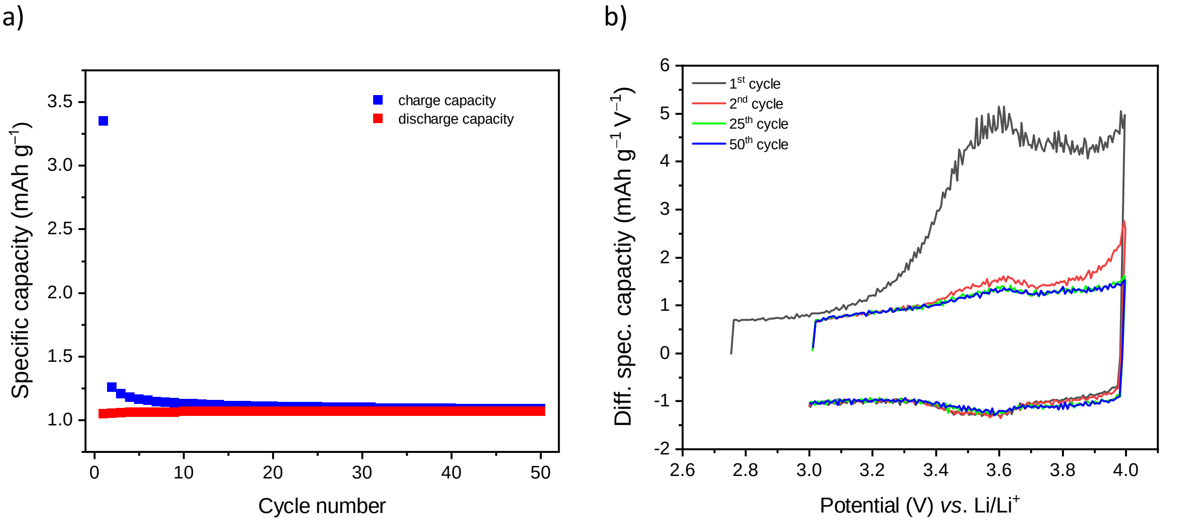

Figure S60. a) Constant current cycling measurement of a composite electrode containing 80 wt% *Super C65* and 20 wt% PVdF performed at 1C in a potential range of 3.0–4.0 V *vs.* Li/Li^+ and b) its differential specific capacity plot.

4 References

[1] M. Kolek, F. Otteny, P. Schmidt, C. Mück-Lichtenfeld, C. Einholz, J. Becking, E. Schleicher, M. Winter, P. Bieker, B. Esser, *Energy Environ. Sci.* **2017**, *10*, 2334–2341.

[2] F. Otteny, M. Kolek, J. Becking, M. Winter, P. Bieker, B. Esser, *Adv. Energy Mater.* **2018**, *8*, 1802151.

[3] S. A. N. Hashmi, X. Hu, C. E. Immoos, S. J. Lee, M. W. Grinstaff, *Org. Lett.* **2002**, *4*, 4571–4574.

[4] T. Ito, Y. Hamaguchi, K. Tanabe, H. Yamada, S. I. Nishimoto, *Angew. Chemie - Int. Ed.* **2012**, *51*, 7558–7561.

[5] A. G. M. Barrett, B. T. Hopkins, A. C. Love, L. Tedeschi, *Org. Lett.* **2004**, *6*, 835–837.

[6] J. K. Pontrello, M. J. Allen, E. S. Underbakke, L. L. Kiessling, *J. Am. Chem. Soc.* **2005**, *127*, 14536–14537.
